# Animal tracking with particle algorithms informs protected area design

**DOI:** 10.1126/sciadv.adx0255

**Published:** 2025-11-26

**Authors:** Edward Lavender, Andreas Scheidegger, Carlo Albert, Stanisław W. Biber, Jakob Brodersen, Dmitry Aleynik, Georgina Cole, Jane Dodd, Peter J. Wright, Janine Illian, Mark James, Sophie Smout, James Thorburn, Helen Moor

**Affiliations:** ^1^ Department of Systems Analysis, Integrated Assessment and Modelling, Eawag Swiss Federal Institute of Aquatic Science and Technology, Dübendorf, Zürich, Switzerland.; ^2^ School of Mathematics and Physics, University of Surrey, Guildford, Surrey, United Kingdom.; ^3^ Department of Fish Ecology and Evolution, Eawag Swiss Federal Institute of Aquatic Science and Technology, Kastanienbaum, Luzern, Switzerland.; ^4^ Scottish Association for Marine Science, Oban, Argyll and Bute, United Kingdom.; ^5^ Royal Zoological Society of Scotland, Edinburgh, Midlothian, United Kingdom.; ^6^ School of Applied Sciences, Edinburgh Napier University, Edinburgh, Midlothian, United Kingdom.; ^7^ NatureScot, Oban, Argyll and Bute, United Kingdom.; ^8^ Marine Scotland Science, Aberdeen, Aberdeenshire, United Kingdom.; ^9^ School of Mathematics and Statistics, University of Glasgow, Glasgow, Lanarkshire, United Kingdom.; ^10^ Scottish Oceans Institute, University of St Andrews, St Andrews, Fife, United Kingdom.; ^11^ Centre for Conservation and Restoration Science, Edinburgh Napier University, Edinburgh, Midlothian, United Kingdom.

## Abstract

Animal movements affect their exposure to threats and the efficacy of conservation measures, such as marine protected areas (MPAs). However, many species’ movements are difficult to reconstruct from available datasets, hampering conservation efforts. This is especially the case for aquatic species that rarely surface, for which data are often limited to observations from acoustic telemetry (detections) and ancillary sensors. Here, we pioneer the use of state-of-the-art particle algorithms to model movements, integrate datasets, and assess MPA design, leveraging a case study of a Critically Endangered elasmobranch. Our algorithms led to 5-fold improvements in space-use maps and 30-fold improvements in residency estimates compared to prevailing methods. By integrating tracking datasets, we were uniquely able to examine movements beyond acoustic receivers, MPA-scale residency, and specific habitats beyond protected areas that warrant protection. This work reveals a modeling framework that enhances the conservation value of acoustic telemetry, supporting analyses of MPA efficacy worldwide.

## Teaser

Particle algorithms strengthen the value of acoustic telemetry for conservation.

## INTRODUCTION

Ocean biodiversity is increasingly threatened by anthropogenic activities, such as overfishing (*1*). Many marine taxa have declined, especially in coastal ecosystems (*2*). In some taxa, such as elasmobranchs (sharks, skates, and rays), rates of decline are now critical (*3*, *4*). There is a pressing need for research designed to inform conservation measures that can support these species and bend the curve of aquatic biodiversity decline (*5*).

Marine protected areas (MPAs) are an important conservation solution (*6*). An effective MPA is a refuge that reduces the pressures to which individuals are exposed (especially fishing) and supports population recovery (*7*, *8*). Designing MPAs for mobile species requires an understanding of animal movement, which shapes individual exposure to threats, hotspots of habitat use, and residency in selected areas (*9*). This requirement has motivated huge interest in animal electronic tagging and tracking (*10*).

Tagging and tracking technologies for aquatic species have proliferated in recent years (*11*). For marine mammals and seabirds, satellite transmitters are widely used (*12*, *13*). These tags periodically collect/transmit location data from which movement trajectories can be reconstructed using well-established statistical approaches (*14*). However, for species that rarely surface, satellite tracking is limited and alternative technologies are required (*11*). Passive acoustic telemetry systems are extensively deployed (*15*). These use receiver arrays to detect individual-specific acoustic transmissions of tagged animals when they move within range. Since array coverage is often limited, detections are usually sparse and may be considerably enhanced by ancillary datasets, such as archival (e.g., depth) observations (*16*). However, integrating sparse detections with ancillary datasets to reconstruct movement patterns (within and beyond receiver arrays) remains a substantial challenge that has hampered the use of these data to inform MPA design.

Heuristic methods currently dominate efforts to analyze movements in passive acoustic telemetry systems (*17*–*19*). These methods use detections and apply summary statistics and tuning parameters to map space use around receivers (*17*). For example, the center-of-activity (COA) algorithm computes weighted averages of the receiver locations where detections were recorded over sequential time intervals (of duration ΔT ) (*20*, *21*). Similarly, the refined shortest path (RSP) algorithm interpolates “relocations” along the shortest paths between the receivers that recorded sequential detections, assuming distance-dependent interpolation errors (in line with a tuning parameter termed er.ad) (*22*). Post hoc smoothing is used to generate maps of space use or utilization distributions (UDs). Residency indices (such as “detection days” or the proportion of days with detections) have also been developed to quantify residency around receivers (*23*). These methods have been subject to limited formal evaluation, but their limitations in sparse receiver arrays (where individual movements are uncertain) are acknowledged (*17*–*19*).

Recent developments in state-space modeling create opportunities to move beyond heuristics and strengthen animal-tracking analyses for conservation (*19*, *24*). In an animal-tracking context, a state-space model (SSM) is a hierarchical framework that models an underlying movement process and the observation processes that connect movements to observations (*25*). Until recently, state-space modeling routines for passive acoustic telemetry data were bespoke, computationally expensive, and limited to detection data (*24*, *26*, *27*). However, it is now possible to fit SSMs that integrate detections and diverse ancillary datasets (from sensor measurements to mark-recapture events) using particle filtering–smoothing algorithms (*19*, *28*). As examples, this paper considers algorithms that incorporate acoustic observations, depth observations, or both sets of observations simultaneously; we label these algorithms the “acoustic-container” (AC), “depth-contour” (DC), and “acoustic-container depth-contour” (ACDC) algorithms (*19*). These algorithms represent an individual’s possible location probabilistically with a set of weighted samples, termed “particles.” A simulation study showed that particle algorithms consistently outperform heuristic methods, generating refined maps of space use and residency estimates for entire regions of interest, such as MPAs (*19*). However, particle algorithms have yet to be exploited to inform MPA design in any real-world system.

**Fig. 1.**
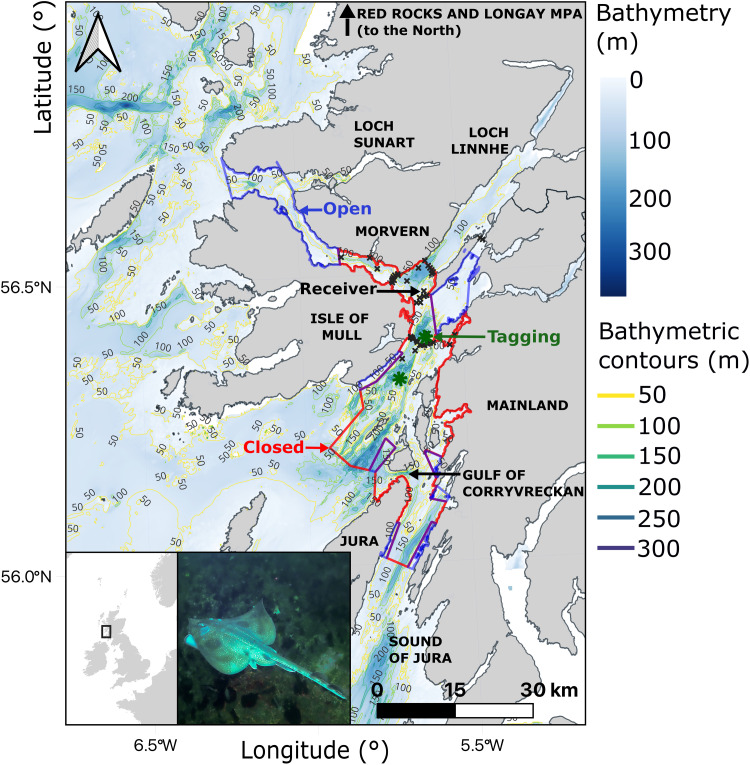
A case-study system. The insets show the location of the study system in the United Kingdom and a flapper skate. The main panel shows the study area. The colored polygons mark the boundary of the Loch Sunart to the Sound of Jura MPA (741 km^2^). This includes zones that are open (blue) and closed (red) to fisheries. Tagging locations (✵), receivers (x), and bathymetric contours are marked. For spatial data sources, see text S1 and table S1. Photo credit: Simon Bradley.

Here, we pioneer the application of particle algorithms for conservation with a case study of the Critically Endangered flapper skate (*Dipturus intermedius*) (Fig. 1). This is a large-bodied, largely benthic species that occupies habitats from 0 to 1200 m deep in north-western Europe (*29*). Once decimated by overfishing (*30*), flapper skate remain vulnerable as bycatch (*31*). In 2014, the Loch Sunart to the Sound of Jura MPA was designated for flapper skate in Scotland. To monitor skate in the MPA, an acoustic array comprising 58 receivers was later established (Fig. 1). Skate were tagged with acoustic transmitters (programmed to transmit an individual-specific signal every 30 to 90 s) and archival tags (programmed to record depth every 2 min) (*32*–*34*). Preliminary analyses demonstrated that skate exhibit localized movements within the MPA (*32*, *35*, *36*). However, key conservation questions pertaining to MPA efficacy—including the use of seasonally fished zones beyond receivers, the extent to which skate remain in the MPA in detection gaps, and the suitability of MPA boundaries—remained poorly addressed, given incomplete receiver coverage. Movements beyond the MPA, and connectivity to the only known egg nursery (in the Red Rocks and Longay MPA), also remained poorly understood (*37*). Such knowledge gaps are pervasive among studies reliant upon sparse receiver arrays (*38*, *39*) and highlight the need for statistical tools that can enhance the conservation of mobile aquatic species.

Combining simulations and real-world analyses, this study showcases how particle algorithms can reveal marked improvements in maps of space use, residency in regions beyond receivers, MPA suitability, and specific habitats beyond MPA boundaries where additional protection may be warranted. By comparing maps based on acoustic and/or archival data, we also quantify the contributions of different datasets, providing valuable information for monitoring programs. This work provides a basis to strengthen the use of movement data for mobile species conservation across the globe.

## RESULTS

### Overview

We conducted simulation-based and real-world analyses of animal movements in relation to an MPA using two heuristic algorithms and three particle filtering–smoothing algorithms (Fig. 1). The two heuristic algorithms (COAs and RSPs) incorporated detections; the particle algorithms (AC, DC, and ACDC) fitted SSMs incorporating acoustic observations (the AC algorithm), depth observations (the DC algorithm), or both datasets (the ACDC algorithm). For a summary of the differences among algorithms, see Table 1. For a visualization of the SSM, see fig. S1. In simulation analyses (labeled A1 and A2), we compared simulated and reconstructed patterns to evaluate algorithm performance (A1) and sensitivity (A2). In real-world analyses (A3 and A4), we modeled tracking data from flapper skate (fig. S2). Table S2 provides an overview and the labeling hierarchy for analyses (A1, A1.1, etc.). Figure S3 provides a visual summary.

**Table 1.** Summary of algorithms. We considered five algorithms: center of activity (COA), refined shortest path (RSP), and the acoustic-container (AC), depth-contour (DC), and acoustic-container depth-contour (ACDC) particle algorithms. The table highlights their main features, including algorithm type, structural components, inputs, and outputs. Selected analyses also included a null model and a detection days (DD) residency metric for comparison.

Attribute	Category	Algorithm				
		COA	RSP	AC	DC	ACDC
Algorithm type	Heuristic	✓	✓			
	State-space model–based (particle algorithm)			✓	✓	✓
Structure	Movement model			✓	✓	✓
	Observation model(s)			✓	✓	✓
Inputs	Detections	✓	✓	✓		✓
	Nondetections			✓		✓
	Depth observations				✓	✓
Outputs	Interpolated positions	✓	✓			
	Particle samples			✓	✓	✓
	Uncertainty quantification			✓	✓	✓

### Simulation analysis

#### Performance analyses

In simulation analyses of algorithm performance (A1), particle algorithms reproduced the UDs and residency patterns exhibited by 100 simulated paths with impressive accuracy and precision, while the performance of prevailing heuristic methods varied. Heuristic algorithms were calibrated for the analysis by quantifying the mean absolute error (ME) between “true” and reconstructed UDs across a range of candidate parameter values (A1.1.1). For the COA algorithm, an optimal setting of ΔT=2days was identified (fig. S4). For RSPs, ME weakly declined with increasing distance-error (er.ad) values but there was no winning choice of er.ad on average. We selected er.ad = 500 m as an “optimal” compromise between ME and implementation success (fig. S4). Particle algorithms were parameterized following the data-generating movement and observation processes used to simulate paths, based on previous research, domain knowledge, and a literature review (see Materials and Methods). All algorithms were successfully implemented (A1.2) for all simulated paths (fig. S5).

In a visual analysis of UDs for a subset (1 to 3) of simulated paths (A1.3.1), we found that heuristic UDs were moderately accurate but surpassed by those from particle algorithms (figs. S6 and S7 versus fig. S8). Both heuristic algorithms effectively reconstructed the extent of movements for path 3 (for which movements were localized around receivers) but underestimated those for paths 1 and 2 (which included movements to different areas) (figs. S6 and S7). Hotspot placement was partially correct (figs. S6 and S7). Particle algorithms represented simulated patterns more accurately (fig. S8).

**Fig. 2.**
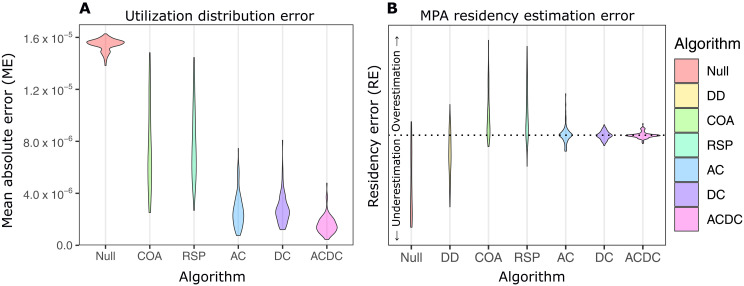
Algorithm performance for estimating UDs and residency. Violins show (**A** and **B**) the distribution of error across 100 simulated paths. See text for abbreviations. For the full figure, see fig. S9.

Across all 100 simulated paths, a clear ranking of algorithms emerged from analysis (A1.3.2) of the ME in UD estimation (Fig. 2A). All algorithms outperformed the null model (a flat UD defined independently of the observations) on average. Performance of the two heuristic algorithms was similar and varied substantially across repeated realizations of the same data-generating processes (occasionally overlapping with the null model). Particle algorithms consistently outperformed the heuristic algorithms—with median MEs, and SDs in ME, 2.7 to 5.0 and 1.7 to 3.6 times lower, respectively. For particle algorithms, the ranking was DC, AC, and ACDC (best). Compared to the AC and DC algorithms, median ME for the ACDC algorithm was 1.6 to 1.8 times lower.

Algorithm ranking for residency estimation (A1.3.3) was similar (Fig. 2B and fig. S9). The null model consistently underestimated residency. The proportion of days with detections (DD) benchmark also underestimated residency, while heuristic algorithms overestimated residency, except in open areas where receivers were absent. For heuristic algorithms, median residency error for the MPA was 12% (SE ≈ 22%). Particle algorithms performed better, estimating residency in the MPA with a median error below 1% and a precision (SE) of 7.6% (AC), 3.7% (DC), and 2.8% (ACDC). These statistics represent >30-fold improvements in residency error and 2.8- to 8.3-fold improvements in precision compared to heuristic algorithms.

#### Sensitivity analysis

The sensitivity analyses (A2) revealed algorithm sensitivity to mis-specification. We reimplemented algorithms with more restrictive and flexible parameterizations and examined variation in performance. Heuristic algorithms were successfully re-implemented for all simulated paths (A2.1). For particle algorithms, parameter mis-specification was associated with convergence failures (fig. S5). The restrictive depth observation model, which underestimated the amount of error in the depth observations, was particularly problematic. However, multiple independent runs of the particle filter facilitated convergence in some simulations.

Patterns of space use and residency estimates (A2.2) were relatively robust to algorithm parameterization. The visual analysis (A2.2.1) revealed consistency in UDs between algorithm parameterizations (figs. S6, S7, S10, and S11). For heuristic algorithms, more flexible parameterizations produced more diffuse maps, but differences were small (figs. S6 and S7). For the particle algorithms that converged, maps were relatively robust to the degree of parameter mis-specification we explored (figs. S10 and S11). The main exception to this was mis-specification of the depth observation model (in the DC and ACDC algorithms), which affected the distribution of hotspots and patterns of space use (fig. S10). Particle-based maps were highly reproducible (fig. S11). Analyses of UD ME (A2.2.2) and residency (A2.2.3) produced similar results (figs. S12 and S13).

### Real-world analysis

#### Observations

Real tracking data from modeled skate exhibited diverse patterns (fig. S2). The number of detections per individual/month varied from 43 to 8887 (median = 1152). Some individuals were detected regularly, while others went undetected for longer periods (up to 25 days). Most individuals were only detected around southerly receivers. The two exceptions were individual 25 (a mature female), which moved between southern and northern receivers over a 14-month period, and individual 28 (an immature female), which was primarily detected at southerly receivers but spent some time around northerly receivers in early 2017. Depth time series were variable and included extended periods (>1 week) with limited change alongside movements from a maximum depth (≈150 m) into shallower water and extensive transitions between shallow (<50 m) and deep (>200 m) areas (fig. S2).

#### Main analyses

In our main analyses (A3), algorithms were successfully implemented (A3.1) for most individuals/months (fig. S14). The success rate was high for the heuristic algorithms (98 to 100%). In the particle algorithms, the convergence rate was high for the AC algorithm (100%), but lower in the DC (79%) and ACDC (85%) algorithms.

**Fig. 3.**
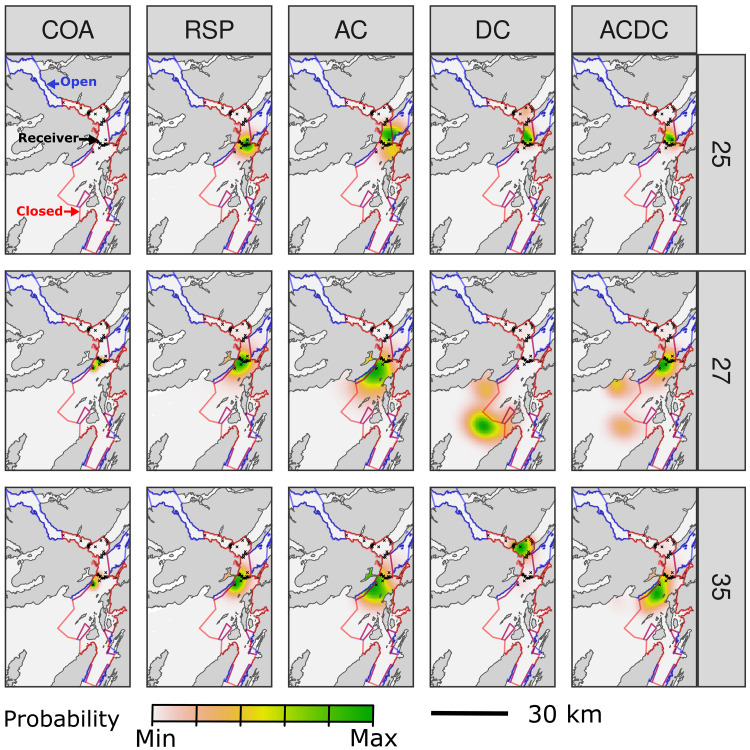
Example UDs from tagged skate. Each panel shows a UD for April 2016 for a given individual (row) and algorithm (column). (UDs for the null model are not shown, as they are uniform over the marine area.) Receivers (x) and zones open/closed to fisheries are marked. In the top-left panel, the UD is so concentrated that it is hidden by receivers. For the particle algorithms, note that our knowledge of the animal’s distribution is shaped by the dataset(s) we consider.

**Fig. 4.**
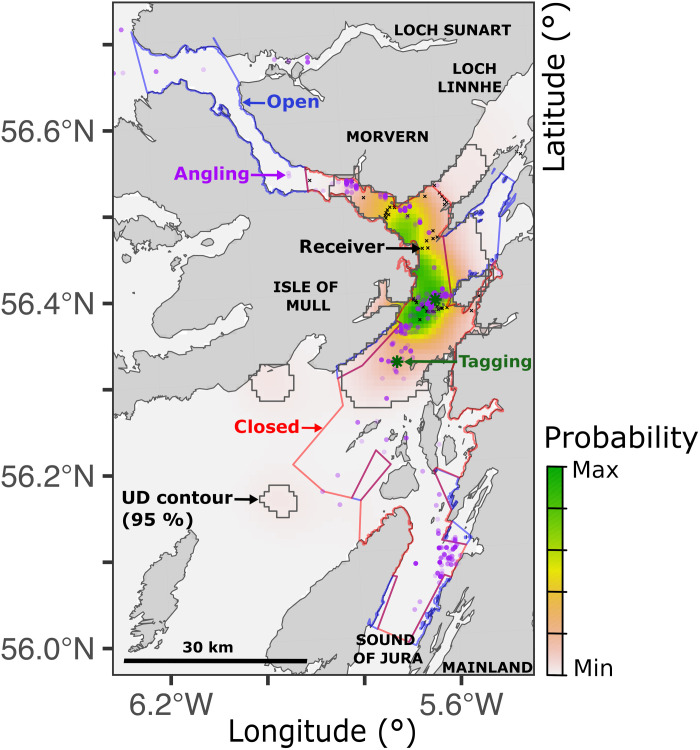
An overall UD for modeled skate, reconstructed by the ACDC algorithm. Colors represent the probability that a modeled individual was located in a given cell at a randomly chosen time (within the analyzed time series). Probabilities are highest in the green zone along western side of the Firth of Lorn. The region containing 95% of the probability mass is delineated by the black contour. Zones open/closed to fisheries, tagging locations (✵), receivers (x), and locations where skate have been captured by anglers (●) are marked.

Estimated UDs (A3.2.1) largely concentrated within the MPA but differed among algorithms (Figs. 3 and 4 and figs. S15 to S19). Of the two heuristic algorithms, the COA algorithm generally produced highly restricted UDs that concentrated around southerly receivers, although maps for two individuals (25 and 28) spanned a larger area (fig. S15). UDs from the RSP algorithm similarly centered in this region, but were more spread out (fig. S16). As for the COA algorithm, RSP UDs were driven by receiver locations. Maps for individuals detected around southerly receivers (e.g., 35) were unaffected by the temporal pattern of detections (figs. S2 and S16).

Particle algorithms indicated more nuanced movement patterns (Fig. 3 and figs. S17 to S19). In the AC algorithm, most UDs spanned the southern receivers (where detections occurred) but were more diffuse and not exclusively centered on receivers (fig. S17). These patterns reflect array design, detection gaps, and individuals’ capacity to move away from receivers. Some UDs suggest movement into receiver gaps in the array’s center, fished zones, and/or further south (beyond MPA boundaries). UDs from the DC algorithm exhibited both similarities and distinct features in the absence of acoustic constraints, the latter including hotspots in shallow to deep water south of the MPA (fig. S18). ACDC-derived UDs integrate features of AC and DC maps. Compared to the AC algorithm, maps were more concentrated within the MPA but also exhibited a redistribution of probability mass beyond MPA boundaries associated with the depth information (fig. S19). For both males and females detected around southerly receivers, the maps suggest notable movements to habitat patches beyond the MPA (especially off the Isle of Mull and north-western Jura). There are also indications of movements further north (including in the deep water off Morvern and beyond the MPA up Loch Linnhe). (For place names, see Figs. 1 and 4.)

Within the MPA, our maps broadly align with skate presence records from angling (A3.2.2), which concentrates in this region (Fig. 4). Potential movements beyond the MPA are poorly represented in angling records.

**Fig. 5.**
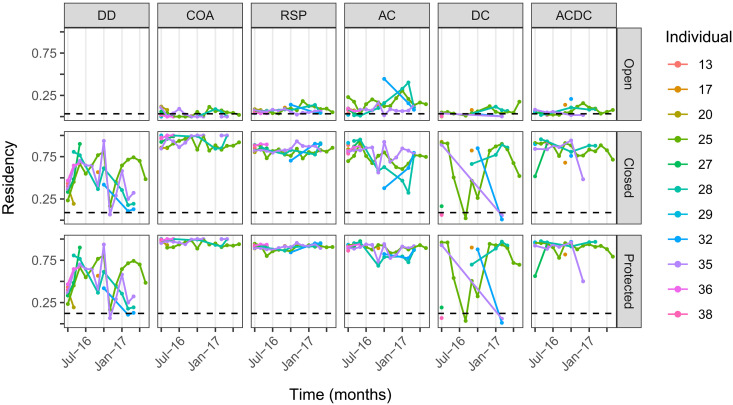
Residency of tagged skate in the MPA. Panels show the proportion of time each individual spent in each management zone (row) in selected months according to each algorithm (column). The terms “Open,” “Closed,” and “Protected” refer to zones within the MPA that are open or closed to fisheries and the entire MPA. Estimates are only shown for months with sufficient data (hence gaps in some time series). Horizontal lines are null model estimates.

In line with UDs, residency estimates (A3.2.3) were high in the MPA but differed among algorithms (Fig. 5). Detection day (DD) proportions varied from 0.07 to 0.94 (median = 0.48). As in simulations (Fig. 2 and fig. S9), heuristic residency estimates were consistently low in open zones (median = 0.05) and high in closed areas (median = 0.88) and the MPA as a whole (median = 0.93). These estimates were relatively insensitive to differences in detection patterns among individuals (fig. S2). Particle algorithm estimates were somewhat higher in open zones and lower in closed zones and the entire MPA. In the AC algorithm, residency ranged between 0.01 and 0.45 (median = 0.10) in open zones, 0.32 and 0.95 (median = 0.78) in closed zones, and 0.68 and 0.97 (median = 0.90) in the entire MPA. In the absence of acoustic constraints, DC algorithm estimates were more variable, although median residency estimates were similar (0.04, 0.80, and 0.88). Median estimates from the ACDC algorithm were also broadly similar, ranging from 0.05 in open zones to 0.88 in closed zones and 0.92 in the entire MPA. Given limited data, seasonal trends in residency are unclear, although it is notable that ACDC residency estimates are broadly elevated in open areas over winter (when commercial fishing is permitted).

#### Sensitivity analyses

In real-world sensitivity analyses (A4), particle algorithm implementation success (A4.1) was affected by algorithm parameterization (fig. S14). Convergence rates were highest in AC algorithm implementations (98 to 100%) and lowest in DC algorithm implementations (67 to 83%). Lower convergence was associated with both restricted and flexible parameterizations.

In the UD analysis (A4.2.1), maps produced by different model parameterizations were broadly similar but differed in detail (fig. S20). As in simulations (figs. S4, S6, and S7), heuristic algorithm UDs were largely unaffected by tuning parameters (fig. S20). In the AC algorithm, restrictive parameterizations generally concentrated maps and flexible parameterizations spread them out (fig. S20). DC algorithm sensitivity was more complex and included contractions, expansions, and shifts in the distribution of hotspots. In the ACDC algorithm, restrictive and flexible parameterizations generally concentrated/expanded patterns of space use, weakening/strengthening the relative importance of habitat patches beyond the MPA, respectively (fig. S20). Where convergence was achieved, the restrictive depth observation model parameterization in particular strengthened patterns of space use within the MPA and dampened the apparent importance of habitats further south.

Residency estimates (A4.2.2) showed a similar degree of sensitivity to algorithm parameterization (fig. S21). Restrictive parameterizations were generally associated with elevated residency estimates, while flexible parameterizations were associated with reduced estimates, especially in the DC algorithm. However, median residency estimates remained remarkably stable. In the ACDC algorithm, median estimates, accounting for all algorithm parameterizations, were 0.05 (in open zones), 0.87 (in closed zones), and 0.93 (in the entire MPA).

## DISCUSSION

This study advances animal tracking for conservation with underwater biotelemetry, building on decades of valuable work in this field. We reveal particle algorithms as a powerful methodology that can strengthen analyses of space use and residency while addressing the limitations of long-established heuristic methods. Our analyses show that particle algorithms achieve substantial improvements in accuracy and precision, while heuristic algorithms can be difficult to tune and insensitive to the temporal pattern of detections and exhibit marked variation in performance. For the Critically Endangered flapper skate, particle algorithms suggested concentrated patterns of space use within an MPA, including time spent beyond receivers in zones that are open to fishing, as well as movements beyond MPA boundaries—extending work on this and related species, with important implications for species conservation (*32*, *38*, *39*). While heuristic methods have been valuable components of the animal-tracking toolbox (*17*, *21*, *22*), particle algorithms provide a probabilistically sound statistical methodology that is sufficiently fast, flexible, and accessible to meet the challenges of many modern animal-tracking datasets. We encourage their application to analyses of species’ movements and MPA efficacy across aquatic ecosystems (*9*, *40*).

We developed a simulation analysis to calibrate heuristic algorithms and evaluate algorithm performance and sensitivity. For heuristic algorithms, previous studies have typically relied on default values (*22*) or subjective judgment (*20*) to set tuning parameters such that variation in algorithm performance and sensitivity is poorly documented (*19*). In our study system, for the COA algorithm, we found that a setting of ΔT=2 days was marginally better than other settings, while for the RSP algorithm there was no optimal choice for the er.ad parameter on average. This insensitivity appears reassuring, but it calls into question the relevance of tuning parameters and their ability to envelop the movement and detection processes that generate observations, upon which the utility of heuristic methods rests (*17*, *19*).

Our simulations revealed a clear ranking of algorithms for estimating patterns of space use and residency. Performance of the two heuristic algorithms was similar but highly variable: simulated patterns were captured well in some instances and poorly in others. In residency analyses, the widely used detection days metric underestimated residency in the MPA, in line with the limited movement capacity of simulated individuals (which could linger within the MPA but beyond receivers). Meanwhile, heuristic algorithms (which restrict positional estimates within receiver arrays, even if individuals move further afield) overestimated residency by a median 12% (SE ≈ 22%). These results are specific to our study system but pertinent for many real-world arrays, where behavior of prevailing analytical methods remains understudied (*17*, *19*).

Compared to heuristic methods, particle algorithms produced more accurate insights into space use and residency. There were clear benefits of data integration, with depth observations refining movements in the AC algorithm and acoustic observations helping to localize movements to relevant regions in the DC algorithm (which otherwise sometimes misplaced individual hotspots). Overall, residency was accurately estimated within a SE of 7.6% (AC), 3.7% (DC), and 2.8% (ACDC). This quantification of “array precision” should become an effective means to evaluate and improve monitoring programs in future. Here, simulations suggest that data integration enabled up to threefold improvements in estimates of space use and residency (lower mean error, higher precision). We anticipate considerable opportunities to extend this kind of analysis in other systems to evaluate (i) existing arrays, (ii) alternative array designs, and (iii) the number of receivers required to estimate metrics of interest with a specified degree of accuracy.

The above simulation results were broadly robust to algorithm parameterization. Heuristic algorithms were highly insensitive to algorithm parameterization, but even particle algorithms were generally robust to mis-specification of individual parameters (such as the maximum movement speed), given the presence of other constraints (such as detection range) (*19*). The DC algorithm showed the greatest sensitivity, as expected given the absence of acoustic constraints and the heterogenous distribution of depth habitats in our study system. In other study systems, we recommend that studies leveraging multiple datasets conduct similar analyses to examine algorithm sensitivity (*41*).

Our real-world analyses of flapper skate showcase enhancements in the use of acoustic telemetry for mobile species conservation. Within the MPA we study, we found that heuristic methods produced limited maps of space use that were driven by receiver positions (and insensitive to the temporal pattern of detections), while particle analyses indicated specific habitat patches in between receivers that appear to be important, filling gaps in angling records and detection analyses (*32*). The drivers of movement within these regions are unclear, but depth, temperature, light, sediment, and/or prey preferences may play a role, as shown for other elasmobranchs (*42*, *43*). A wide range of studies have linked detection patterns to habitat selection (*44*, *45*) and this is an important next step in our research. By probabilistically representing individual movements, particle algorithms have clear potential to strengthen habitat-selection analyses and support the identification of sites, within and beyond MPAs, that may benefit from conservation measures (*46*, *47*).

Within the MPA, our algorithms also suggested movements beyond receivers into fished zones. Given the sensitivity of elasmobranchs to trawling (*4*, *29*), this result is potentially concerning and suggests that further analyses of skate-fisheries interactions in our study area are warranted. Further afield, we recognize that many MPAs contain fished zones where receiver deployments are limited by fishing activity (*6*). The capacity to investigate movements into these zones is an important development for quantifying the trade-offs between species conservation and extractive activities. This should inform marine spatial planning in many regions (*48*).

By integrating tracking datasets with previous knowledge of species movement, we were also able to estimate individual residency at spatial scales relevant to management, notwithstanding limited receiver coverage. For analyzed skate, our algorithms indicate a remarkably high degree of residency in the MPA. Earlier analysis of residency indices indicated that skate can spend weeks at a time around receivers (*32*), but robust estimates of residency for flapper skate (and other mobile species) in regions with incomplete receiver coverage have remained elusive. Here, we found the commonly used detection days metric (*17*) variable and poorly indicative of residency. Meanwhile, heuristic algorithms indicated consistently high MPA-scale residency across individuals, irrespective of detection gaps. Properly accounting for movements during detection gaps is a step forward in studies of the efficacy of MPAs for mobile species. For flapper skate, our best (ACDC) median residency estimate is 92%, suggesting that the MPA is sufficiently large to protect analyzed skate for much of the year. This result is robust to algorithm parameterization. This work strengthens the evidence that MPAs can be effective conservation measures for mobile species and supports the use of permanent, rather than seasonal, fisheries closures for flapper skate and other species that display similar movement patterns (*9*, *38*, *39*). However, fisheries closures should be accompanied by wider fisheries management measures to protect individuals that exhibit weaker site affinity (*31*).

We also documented evidence for movements into specific habitat patches beyond MPA boundaries that may warrant protection. For flapper skate, these include an area of moderately deep (>100 m) water off Jura and a shallow (50 m) sandy-muddy habitat off the Isle of Mull that is bounded by a deeper (100 m) trench (punctuated by subsurface rocks). It is tantalizing to notice characteristics of an egg nursery in this location (*37*). Currently, the only known flapper skate nursery is in the Red Rocks and Longay MPA (further north). While sample size is limited, we found no strong evidence for movements toward Red Rocks, so the search is on to determine where flapper skate using the MPA lay eggs (*37*). The identification of essential fish habitats is crucial for mobile species conservation and a key objective of many telemetry studies, including our own work (*40*, *49*). The unique capacity of our algorithms to facilitate this research, via the identification of specific habitats beyond receivers that are used by tagged animals, is a useful step forward. This should support the establishment of connected MPA networks for mobile species (*40*).

Improving the movement and observation models we use to model underwater biotelemetry data is an important task for future research (*18*, *24*). Our skate movement model would be informed by additional (e.g., accelerometer) data on individual behavior and swimming speeds. Multi-sensor tags hold considerable promise for this in future and should be leveraged by other studies (*11*). Similarly, our acoustic observation model would be improved with expanded detection range testing. While challenging, range-testing data are often collected and should be incorporated in analyses (*50*). However, in our study system, improvements to the depth observation model are the priority: In sensitivity analyses, different model parameterizations affected the localization of hotspots within the MPA and the relative importance of habitat patches further afield. The key knowledge gap is the extent to which flapper skate exhibit benthic versus pelagic behavior. In rugged bathymetric landscapes, fish behavior affects the locational information provided by depth observations and the ease with which particle filters converge. Here, we attribute convergence failures to potential inadequacies in the depth observation model and the difficulty of finding valid routes through a bathymetric maze. In complex environments, particle algorithms may struggle and discretization of the state space for filtering (*51*) or tempered Hamiltonian Monte Carlo (*52*) may be required. For further commentary, see (*53*). For flapper skate and other aquatic species, continued tagging and monitoring, coupled with further analytical development in these directions, will support improved analyses in years to come.

This work has implications for the spatial management of mobile species. By integrating animal movement modeling and tracking data, we reveal how particle algorithms can represent movements beyond receivers, refine maps of space use, and improve residency estimates. These developments enhance the value of biotelemetry data for mobile species conservation, informing MPA placement, size, and management (*9*). Our case-study analyses of flapper skate provide a concrete example: confirming the value of fisheries restrictions in an existing MPA, indicating patterns of habitat use, quantifying movements into fished zones, and highlighting specific habitats beyond the MPA that may warrant protection. These insights into space use, residency in regions of interest, and unprotected habitats beyond receivers represent great steps forward for aquatic conservation (*45*). That being said, model precision remains dependent on data quality and algorithm-informed study design may be beneficial moving forward. This work should support the conservation of flapper skate and other threatened species across the globe (*10*, *40*).

Particle algorithms have potential applications beyond spatial management. Acoustic telemetry and biologging are expanding rapidly (*15*, *16*). With the growth of international telemetry networks, the spatial scale over which animals can be tracked is also widening. Analyses of telemetry data require statistical models that accurately reconstruct movements and represent uncertainty. Our integrative modeling framework is thus well placed to strengthen research across the biotelemetry sphere, from ecological analyses of individual movements (*45*) to the conservation objectives addressed in this paper (*40*). We foresee opportunities to refine habitat-selection analyses (*47*), investigate co-occurrence patterns (*54*), and support animal-borne oceanography (*55*), fisheries management (*56*), climate change mitigation (*57*), habitat restoration (*58*), and impact assessments (*59*). The methods are relevant for research projects across disparate systems (*15*, *16*) and have the potential to support progress toward strategic objectives, such as Sustainable Development Goal 14 (*40*). We hope that this study encourages advances toward this goal in the second half of the United Nations Decade of Ocean Science for Sustainable Development.

## MATERIALS AND METHODS

### Study system

We selected a 14,000 km^2^ case-study system in Scotland (Fig. 1). The bathymetry encompasses shallow-water platforms, deep basins, and channels up to 350 m in depth. Within the site, the Loch Sunart to the Sound of Jura MPA spans 741 km^2^ and a depth range of 0 to 290 m. Current management prohibits commercial fishing except in eight seasonally (October to March) fished zones. For further description, including bathymetric data sources, see text S1 and table S1.

From 2016 to 2017, a passive acoustic telemetry system comprising 58 Vemco receivers was established in part of the MPA (*32*). Skate were captured in the same area and tagged with acoustic transmitters and archival (depth) tags (*35*). For full details, including array and tag properties, see text S2.

### Workflow

We conducted simulation-based and real-world analyses of animal movements (see table S2 for an overview). In each analysis, we analyzed movements using two heuristic algorithms (COAs and RSPs) and our particle algorithms (Table 1). The workflow comprised three stages: (i) coordinate estimation, (ii) mapping, and (iii) residency estimation, which are explained in general terms below before the details of our analyses. Algorithms were implemented in R, v.4.3.1 (*60*), using the patter (*28*) and RSP (*22*) packages. All code is available online (*61*).

#### Coordinate estimation

*COAs*The first algorithm we used to estimate coordinates for mapping was the COA algorithm. This estimates coordinates as weighted averages of receiver locations where detections were recorded over sequential time intervals (duration: ΔT ). We selected three ΔT values (an optimal value, a restrictive value, and a more flexible value) to examine algorithm performance and sensitivity (see the “Simulation-based analysis” section).

*RSPs*The RSP algorithm interpolates coordinates along the shortest paths between receivers. We used the default settings for tuning parameters except er.ad, which tunes coordinate weights for mapping. As above, we selected three values by simulation (see the “Simulation-based analysis” section). For implementation details, see text S3.

*Particle algorithms*We also implemented the AC, DC, and ACDC particle filtering–smoothing algorithms (*19*). To implement these algorithms, we formulated a Bayesian SSM for the location (strictly “state”) $s_{t}$ of an individual through time ( t∈{1,2,…,T} ), given the (acoustic and archival) observations ( $y_{1:T}$ ), that is, $f\left(s_{1:T}|\;y_{1:T}\right)$ . Our SSM represents an underlying behavioral switching movement process that includes a “low activity” state (encompassing resting) and a more active state. Movements are connected to acoustic observations (detections, nondetections) by a Bernoulli observation model in which detection probability declines logistically with distance from receivers. The depth observation process is described by a truncated Gaussian distribution centered on the seabed (capturing skate’s benthic lifestyle) with a variance accounting for observational and bathymetric uncertainty. For the model formulation, see text S4.1 (mathematics), table S3 (notation summary), and fig. S1 (visualization). We considered a “best-guess” parameterization as well as a restrictive and flexible parameterization for the movement and observation submodels. SSM parameterizations were based on previous research, domain knowledge, and a literature review. See texts S4.2 and S4.3 (for details) and table S4 (for a summary of parameter values).

Particle filtering and smoothing algorithms perform inference for the SSM, targeting the marginal distribution $f\left(s_{t}\;|\;y_{1:T}\right)$ . The distribution of individual locations is approximated by a Monte Carlo simulation of N weighted particles, which represent candidate positions of the individual. In the particle filter, a movement model simulates particle movement from one time step to the next; observation models weight particles in line with their compatibility with the data; and a resampling step duplicates or eliminates particles accordingly. The AC, DC, and ACDC algorithms differ only in the data incorporated during this process (acoustic, depth, or both datasets). The number of particles is a trade-off between speed and convergence: Sufficient particles are required to ensure that at least some particles are compatible with the data at every time step. After subsequent particle smoothing, the result is a set of particles that approximate $f\left(s_{t}\;|\;y_{1:T}\right)$ . For implementation details, see text S4.4.

#### Mapping

For COAs and particle algorithms, we generated maps of space use, i.e., UDs, by kernel smoothing estimated coordinates (*28*). For the RSP algorithm, a dynamic Brownian-bridge movement model is used to smooth coordinates (*22*). For implementation details, see text S5.

#### Residency

Alongside UDs, we estimated the proportion of time spent in selected areas (i.e., residency). We considered residency in our study system in three areas: zones (i) open and (ii) closed to fisheries and (iii) the entire MPA. Residency was estimated as the proportion of (i) UD volume (for heuristic algorithms) or (ii) resampled particles (for particle algorithms) in each area. We also considered (iii) the proportion of DD in each area (a standard metric) for comparison.

### Simulation-based analysis

Our first analysis was a simulation-based analysis (see table S2 for the overview). In this analysis, we simulated individual movements and corresponding observations in our study system and reconstructed patterns of space use using COAs, RSPs, and particle algorithms. We used simulation results to calibrate heuristic algorithms for real-world analyses, evaluate algorithm performance and sensitivity, and validate the use of particle algorithms for later analyses (see the next section). Full details are available in text S6.

In outline, we simulated movements from “tagging locations” within the study area for 100 hypothetical flapper skate over a 1-month period, according to our best-guess skate movement model (see text S6.1). For each movement path, we simulated acoustic and depth observations, following best-guess models for the aforementioned acoustic and depth observation processes. For each path, we generated a true UD via kernel smoothing. Residency was calculated as the proportion of path steps in each area. Using the simulated datasets, we assessed the performance and sensitivity of algorithms applied to simulated observations in terms of how well they recovered path UDs and residency (see texts S6.2 and S6.3). In the following description, we label performance and sensitivity analyses as “A1” and “A2,” respectively. Analytical steps are labeled in the same way (as A1.1, A1.2, etc.), following table S2.

In performance analyses, we evaluated the skill with which algorithms reconstructed simulated patterns of space use and residency (A1). This analysis included a null model (uniform UD, excluding land) alongside heuristic and particle algorithms parameterized with optimal or best-guess parameters (A1.1). For heuristic algorithms, a simulation approach was used to select optimal parameter values: For each path, we computed the ME between the UD for each simulated path and reconstructed UDs for a range of candidate parameter values and identified the parameter value with the lowest ME on average (A1.1.1). Particle algorithms were parameterized according to the data-generating processes (A1.1.2). Heuristic algorithms were implemented for each acoustic time series, and particle algorithms (AC, DC, and ACDC) were implemented for each (i) acoustic, (ii) depth, and (iii) combined dataset, respectively (A1.2). We then evaluated algorithm performance (A1.3) by visually comparing simulated and reconstructed UDs for three selected paths (A1.3.1), the distribution of ME between all simulated and reconstructed UDs (A1.3.2), and the distribution of residency error (estimated residency minus true residency) between simulated and reconstructed patterns, by algorithm (A1.3.3). Residency error was quantified for the null model and the detection days metric (as benchmarks), as well as the heuristic and particle algorithms.

In sensitivity analyses, we used a subset of paths (1 to 3) to explore algorithm sensitivity (A2). In this analysis, we re-implemented the algorithms with restrictive and flexible parameterizations (A2.1). For the particle algorithms (which are stochastic), we ran each algorithm implementation three times to examine reproducibility. We then analyzed algorithm sensitivity and reproducibility (A2.2) by visualizing patterns of space use (A2.2.1), ME (A2.2.2), and residency error (A2.2.3) for each algorithm parameterization/implementation.

For our study area, the simulation analyses (i) confirmed that particle algorithms outperform heuristics, (ii) revealed algorithm sensitivity, and (iii) demonstrated that stochastic particle runs produce consistent results. By visually comparing maps between AC, DC, and ACDC algorithms, we also gauged the relative importance of acoustic and/or depth datasets in refining maps of space use.

### Real-world analyses

In real-world analyses (A3 and A4), we analyzed movement patterns of tagged skate (fig. S2 and table S5). Data were sourced from a study of 42 individuals tagged with acoustic and archival tags (33 of which were detected) (*32*). We selected 11 individuals (30%) with sufficient data for analysis. (For data processing, see text S7.1.) For each individual, we analyzed movements over each month using (i) the acoustic data, (ii) the depth data, and (iii) the combined data. We analyzed 48 individual/month time series in total (1 to 14 months per individual).

Following the simulation workflow, for each individual, for each month, we analyzed data using heuristic algorithms (acoustic datasets only) and the particle algorithms (acoustic, depth, and combined datasets). In our main analyses (A3), each algorithm was implemented once using optimal (heuristic) or best-guess (particle algorithm) parameters (A3.1). We estimated UDs for each dataset (A3.2.1). We assessed similarities and differences among individuals and algorithms visually, given limited data. UDs were aggregated (A3.2.2) to map the overall pattern of space use and assess overlap with long-term (1975–2024) skate presence angling records (*62*). Residency was estimated as in simulations (A3.2.3). In sensitivity analyses (A4), we re-implemented algorithms with restricted and flexible parameterizations (A4.1) and analyzed changes in UDs (A4.2.1) and residency (A4.2.2). For the overview, see table S2. For full details, see text S7.2.

## Supplementary Materials

### This PDF file includes:

Supplementary Text Figs. S1 to S21 Tables S1 to S5 References
