# Supplementary material for: Animal tracking with particle algorithms informs protected area design
Source: Sci Adv. 2025 Nov 26;11(48):eadx0255. doi: 10.1126/sciadv.adx0255 (PMC12652237; doi:10.1126/sciadv.adx0255)
Supplement: Supplementary file 1 — Supplementary Text Figs. S1 to S21 Tables S1 to S5 References [file sciadv.adx0255_sm.pdf]

Science Advances
AAAS

Supplementary Materials for

Animal tracking with particle algorithms informs protected area design

Edward Lavender *et al.*

Corresponding author: Edward Lavender, edward.lavender@eawag.ch; Sophie Smout, scs10@st-andrews.ac.uk

Sci. Adv. **11**, eadx0255 (2025)
DOI: 10.1126/sciadv.adx0255

This PDF file includes:

Supplementary Text
Figs. S1 to S21
Tables S1 to S5
References

Supplementary text

1. Study site

1.1. Domain

Our study system forms a 14,000 km^2 area on the west coast of Scotland. The study system is centred in the Firth of Lorn and was chosen to envelope the Loch Sunart to the Sound of Jura Marine Protected Area (MPA) and surrounding regions, while remaining sufficiently small in size for analyses.

1.2. Bathymetry

1.2.1. Data sources

We required a bathymetric raster to reconstruct patterns of space use for tagged flapper skate within the study area. A raster for the study system was built using data from three sources:

- **A Firth-of Lorn dataset** (5 x 5 m), compiled from an Order 1a hydrographic survey, obtained from Howe. et al. (*63*).
- **Supplementary datasets** for selected regions within the study area, obtained from Admiralty (https://seabed.admiralty.co.uk/).
- **A Scotland-wide dataset** (6 arc-second resolution), obtained from Digimap © Crown copyright and database rights [2019] Ordnance Survey (100025252).

For full details on bathymetric data sources, see Table S1. Coastline data (for UD estimation and/or mapping) were obtained from Digimap (Westminster constituencies) and GADM (wider context) (*64*).

1.2.2. Dataset assembly

Study site datasets. We assembled two datasets for the study system: a high-resolution dataset (named 'bathy-high') and a lower resolution dataset (named 'bathy-low'). Datasets were assembled as follows:

- **Merger.** The Firth of Lorn and supplementary datasets were merged onto a 5 x 5 m grid (556,740,000 cells) with a Universal Transverse Mercator (Zone 29N) Coordinate Reference System.
- **Resampling.** All supplementary datasets were resampled for the merger using bilinear interpolation.
- **Smoothing.** Deep spikes in the merged dataset, which reflect errors in bathymetric soundings (and are particularly prevalent along coastlines), were processed. Spikes were flagged in cells where the bathymetric depth was more than 50 m deeper than the median depth of the surrounding eight cells and cut back to the median depth. Visual assessment of this heuristic suggested it was effective.
- **Aggregation.** For selected analyses, bathymetric data were aggregated onto a 500-by-500-pixel grid (a resolution of approximately 200 x 278 m) using the arithmetic mean (see §5–7). All receivers were in valid locations (i.e., in water) on the aggregated bathymetric dataset (bathy-low).

Scottish dataset. We processed the Scotland-wide dataset for RSP analyses (see §5).

- **Cropping.** Data were cropped to the area spanned by our 'bathy' grids, expanded by 200 km in the four cardinal directions.
- **Resampling.** Data were resampled to match the resolution of bathy-low.
- **Merger.** Within the domain of the bathy-low dataset, we replaced the values on the Scottish dataset with those from bathy-low to ensure consistency between analyses.

1.2.3. Dataset uncertainty

We required estimates of bathymetric uncertainty to reconstruct patterns of space use for tagged skate with the particle algorithms (see §4.2.3). Uncertainty in bathymetric datasets depends on survey accuracy and subsequent processing:

- **Order 1a survey.** The Howe. et al. (*63*) survey was conducted to an industry standard, 95 % confidence vertical-uncertainty limit of $\sqrt{(0.500 + \left(0.013\ b(\boldsymbol{s})\right)^2}$, where $b(\boldsymbol{s})$ is the bathymetric depth in the location (s_x, s_y) defined by $\boldsymbol{s}$ (*65*). This ranges from 0.71 m to 3.85 m at 0 m and 290 m (the maximum depth in the domain of the dataset), respectively.
- **Order 2 surveys.** The accuracy of the supplementary datasets is uncertain. However, for Order 2 surveys, the bathymetric uncertainty at 350 m depth may be up to ± 8.08 m (*65*).

Based on these statistics, we considered a bathymetric uncertainty of ± 10 m for the bathy-high dataset, given the 95 % confidence threshold, the merger of multiple datasets and spike processing. For the bathy-low dataset, there is an additional aggregation uncertainty that we represented (see §4.2.3).

1.3. Flow regime

Information on the flow regime was required to parameterise our particle algorithms (see §4.2.1):

- **Tidal range.** Within the study area, tidal range can be up to ± 3 m (based on tidal charts).
- **Storm surges.** Tidal ranges may be compounded by ± 2 m storm surges.
- **Flow regime.** The flow region is complex, driven by the interaction between the tides and bathymetry. In the West Scotland Coastal Ocean Modelling System (*66*, *67*), in our study system, average bottom currents are 0.09 ms^{-1} but can exceed 4 ms^{-1}. In the Gulf of Corryvreckan tidal channel (which is 3.5 km in length), current speeds reaching 4.75 ms^{-1} have been recorded (*68*).

2. Passive acoustic telemetry

2.1. Array design

From 2016–17, a passive acoustic telemetry system was established in the study site to monitor skate (*32*). In total, 58 Vemco VR2, VR2W and VR2AR 69 kHz receivers were deployed (Fig. 1). Array configuration was driven by various research projects and logistics. Array coverage fluctuated in line with changes in receiver number and placement, with aggregated 50 % detection-probability regions spanning approximately 4–14 km^2 (*32*).

2.2. Acoustic and archival tags

Flapper skate were captured by angling in and around the array and given acoustic and/or archival tags.

Acoustic transmitters. The acoustic tags (Vemco V13s and Thelma Biotel MP-13s) were programmed with random transmissions every 30–90 s (over an 18-month battery life). Tag power output was 147 dB and 153 dB re 1 uPa at 1 m for the V13 and MP-13 tags, respectively (*32*).

Archival tags. The archival tags (Star Oddi Milli-TDs) were programmed to record depth (± 4.77 m) every two minutes, until their removal from recaptured skate (*35*).

2.3. Detection probability

We required estimates on detection probability to parameterise our particle algorithms (see §4.2.2). Drift tests of our array are described by ref. (*69*) and summarised by ref. (*32*). The protocol was as follows:

- **Set up.** Tests were conducted (with limited resources) for a subset of receivers on 19th–20th October 2016.
- **Transmitter deployment.** For each test, two acoustic transmitters were deployed on a line from a boat (approximately 5 m below the surface and 2 m above the seafloor, respectively).
- **Drift.** With the boat's engines and depth sounders turned off, the boat was allowed to drift away from each receiver. Locations and transmissions were recorded by a Vemco VR100 Digital Tracking Receiver.
- **Modelling.** Detection-probability relationships were explored by modelling acoustic observations (detections, non-detections), inferred from receiver records versus known transmissions, as a function of the distance between receivers and transmitters (and other variables) (*69*).

Drift test analyses demonstrated that detection probability declines from 0.97 near to receivers to 0.5 by 400–450 m (*69*). The maximum observed detection range was 708 m. However, in the nearby Loch Etive study system, a detection range of 767 m for V13 tags was recently measured. Considering limited testing, the maximum detection range in our system likely exceeds these thresholds (see §4.2.2).

3. Heuristic algorithms

We reconstructed patterns of space use in simulation (§6) and real-world (§7) analyses using two heuristic algorithms as well as our particle algorithms:

- **COA algorithm.** The COA algorithm was implemented with the `coa()` function in the `patter` package (*28*, *70*).
- **RSP algorithm.** The RSP algorithm was implemented with the `runRSP()` function in the `RSP` package (*22*). This routine interpolates 'relocations' along the shortest paths between receivers for UD estimation. Shortest paths are computed using a transition layer that defines horizontal, in-water distances between receivers, which we built from our aggregated bathymetry dataset (bathy-low).

The algorithms depend on the following parameters:

- **ΔT.** The COA algorithm depends on the time interval over which detections are averaged (ΔT). In our analyses, we used an 'optimal' value, as well as a more restrictive and flexible setting, selected by simulation (see §6.2). Multiple values were used to examine sensitivity (see §6.3).
- **`er.ad`.** The RSP algorithm depends on multiple tuning parameters. We selected three values for the `er.ad` parameter, which tunes the rate at which uncertainty around interpolated 'relocations' grows with distance from a receiver (see §6.2). The detection range (γ) was held constant at 3000 m, which is our best-guess for the study system (see §4.2.2). Default settings were used for the other parameters.

Coordinates estimated by COA and RSP algorithms were translated into UDs (see §5 for generic details). In selected analyses, UDs were used to estimate the proportion of time spent in selected areas (i.e., residency) from the proportion of their volume in those areas. The analyses in this paper are outlined in Table S2. Implementation details are provided in sections §6–7.

4. Particle algorithms

4.1. State-space model

Alongside heuristic algorithms, we reconstructed patterns of space use using particle algorithms (*19*). Unlike heuristic methods, particle algorithms are based on a formal statistical (state-space) model of individual movements for which inference is performed. In this section, we formalise a discrete-time model of skate movements in our study system in general terms, following ref. (*19*). See Fig. S1 for a visualisation and Table S3 for a summary of notation. In subsequent sections, we explain model parameterisation (§4.2–3) and inference using particle algorithms (see §4.4).

4.1.1. Posterior

The model represents the joint distribution $f(\boldsymbol{s}_{1:T} \mid \boldsymbol{y}_{1:T})$ of a skate's state ($\boldsymbol{s}$) given the data ($\boldsymbol{y}$) through time ($t \in \{1, 2, \ldots, T\}$). The individual's state is denoted $\boldsymbol{s}_t = (s_{x,t},\ s_{y,t},\ \phi_t,\ \text{behaviour}_t)$ and comprises its two-dimensional location (s_x, s_y) on (or near) the seabed, its heading (ϕ_t) and behaviour.

We consider two behavioural states: a 'low activity' state (including resting) and an 'active' state (see §4.1.2). Behavioural states were defined *a priori*: we examined individual depth (vertical activity) time series and distinguished 'low activity' and 'active' behavioural states by a vertical activity threshold of 0.25 m, following ref. (*36*) (see §6.1 and §7.2.2). We recognise that we ought to infer behaviour alongside the other state variables jointly in a Bayesian framework; however, this is numerically expensive. Our approach here might lead to a slight underestimation of uncertainty, but is cheaper.

The joint distribution is proportional to a prior $f(\boldsymbol{s}_{1:T})$ (the movement process) and a likelihood $f(\boldsymbol{y}_{1:T} \mid \boldsymbol{s}_{1:T})$ (the observation process):

$$f(\boldsymbol{s}_{1:T} \mid \boldsymbol{y}_{1:T}) \propto f(\boldsymbol{s}_{1:T})\, f(\boldsymbol{y}_{1:T} \mid \boldsymbol{s}_{1:T}). \qquad \text{eqn 1}$$

We consider three versions of this model, with the same prior, but different observation processes for (a) acoustic data, (b) archival data and (c) the combined dataset. The models (and corresponding inference algorithms) are labelled AC, DC and ACDC respectively (*19*).

4.1.2. Prior

We represent $f(\boldsymbol{s}_{1:T})$ as a discrete-time Markovian process:

$$f(\boldsymbol{s}_{1:T}) = f(\boldsymbol{s}_{t=1}) \prod_{t=2}^{T} f(\boldsymbol{s}_t \mid \boldsymbol{s}_{t-1}). \quad \text{eqn 2}$$

Initialisation. The term $f(\boldsymbol{s}_{t=1})$ is the probability density function of the individual's initial state. We treat the initial location as known (see §6.2), or uniform within some subset of the study area (see §7.2.1) compatible with the initial observations (A) and assume a uniformly distributed initial heading, i.e.,

$$f(\boldsymbol{s}_{t=1}) = \text{Uniform}(s_x, s_y; A) \cdot \text{Uniform}(\phi; -\pi, \pi). \quad \text{eqn 3}$$

The state $\boldsymbol{s}_{t=1} = (s_{x,1},\ s_{y,1},\ \phi_1,\ \text{behaviour}_1)$ includes behaviour. However, as behaviour states were assigned *a priori,* we drop this dimension from eqn 3 for clarity.

Correlated random walk. The term $f(\boldsymbol{s}_t \mid \boldsymbol{s}_{t-1})$ is the probability density of moving from $\boldsymbol{s}_{t-1} \rightarrow \boldsymbol{s}_t$. We model movement as a two-dimensional, behavioural switching correlated random walk, based on step lengths and turning angles i.e.,

$$\boldsymbol{s}_t \mid \boldsymbol{s}_{t-1} = \big(s_{x,t-1} + d_t \cos(\phi_{t-1} + \Delta\phi_t),\ s_{y,t-1} + d_t \sin(\phi_{t-1} + \Delta\phi_t),\ \phi_{t-1} + \Delta\phi_t,\ \text{behaviour}_t\big), \quad \text{eqn 4}$$

where d is the step length, ϕ is the heading and $\Delta\phi_t$ is the change in heading (turning angle). During inference, this sampling procedure is implemented iteratively until a valid location (in water) is found (see eqn 8 and §4.4).

The movement density is represented by $f(\boldsymbol{s}_t \mid \boldsymbol{s}_{t-1})$. The density of the current step length and heading ($f_{d,\phi}$) is given by the densities of the step length (f_d) and turning angle ($f_{\Delta\phi}$):

$$f_{d,\phi}(d_t, \phi_t \mid \phi_{t-1}) = f_d(d_t)\ f_{\Delta\phi}(\phi_{t-1} - \phi_t). \quad \text{eqn 5}$$

The step length and turning angle define the change in location in a polar coordinate system, which is mapped to the change in x and y coordinates (s_x, s_y) by:

$$\boldsymbol{g}\left((\Delta s_x, \Delta s_y)\right) = (d_t, \phi_t) = \left(\sqrt{\Delta {s_x}^2 + \Delta {s_y}^2}\ , \arctan(\Delta s_y\ /\ \Delta s_x)\right), \quad \text{eqn 6}$$

where $\Delta s_x = s_{x,t-1} - s_{x,t}$, $\Delta s_y = s_{y,t-1} - s_{y,t}$ and $\arctan(\Delta s_y\ /\ \Delta s_x)$ returns the angle (in radians) in the interval $[-\pi, \pi]$. The arctan function, or `atan` in our `Julia` code, corresponds to the standard definition of an `atan2` function in computer programming languages. This is a variation of the arctangent function that returns the angle between $-\pi$ and π, as opposed to the usual atan function that returns the angle between 0 and π (or $-\pi/2$ and $\pi/2$). For further details, see the Julia documentation (*71*). By change of variables, the density of the movement to $\boldsymbol{s}_t$ is thus:

$$f(\boldsymbol{s}_t \mid \boldsymbol{s}_{t-1}) = f_{d,\phi}\left(\boldsymbol{g}\left((\Delta s_x, \Delta s_y)\right) \,\middle|\, \phi_{t-1}\right) \left|\det\left(J\left((\Delta s_x, \Delta s_y)\right)\right)\right| \quad \text{eqn 7}$$

where the absolute determinant of the Jacobian J is derived from $\boldsymbol{g}$ as $1/\sqrt{\Delta {s_x}^2 + \Delta {s_y}^2}$. Incorporating the criterion that the individual can only swim in water completes the movement model:

$$f(\boldsymbol{s}_t \mid \boldsymbol{s}_{t-1}, \text{in water}) = \frac{1}{z(\boldsymbol{s}_{t-1})} f(\boldsymbol{s}_t \mid \boldsymbol{s}_{t-1})\, I(\boldsymbol{s}_t \text{ is in water}), \qquad \text{eqn 8}$$

where I is the indicator function and z is a normalisation constant. In practice, this constant is obtained during inference by Monte Carlo simulation (see ref. (*19*) and §4.4).

Step lengths. We model $f_d(d)$ using two Cauchy distributions for 'low activity' (e.g., resting) and 'active' behavioural states, truncated between zero and mobility:

$$f_d(d) = \begin{cases} \text{Truncated Cauchy}(d;\ k_1, \theta_1, 0, \text{mobility}) \text{ if behaviour}_{t-1} = 0 \text{ ('low activity')} \\ \text{Truncated Cauchy}(d;\ k_2, \theta_2, 0, \text{mobility}) \text{ if behaviour}_{t-1} = 1 \text{ ('active')}. \end{cases} \qquad \text{eqn 9}$$

The location (k) and scale (θ) parameters determine the shape of the distribution and the mobility is the maximum moveable distance between $t-1$ and t (i.e., the truncation threshold). Smaller values for k_1 and θ_1, compared to k_2 and θ_2, produce a low-activity step-length distribution that is more restrictive than that for active behaviour. As noted above, behavioural states were defined *a priori* from depth (vertical activity) time series. The behaviour at time $t-1$ was assigned based on vertical activity between $t-1$ and t. During inference (§4.4), at each $t-1$, we simulated movement into new location(s), given the behaviour over that timeframe (at $t-1$).

Turning angles. Turning angles were modelled using a Gaussian distribution:

$$f_{\Delta\phi}(\Delta\phi) = \text{Gaussian}(0.0, \sigma^2_{\Delta\phi}). \qquad \text{eqn 10}$$

Smaller $\sigma_{\Delta\phi}$ settings in this model produce smaller turning angles (i.e., more correlated random walks).

4.1.3. Likelihood

The term $f(\boldsymbol{y}_{1:T} \mid \boldsymbol{s}_{1:T}) = \prod_{t=1}^{T} f(\boldsymbol{y}_t \mid \boldsymbol{s}_t)$ in eqn 1 is the joint likelihood; that is the probability of the observations given the latent locations. Our observations include acoustic detections (1) and non-detections (0) at each operational receiver k (denoted $y_{k,t}^{(A)} \in \{0,\ 1\}$) and depth observations (denoted $y_t^{(D)}$). In the three versions of our model for the acoustic, depth and combined data types, $f(\boldsymbol{y}_t \mid \boldsymbol{s}_t)$ equals $f\left(\boldsymbol{y}_t^{(A)} \,\middle|\, \boldsymbol{s}_t\right)$, $f\left(y_t^{(D)} \,\middle|\, \boldsymbol{s}_t\right)$ and $f\left(\boldsymbol{y}_t^{(A)} \,\middle|\, \boldsymbol{s}_t\right) f\left(y_t^{(D)} \,\middle|\, \boldsymbol{s}_t\right)$ respectively.

Acoustic observations. The likelihood of acoustic observations was modelled as a Bernoulli probability mass function:

$$f\left(\boldsymbol{y}_t^{(A)} \,\middle|\, \boldsymbol{s}_t\right) = \prod_k \text{Bernoulli}\left(y_{k,t}^{(A)}; p_{k,t}(\boldsymbol{s}_t)\right), \qquad \text{eqn 11}$$

where detection probability is denoted p. We modelled p as a truncated logistic function of the Euclidean distance $h(\boldsymbol{s}_t, \boldsymbol{r}_k)$ between the transmitter and the receiver ($\boldsymbol{r}_k = (s_{x;k}, s_{y;k})$):

$$p_{k,t}(\boldsymbol{s}_t) = \begin{cases} \left(1 + e^{-(\alpha + \beta \times h(\boldsymbol{s}_t, \boldsymbol{r}_k))}\right)^{-1} \text{ if } h(\boldsymbol{s}_t, \boldsymbol{r}_k) < \gamma \\ 0 \text{ otherwise} \end{cases}, \qquad \text{eqn 12}$$

where α and β are parameters and γ is the detection range.

Depth observations. The likelihood of the depth observation is modelled using a Gaussian distribution with a mean (μ_{depth}) on the seabed $\left(b(\boldsymbol{s}_t)\right)$ and a standard deviation σ_{depth}, truncated between the surface ($\text{lim}_{\text{shallow}} = 0$ m) and the maximum depth ($\text{lim}_{\text{deep}} = 350$ m); i.e.,

$$f\left(y_t^{(D)} \mid \boldsymbol{s}_t\right) = \text{Truncated Gaussian}\left(y_t^{(D)}; \mu_{\text{depth}}, \sigma_{\text{depth}}^2, \text{lim}_{\text{shallow}}, \text{lim}_{\text{deep}}\right). \quad \text{eqn 13}$$

This model effectively elevates the probability of $y_t^{(D)}$ in latent locations where $y_t^{(D)}$ is close to the seabed, in line with skate's benthic lifestyle. The σ_{depth} parameter accounts for observational and bathymetric uncertainty and tunes the probability density of pelagic movements off the seabed.

4.2. Model parameterisation

We formulated and parameterised our state-space model using available information for flapper skate and other elasmobranchs. In the following sections, the formulation and parameterisation of the movement model (§4.2.1) and acoustic (§4.2.2) and depth (§4.2.3) observation models are explained. For a summary of all model parameterisations, see Table S4.

After leveraging domain knowledge to parameterise our model, we performed inference of the latent locations (see §4.4). While it is possible to infer locations and static parameters simultaneously, the information available in passive acoustic telemetry systems is limited (*19*). Data driven estimation of static parameters is also computationally expensive, so we did not pursue this option here.

4.2.1. Movement model

The movement model (prior) represents how far skate can swim in a given time step. This model was based on a review of the literature on locomotory capacity (especially swimming speeds) in other elasmobranchs and limited available data from flapper skate.

4.2.1.1. Literature review

Elasmobranchs. In the literature, studies of elasmobranch locomotion are relatively scarce and focus on sharks (*72*). Routine swimming speeds are particularly poorly documented, but in sharks are generally thought to be around one body length per second (BLs^{-1}) (*72–74*). In shortfin mako (*Isurus oxyrinchus*), which is one of the fastest shark species, an accelerometry study documented mean sustained cruising speeds of 0.4 BLs^{-1} or 0.9 ms^{-1}, comparable to those of other regionally endothermic fish (0.7–1.5 ms^{-1}) (*75*). The maximum burst swimming speed was 2.5 BLs^{-1} (5.0 ms^{-1}), though speeds exceeding 4.9 BLs^{-1} (9.8 ms^{-1}) have been documented during 'escape' responses and speeds up to 10 BLs^{-1} (20 ms^{-1}) are thought possible (*76*).

Batoids. In batoids, locomotion is based on oscillatory ('flapping') or undulatory ('rippling') movements of the enlarged pectoral fins (*72*, *77*). Pelagic rays swim principally by oscillatory locomotion—a type of lift-based propulsion that is efficient for high-speed cruising. For oceanic manta rays (*Manta birostris*), median speeds observed from satellite tracking of six medium-sized individuals (disc length [DL] ≈ disc width ≈ 3.5–4.5 m) were approximately 0.1 DLs^{-1} (0.3 ms^{-1}) (*78*). Higher speeds of 0.5–1.0 DLs^{-1} (2.78–4.17 ms^{-1} or 10–15 kph) have been documented during breeding (*79*); maximum burst speeds are likely to be higher still (*80*). A more recent study reported speeds ranging between approximately 0.29 DLs^{-1} (during resting) to 0.75 DLs^{-1} during feeding (*81*). Other studies on cownose rays (*Rhinoptera bonasus*) have reported comparable statistics, with migration speeds between 2.06–2.57 ms^{-1} (*82*). An experimental study observed speeds of 0.9–2.1 BLs^{-1} in undisturbed conditions, with faster speeds observed in response to back strikes from waves (*83*). (We calculated this range based on a reported mean body length over the study of 28.7 cm and swim speeds of 24.8–59.2 ms^{-}

^{1}.) Turning velocity estimates in oscillatory and undulatory rays generally range between 0.2–1.1 ms^{-1} and 0.3–0.8 ms^{-1} respectively (*84*), but can be faster (*80*).

Benthic batoids. In benthic batoids (including the majority of stingrays and skates), undulatory locomotion is more common and swimming efficiency is generally considered to be lower (*82*). Flow tank experiments have been conducted for blue-spot stingray (*Taeniura Lymma*, DL ≈ 0.16 m) and juvenile freshwater stingray (*Potamotrygon orbignyi,* DL ≈ 0.13 m) at steady swimming speeds of 0.9–3.0 DLs^{-1} (0.22–0.55 ms^{-1}) (*85*) and 2.5 DLs (0.33 ms^{-1}) (*86*), respectively. In wave experiments (*83*), reported swimming speeds in undisturbed conditions for Atlantic stingray (*Dasyatis sabina*) and South American freshwater stingray (*P. motoro*) span a similar range (approximately 0.5–2.5 BLs^{-1}).

Skate. For skate (Rajidae), data are limited. Skate are often sedentary and small species, such as little skate (*Leucoraja erinacea*, DL ≈ 7 cm) may 'punt' over the seafloor (*87*). Observations from trawl fisheries show that skate (and sharks) are often unresponsive to trawling or swim at slower speeds (< 1 ms^{-1}) (*88*). For little skate (which is between an undulator and oscillator), optimal cruising speeds are 1 DLs^{-1} (0.07 ms^{-1}), although speeds of 1.25 DLs^{-1} can be sustained for a few minutes and speeds of up to 2 DLs^{-1} are documented over shorter time intervals (*89*). The extent to which these results scale to other species is unclear, but it is generally thought that in larger species size-relative routine speeds are lower (though absolute speeds may be higher). Trawl footage of species such as barndoor skate (*Dipturus laevis*) show that individuals may swim for a few seconds in front of a trawl, implying movement speeds up to 1.5 ms^{-1} (3 knots) or approximately 1 DLs^{-1}, before trying to escape below the gear (*90*). However, swimming capacity depends on context-specific conditions, including substrate hydrodynamic forces and temperature (*72*).

Flapper skate. We used the literature on elasmobranch locomotion to bound our models of flapper skate movement in Scotland. In our dataset, captured skate vary in disc width from 0.75–1.75 m (which we take as an approximate measure of DL). For much of the time, flapper skate are probably largely sedentary on the seabed, resting or waiting to ambush benthic prey (*35*). Putative cruising speeds up to 1 DLs (0.75–1.75 ms^{-1}) are broadly comparable to pelagic rays and suggests speeds of 90–210 m per 2 min (the resolution of depth measurements). Speeds up to 2 DLs^{-1} (1.5–3.5 ms^{-1} or 180–420 m per 2 min) seem plausible. Over a few seconds, maximum burst speeds may exceed these limits but are likely less than the 3 DLs^{-1} required to reach the speeds (2.25–5.25 ms^{-1}) achieved by big pelagic rays and endothermic sharks. However, substantially faster ground speeds may occur in areas of strong current flow. While average current speeds in our study system are negligible, speeds up to 4.75 ms^{-1}, capable of carrying skate 570 m per 2 min, have been documented (see §1.3).

Datasets. Little data exists with which to corroborate suggested flapper skate swimming speeds. Previous analyses of movements between receivers suggested potential speeds of up to 273 m per 2 min (2.27 ms^{-1}), which for larger skate is 1.30 DLs^{-1}, but likely underestimate attainable speeds. Ascent rates of up to 100 m per two minutes, and vertical movements up to 150 m per 2 min, are known (*35*, *36*). Based on these results, in a previous, small-scale, illustrative analysis, we formulated a behavioural switching movement model in which movement probabilities during periods of high activity declined rapidly beyond 250 m up to a maximum mobility of 500 m (2.38 DLs^{-1} for a 1.75 m skate) (*18*). However, for the present study, this is likely overly restrictive.

4.2.1.2. Parameter settings

The aforementioned literature, our earlier analyses and exploratory trials of the convergence properties of different model formulations, led to the formulation of a correlated random-walk movement model for flapper skate (eqns 4–10, Fig. S1). The model was parameterised as follows.

Step lengths: best-guess parameterisation. In our best-guess parameterisation, step lengths were modelled with the following parameters during 'low-activity' or 'active' behaviour:

$$\begin{cases}(k_1, \theta_1, \text{mobility}) = (0.0, 5.0, 1095.0) \\ (k_2, \theta_2, \text{mobility}) = (5.0, 100.0, 1095.0).\end{cases} \quad \text{eqn 14}$$

This parameterisation exhibits the following characteristics:

- **Low-activity distribution ($\boldsymbol{k_1, \theta_1}$, mobility).** During 'low-activity' (including presumed resting periods), the model makes steps of zero length most likely, steps up to 25 m relatively plausible and longer steps unlikely.
- **Active distribution ($\boldsymbol{k_2, \theta_2}$, mobility).** In the 'active' step-length distribution, longer steps are also less likely, especially beyond 1 DLs^{-1} (210 m per 2 min for a large skate).
- **Truncation by mobility.** Both 'resting' and 'active' step-lengths distributions were constrained by the mobility parameter of 1,095 m per 2 min (the maximum moveable distance in 2 min). This value is based on (a) an absolute maximum swimming speed of 525 m per 2 min (2.5 DLs^{-1} for a 1.75 m skate or 4.37 ms^{-1}) in (b) an area of high current flow (4.75 ms^{-1}). This model formulation makes slower movements more likely, while permitting faster movements with lower probability.

To examine the sensitivity of our analysis to our parameter choices, we also formulated a more restrictive model and a more flexible model.

Step lengths: restricted parameterisation. In the restrictive parameterisation, parameter settings were as follows:

$$\begin{cases}(k_1, \theta_1, \text{mobility}) = (0.0, 5.0, 985.5) \\ (k_2, \theta_2, \text{mobility}) = (2.5, 50.0, 985.5),\end{cases} \quad \text{eqn 15}$$

which represents a 50 % shrinkage in the location and scale parameters during active behaviour and a 10 % shrinkage in mobility (Fig. S1).

Step lengths: flexible parameterisation. In the flexible parameterisation, parameter settings were corresponding inflated:

$$\begin{cases}(k_1, \theta_1, \text{mobility}) = (0.0, 5.0, 1204.5) \\ (k_2, \theta_2, \text{mobility}) = (7.5, 150.0, 1204.5),\end{cases} \quad \text{eqn 16}$$

with a 50 % increase in the location and scale parameters during active behaviour and a 10 % increase in mobility (Fig. S1).

Turning angles. All movement model parameterisations used a Gaussian distribution to represent turning angle, with a standard deviation of $\sigma_{\Delta\phi} = 1.5$. This corresponds to a weak–moderate correlation ($\rho \approx 0.32$) in heading. This choice was based on experience in our study area that weakly correlated random walks have improved convergence properties during inference. Due to computational restrictions, it was not possible to trial more restrictive/flexible turning angle distributions in our sensitivity analyses (see §6–7). However, we expect our

results are robust to limited changes in correlation strength. Moderate to strong correlations ($\sigma_\phi \leq 1.2$) induced convergence failures in initial experiments.

4.2.2. Acoustic observation model

Model formulation. The acoustic observation model was based on a truncated, distance-decaying, logistic detection-probability function (Fig. S1). Detection probability was set to decline with distance from a receiver at a rate determined by two coefficients (α, β), truncated by the maximum detection range (γ) (*18*, *19*). This model formulation is based on evidence that the distance between a receiver and transmitter is the main driver of detection probability and can be parameterised using standard range-testing protocols, such as drift tests (*50*). We recognise that other variables, such as noise, affect detection probability, but their effects are less generalisable and difficult to parameterise with available datasets (*32*).

Parameterisation information. We parameterised our detection-probability model using drift-test analyses (see §2.3), plus literature and manufacturer specifications:

- **Drift tests.** Drift tests demonstrated that probability declines to 0.5 by 400–450 m and is low beyond 700 m (see §2.3).
- **Literature.** While comparable data from other study systems are limited, detection ranges of 500–850 m for V13 tags and VR2W receivers are not uncommon in the literature (*91*, *92*) and align with manufacture guidelines. In deep-water marine ecosystems, ranges up to at least 1,628 m have been reported (*93*) and in other systems tests of similar technologies have reported ranges exceeding 9–10 km (*94*, *95*).

Best-guess parameterisation. In our best-guess parameterisation, we set:

$$(\alpha, \beta, \gamma) = (4.0, -0.0094, 3000.0). \quad \text{eqn 17}$$

This parameterisation exhibits the following features:

- **Half-detection probability.** The model approximately recapitulates drift-test results, exhibiting half detection probability at 425 m and low detection probability beyond 700 m.
- **Detection range.** The detection range (3000 m) is approximately twice the maximum range reported in other deep water systems (*93*) but considerably lower than maximum known ranges for similar technology (*94*, *95*). In our algorithms, the detection range sets a hard threshold beyond which detections are assumed impossible. The value of 3000 m is compromise that permits detections, with low probability, at moderate, but not excessive, distances from receivers.

Restrictive parameterisation. In our restrictive parameterisation, we set:

$$(\alpha, \beta, \gamma) = (3.0, -0.01175, 2250.0), \quad \text{eqn 18}$$

which represents a 25 % steepening of the function (25 % decrease in α, 25 % increase in β and 25 % decrease in γ).

Flexible parameterisation. In our flexible parameterisation, we set:

$$(\alpha, \beta, \gamma) = (5.0, -0.00705, 3750.0), \quad \text{eqn 19}$$

which represents a 25 % shallowing of the function.

4.2.3. Depth observation model

Initial model formulation. We formulated a Gaussian depth observation model (eqn 13, Fig. S1). During inference, this model updates particle weights in line with the correspondence between the observed depth $\left(y_t^{(D)}\right)$ and the bathymetric depth $(b(\boldsymbol{s}))$. The mean of the Gaussian distribution (μ_{depth}) was set to the depth of the seabed, in line with the benthic lifestyle of skate (*34*, *96*, *97*). Initially, the standard deviation ($\sigma_{\text{depth}} = 20$) was set accounting for accounting for uncertainty in the bathymetric depth (± 10 m, see §1.2.3), tidal elevation (± 3 m, see §1.3), storm surges and waves (± 2 m, see §1.3) and manufacturer-quoted tag accuracy (± 4.77 m, see §2.2) but later revised (see below).

Revised model formulation. In initial simulation experiments, we found that inference with the above model was extremely difficult: in a complex bathymetric landscape, the model opens up a labyrinth of possible routes that particles have to explore but ultimately renders few compatible with the data. This makes convergence in the particle filter extremely hard to achieve—a challenge that can be compounded by mismatches between the underlying data-generating process and the modelled process. This is a known difficulty in particle filtering contexts and a hard problem to solve. In this context, our solution was to aggregate the bathymetry data onto a 500-by-500-pixel grid (as described in §1.2.2) and propagate additional uncertainty in bathymetry depth in the depth-observation model. This makes it much easier for particles to move into cells that are compatible with the depth observations (since cells are larger and encompass a larger range of depths), facilitating convergence (at the cost of reduced precision) in the particle filter.

Best-guess parameterisation. In our best-guess parameterisation, we set:

$$\left(\mu_{\text{depth}}, \sigma_{\text{depth}}, \text{lim}_{\text{shallow}}, \text{lim}_{\text{deep}}\right) = (b(\boldsymbol{s}), 100.0, 0.0, 350.0). \qquad \text{eqn 20}$$

This parameterisation exhibits the following features:

- **Mean.** The model upweights locations for which the bathymetric depth is close to the observed depth.
- **Standard deviation.** The standard deviation accounts for known sources of uncertainty (± 20 m) and the approximate range (± 70 m) of bathymetric values in each cell, while permitting potential pelagic movements (*98*, *99*), and effectively 'dampens' the influence of depth observations sufficiently to achieve convergence in most instances (see Main Text Results).

Restrictive parameterisation. In the restrictive parameterisation, we trialled:

$$\left(\mu_{\text{depth}}, \sigma_{\text{depth}}, \text{lim}_{\text{shallow}}, \text{lim}_{\text{deep}}\right) = (b(\boldsymbol{s}), 50.0, 0.0, 350.0), \qquad \text{eqn 21}$$

which represents a 50 % reduction in the standard deviation (i.e., reduced uncertainty, pelagic behaviour and/or dampening).

Flexible parameterisation. In the flexible parameterisation, we set:

$$\left(\mu_{\text{depth}}, \sigma_{\text{depth}}, \text{lim}_{\text{shallow}}, \text{lim}_{\text{deep}}\right) = (b(\boldsymbol{s}), 150.0, 0.0, 350.0), \qquad \text{eqn 22}$$

which represents a 50 % increase in the standard deviation (i.e., increased uncertainty, pelagic behaviour and dampening).

4.3. Uncertainty

We recognise that our observation models are imperfect and uncertain. We therefore examine algorithm sensitivity to variation in parameter values (within reasonable limits). By performing inference using (i) acoustic data, (ii) depth data and (iii) both datasets, we also reveal the extent

to which independent datasets suggest similar patterns of space use and the extent to which patterns based only on acoustic or depth data are refined by the integration of both datasets.

4.4. Inference

The particle algorithms perform inference for our state-space model (§4.1), given static parameters are specified (see §4.2). We performed inference considering (i) acoustic observations, (ii) depth observations and (iii) both datasets. For convenience, we label these procedures as the acoustic-container (AC), depth-contour (DC) and acoustic-container depth-contour (ACDC) algorithms, respectively. All algorithms comprise a filtering and a smoothing step; the only difference lies in the data incorporated during filtering. Algorithms were implemented using the `patter` package, v.1.0.1 (*28*, *70*):

- **Filter.** The particle filter was implemented with the `pf_filter()` function. We used 100,000–150,000 particles (see §6–7). The movement process was implemented iteratively until a valid movement (on the bathy-low raster) was found or for a maximum number of 10,000 trials. Particles which failed to make a valid move in 10,000 trials were killed. Low-variance resampling was implemented every time step. The filter stores a subset of equally weighted particles at each time step. The equal weighting is achieved by resampling. The computational complexity of this routine is $O(TN)$, where T is the number of time steps and N is the number of particles (*100*).
- **Smoother.** Particle smoothing was performed using the two-filter smoother implemented by `pf_smoother_two_filter()`. We used 1,500–2,000 particles for smoothing (see §6–7). This algorithm re-weights particles from a forward filter run and a backward filter run in line with the probability density of movements between pairs of locations (*100*). Computational complexity is $O(TN^2)$ for this routine. One hundred Monte Carlo simulations were used to compute the normalisation constant for the density of the movement model (see eqn 8). As for the filter, a subset of equally weighted particles are stored following smoothing at each time step. Note that with insufficient smoothing particles the two filters may not align at every time step (that is, there may be no valid moves between the subset of retained particles on the backward filter and those from the forward filter). In this instance, `patter` randomly retains 50 % of the particles from the forward filter and 50 % from the backward filter. In this study, we only analysed outputs for which proper smoothing was possible on >90 % of time steps.

Following smoothing, UDs were estimated from particle samples as described in §5. In selected analyses, the expected time spent in selected areas (i.e., residency) was estimated from the proportion of (smoothed) particles in those areas.

5. Mapping

5.1. Kernel smoothing

For simulated paths (see §6.1) and coordinates estimated by the COA and particle algorithms (see §6–7), we generated maps of space use using kernel smoothing (*28*). For mapping, coordinates were summarised on our 500-by-500-pixel grid (bathy-low) in terms of probability-of-use (P), which is the probability that an individual is located in a particular cell at a randomly chosen time; i.e.,

$$P_I \propto \sum_{i,t} \delta_{I,I_{i,t}} w_{i,t}, \qquad \text{eqn 23}$$

where I indexes grid cells, i indexes coordinates, δ is the Kronecker delta and w denotes weights (*28*). (To avoid confusion, as above we re-iterate that stored particles are equally weighted.)

For the COA algorithm, P_I amounts to the proportion of COAs in cell I, since only one COA is estimated per time interval and equal weights are assumed. For the particle algorithms, P_I accounts for the number of copies of each particle within a cell (within and across time steps).

Smooth maps of space use were generated by kernel smoothing the summarised (weighted) coordinates, with edge correction. For edge correction, we used an observation window defined by the Westminster constituencies shapefile from Digimap (see §1.2.1). The shapefile was used (rather than our bathy-low pixel image) for computational efficiency. The window was simplified via the `simplify.owin()` function in the `spatstat.geom` package, using a tolerance (`dmin`) of 1,000 m (*101*). This heuristic maintained an accurate representation of the coastline while substantially speeding up UD estimation (from approximately 161 → 10 s). For speed, kernel bandwidth was defined from the combined variance of summarised coordinates using the ad-hoc method (*102*). Kernel smoothing was implemented via the `patter map_dens()` function, which wraps `density.ppp()` from `spatstat.explore` (*101*).

5.2. Brownian bridges

For the RSP algorithm, a dynamic Brownian-bridge movement model is used to smooth coordinates (*22*). One map is estimated for each set of detections spaced less than one day apart. We implemented the method, as defined in the `RSP` package, using an enlarged bathymetric raster to mitigate estimation errors (see §1.2.1). For each dataset, the overall map was defined as the re-normalised aggregation of all maps for that dataset (resampled onto our 500-by-500-pixel [bathy-low] grid).

6. Simulation analyses

6.1. Data simulation

We performed simulation analyses to investigate algorithm behaviour (performance and sensitivity) and inform the implementation and interpretation of real-world analyses (see Table S2 for the overview and §7 for details).

The simulation analysis was based on 100 hypothetical movement paths (and corresponding observational datasets), simulated over a one-month period ($T = 21{,}600$ two-minute time steps). The two-minute time resolution was chosen to align with the time resolution of real-world analyses, which incorporated depth observations measured every two minutes (see §2.2 and §7). Paths were simulated as follows.

Initialisation. Each path was initialised randomly in a location $(s_{x,t=1}, s_{y,t=1})$ within the receiver array (A), with a random heading (ϕ_t):

$$\begin{aligned}(s_{x,t=1}, s_{y,t=1}) &\sim \text{Uniform}(s_x, s_y; A) \\ \phi_{t=1} &\sim \text{Uniform}(-\pi, \pi).\end{aligned} \quad \text{eqn 24}$$

Behavioural states. Subsequently, movements were simulated in discrete-time following our best-guess behavioural switching correlated random walk model (see §4.2.1). Low-activity and

active behavioural states were simulated based on observed properties of real depth time series. Following ref. (*36*), we distinguished 'low activity' (0) and 'active' (1) behaviour in depth time series based on a vertical activity threshold of 0.25 m per 2 min. We observed that individuals spent approximately 50 % of time in each behavioural state and, in our simulations, sampled an initial behaviour from a discrete uniform distribution accordingly:

$$\text{behaviour}_{i=1} \sim \text{Uniform}(\{0,1\}). \qquad \text{eqn 25}$$

Subsequently, for each behaviour_i, we iteratively simulated blocks (indexed by i) of low-activity or active behaviour that were maintained for k_i time steps. The duration for which behaviours were maintained was based on simple Gamma (shape, rate) models derived from the observed time series:

$$k_i \sim \begin{cases} \text{round}(\text{Gamma}(0.730, 0.163)) \text{ if behaviour}_i = 0 \\ \text{round}(\text{Gamma}(0.960, 0.168)) \text{ if behaviour}_i = 1. \end{cases} \qquad \text{eqn 26}$$

Each behaviour_i thus lasts for k_i time steps (with different behaviour_i generally lasting for different durations). After each block of low-activity or active behaviour, a behavioural transition occurred:

$$\text{behaviour}_{i+1} = 1 - \text{behaviour}_i \qquad \text{eqn 27}$$

Blocks of behaviours were simulated iteratively for the duration of the timeline, that is, while $\sum_{i=1} k_i \leq T$. These blocks were then unwrapped into a time series of behavioural states, such that each time step t was associated with a single behavioural state. The time series of behavioural states were used in the next stage of the simulation.

Movements. Following initialisation, for each subsequent time step, movements were simulated by sampling from our best-guess flapper skate model (eqns 4–10). Step lengths were modelled using truncated Cauchy distributions (with location [k_1 and k_2], scale [θ_1 and θ_2] and truncation parameters [0.0, mobility]):

$$f_d(d_t) = \begin{cases} \text{Truncated Cauchy}(d_t;\ 0.0, 5.0, 0.0, 1095.0) \quad \text{if behaviour}_{t-1} = 0 \\ \text{Truncated Cauchy}(d_t; 5.0, 100.0, 0.0, 1095.0) \text{ if behaviour}_{t-1} = 1. \end{cases} \qquad \text{eqn 28}$$

Turning angles were modelled using the Gaussian distribution:

$$f_{\Delta\phi}(\Delta\phi_t) = \text{Gaussian}(\Delta\phi_t;\ 0.0, 1.5). \qquad \text{eqn 29}$$

Patterns of space use for the simulated path were generated by kernel smoothing (see §5).

Observations. For each movement path, we collected state vectors $\boldsymbol{s}_t = (s_{x,t}, s_{y,t}, \phi_t, \text{behaviour}_t)$ and simulated acoustic and depth observations, following the aforementioned data-generating processes (eqns 11–13 in §4.2) with our best-guess parameters (see eqns 17 and 20 in §4.2). That is, acoustic observations were sampled from a Bernoulli model:

$$\boldsymbol{y}_t^{(A)} \sim f\left(\boldsymbol{y}_t^{(A)} \mid \boldsymbol{s}_t\right) \qquad \text{eqn 30}$$

$$f\left(\boldsymbol{y}_t^{(A)} \mid \boldsymbol{s}_t\right) = \prod_k \text{Bernoulli}\left(y_{k,t}^{(A)}; p_{k,t}(\boldsymbol{s}_t)\right)$$

$$p_{k,t}(\boldsymbol{s}_t) = \begin{cases} \left(1 + e^{-(4.0 - 0.0094 \times h(s_t, r_k))}\right)^{-1} \text{ if } h(\boldsymbol{s}_t, \boldsymbol{r}_k) < 3000 \text{ m} \\ 0 \text{ otherwise,} \end{cases}$$

where $\boldsymbol{r}_k$ denotes the location for receiver $k \in \{1, 2, \dots, 58\}$. In simulations, we used real receiver locations but imagined that all receivers were active in the one-month period over which data were simulated. (In reality, only a subset of receivers was deployed in any one month and we accounted for this in real-world analyses.)

Depth observations were sampled from a Gaussian model with a mean $\mu_{\text{depth}} = b(\boldsymbol{s}_t)$ and a standard deviation $\sigma_{\text{depth}} = 100.0$, truncated between 0 and 350 m:

$$y_t^{(D)} \sim f\left(y_t^{(D)} \mid \boldsymbol{s}_t\right)$$
$$f\left(y_t^{(D)} \mid \boldsymbol{s}_t\right) = \text{Truncated Gaussian}\left(y_t^{(D)}; b(\boldsymbol{s}_t), 100.0, 0.0, 350.0\right). \quad \text{eqn 31}$$

6.2. Performance analyses (A1)

In performance analyses, we evaluated the skill with which algorithms reconstructed simulated patterns of space use and residency. See Table S2 for the overview.

A1.1. Algorithm parameterisation. Algorithms were parameterised with optimal or best-guess parameters.

A1.1.1. Heuristic algorithms. For heuristic algorithms, a simulation approach was used to select optimal parameter values for ΔT (COA) and `er.ad` (RSP) (see Main Text and Fig. S4).

A1.1.2. Particle algorithms. Particle algorithms were parameterised according to the data-generating processes (eqns 28–31).

A1.2. Algorithm implementation. Heuristic algorithms were implemented for each acoustic time series and particle algorithms (AC, DC and ACDC) were implemented for each (a) acoustic, (b) depth and (c) combined dataset, respectively.

We initialised particle algorithms at the simulated capture or recapture location (on the forward and backward filter runs, respectively).

We used a 'capture container' to shrink the domain within which particles were resampled towards the simulated 'finishing' (capture or recapture) location. The capture container was implemented as an additional 'observation model'. At each time step, we computed the probability of each particle given the distance of the particle from the 'finishing' location. Particles must be within $\text{radius} = \text{mobility} + \text{mobility} \times \Delta T$ of the 'finishing' location (where mobility is the maximum moveable distance in one time step and ΔT is the number of time steps until the capture/recapture event). We used the extra mobility buffer to relax the requirement for particles to finish exactly in the correct location, instead permitting particles anywhere within mobility m of the correct location (that was also compatible with the simulated data).

Acoustic containers were used for AC and ACDC algorithm runs to facilitate convergence (*19*). Acoustic containers work similarly to capture containers but kill particles that are incompatible with the location(s) of the next acoustic detection(s), given the detection range, rather than particles which are incompatible with a known 'finishing' location (*19*).

In the particle filter, we used 100,000 particles. For smoothing, 1500 particles were used (see §4.4). We expected 1,500 particles to be sufficient to approximate the two-dimensional distribution $f(\boldsymbol{s}_t \mid \boldsymbol{y}_{1:T})$. In practice, we achieved relatively low effective sample sizes (ESS) with 1,500 particles. (For 'performance' analyses, the mean ESS across all simulations was 195 particles.) We also observed one situation in which the forward and

backward filters were insufficiently aligned for adequate smoothing (see §4.4). For this reason, we boosted the number of particles in real-world analyses (see §7).

Algorithms were implemented on a 2019 AMD EPYC 7742 server using 10 threads. Median total computation time was 1.1, 1.8 and 2.0 hours for the DC, AC and ACDC algorithms, respectively. UDs were reconstructed as described in §5. For each algorithm, we checked the proportion of simulations for which UDs were successfully estimated.

A1.3. Algorithm performance. We evaluated algorithm performance by the accuracy with which patterns of space use and residency were estimated.

A1.3.1.*Maps of space use: visual evaluation.* We visually evaluated algorithm performance for the first three paths by comparing simulated and reconstructed UDs.

A1.3.2.*Maps of space use: quantitative evaluation.* To examine algorithm performance in terms of patterns of space use for all simulated paths, we computed and visualised the distribution of mean absolute error between simulated UDs, the UD for a null model (a flat probability distribution over the marine area defined by bathy-low) and UDs from the heuristic and particle algorithms. We used the mean absolute error as a metric of model skill as a previous study showed this metric effectively distinguishes between the performance of our algorithms (*19*).

A1.3.3.*Residency*. We also examined algorithm performance in terms of the accuracy with which residency (within zones open or closed to fisheries and the MPA as a whole) was estimated. Residency for the simulated path was defined as the proportion of time steps spent in each zone. Residency for the null model amounts to the proportional area of each zone in the study (marine) domain. This was compared against a standard residency index (detection days) as well as residency estimated from the heuristic and particle algorithms. 'Detection days' were defined as the proportion of days with detections at receivers in closed zones (where all receivers were located) or the wider MPA. Residency for heuristic and particle algorithms was estimated as explained previously (see §3 and §4.4). We then evaluated algorithm performance in terms of the distribution of residency error (the difference between the estimated and true residency), across all paths, by algorithm/zone.

6.3. Sensitivity analyses (A2)

In sensitivity analyses, we selected a subset of simulated datasets (1–3) for which to compare alternative algorithm parameterisations and thus evaluate algorithm sensitivity. See Table S2 for the overview.

A2.1. Algorithm implementation. We re-implemented all algorithms with 'restrictive' and 'flexible' settings. For COAs and RSPs, we restricted and inflated the $\Delta T \in \{2\text{ days}, 1\text{ day}, 3\text{ days}\}$ and $\text{er.ad} \in \{500, 250, 750\}$ parameters, on the basis of the aforementioned simulation analysis of heuristic parameters (A1.1.1). For particle algorithms, we trialled restrictive and flexible parameterisations of the movement model (see §4.2.1.2), acoustic observation model (see §4.2.2) and/or depth observation model (see §4.2.3), while holding other parameters constant (at data-generating settings). Each best-guess, restrictive and flexible parametrisation of the particle algorithms was implemented three times to examine algorithm reproducibility (given inference via particle algorithms is

based on a stochastic sequential Monte Carlo procedure). Other settings, such as the number of particles, were fixed as in performance analyses (A1). Convergence for each algorithm parameterisation was tracked, as explained above (A1.2).

A2.2. Algorithm sensitivity and reproductivity.

A2.2.1. ***Maps of space use: visual examination.*** As in performance analyses (A1), we visualised UDs for the three selected paths for each algorithm parameterisation. For the particle algorithms, we also visualised UDs for a selected path (1) for the three independent runs of each algorithm parameterisation to examine reproducibility of maps of space use.

A2.2.2. ***Maps of space use: quantitative evaluation.*** We computed and visualised mean absolute error between UDs for each simulated path and the reconstructed UD from each algorithm parameterisation. As in performance analyses (A1), this analysis included UDs from the null model as well as UDs from the heuristic and particle algorithms.

A2.2.3. ***Residency***. We computed and visualised residency error for each path and algorithm parameterisation, as in performance analyses (A1).

7. Real-world analyses

7.1. Datasets

In our real-world analysis, we analysed patterns of space use from tagged flapper skate, using acoustic data (from 33 individuals) and archival data (from 21 individuals) made available by previous studies (Fig. S2).

We considered three datasets:

- The acoustic data
- The archival data
- The combined data

Datasets were processed as follows:

- **Blocking.** Each dataset was split into individual/month datasets.
- **Individuals.** We focused on individuals/months with both sets of observations (to facilitate comparisons between AC, DC and ACDC algorithm outputs).
- **Inclusion criteria.** Datasets were retained only for individuals that were at liberty for the entire month (ensuring comparability among maps of space use) and for individuals that were detected in at least two different weeks on a total of seven days in a given month. This criterion excluded individuals with the sparsest time series (i.e., individuals that were detected following tagging and then appeared to leave the array).

7.2. Analyses (A3–4)

For each individual/month dataset, we reconstructed patterns of space use and estimated residency using the COA, RSP and particle algorithms (see Table S2 for the overview). For each dataset, we implemented algorithms with optimal or best-guess parameters (A3) alongside restrictive and flexible algorithm parameterisations to quantify sensitivity (A4). The COA and

RSP algorithms were implemented exactly as in simulation analyses (see §6). Particle algorithm implementations were adjusted to account for the features of the real-world data (see §7.2.1–2).

7.2.1. Particle algorithm initialisation

Unlike simulation analyses, particle algorithms could not be initialised at known capture/recapture locations (since individuals were not captured/recaptured exactly at the start/end of each individual–month dataset).

Initially, we trialled initialising the filter in locations sampled uniformly from the part of the study area compatible with the data (the default option in `patter`). However, this approach performed poorly: more precise initial locations were required to achieve convergence with moderate numbers of particles. (The issue in our case is that mobility is large, relative to the size of the study area, and the container within which particles are sampled quickly becomes large as the time to the most recent detection(s) increases. Most particles are thus initialised in unlikely locations and are killed, leading to poor convergence properties.)

To improve initialisation in the particle filter, for each dataset, we used a simulation approach:

- **Geolocation records.** We identified the nearest known geolocations of that individual (to the start and end of each time series), based on the full detection time series and the Skatespotter mark-recapture angling database (https://skatespotter.sams.ac.uk) in which all known capture events (individual, date, location) are recorded. (This approach enables geolocation records beyond the one-month timeline directly accessible to the filter to refine filter initialisation.) The database generally only records capture dates, but we identified the exact time of capture events by visual examination of the raw depth time series: following tagging, individuals rapidly descend (by >100 m) to the seabed; recapture events are thus visible as 'spikes' in the depth time series, as individuals are pulled to the surface and then released; and archival tag recovery events are similarly visible from rapid ascents (by > 100 m) as skate are pulled towards the surface (*36*). The exact coordinates of capture events are not always recorded exactly, but the vast majority of (recorded) angling in our study area occurs at known angling marks, which made it possible to assign capture locations with sufficient accuracy for this analysis.
- **Movement simulation.** From each geolocation record, we simulated 30 realisations of our best-guess movement model over the duration between that record and the 'start' of the filter timeline ($t = 1$ or $t = T$ for the forward and backward filters, respectively). The simulation of movement trajectories followed the workflow described previously (see §6.1).
- **Initialisation container.** At the start of the filter timeline, we assumed the individual was located in a 'container' around the geolocation record with a radius equal to (a) two thirds of the maximum detection range (2000 m) or (b) the maximum distance of simulated positions from the geolocation record (whichever was larger). For gaps exceeding 12 hours in duration, we added an additional 5000 m buffer (in acknowledgement of greater uncertainty in individual movements over longer periods).
- **Initialisation samples.** We further assumed that at the start of the filter timeline, individuals must be in a location on the high-resolution bathymetric grid (bathy-high) within 20 m of the observed depth, accounting for accounting for uncertainty in the bathymetric depth (± 10 m, see §1.2.3), tidal elevation (± 3 m, see §1.3), storm surges (± 2 m, see §1.3) and tag accuracy (± 4.77 m, see §2.2). We sampled initial locations

uniformly, with replacement, from the set of possible locations on bathy-high within the initialisation container. This process was repeated for the start and end of each time series (for the forward and backward filter runs, respectively).

7.2.2. Particle algorithm implementation

Particle algorithms were implemented for the acoustic (AC), depth (DC) and combined (ACDC) datasets. Each algorithm run for a given individual/month was initialised with the same (best-guess) starting locations, since precise initialisation was important to achieve convergence. Behavioural states were assigned *a priori* to time steps, from the depth (vertical activity) time series, using the threshold 0.25 m (see also §6.1). Similar to simulation analyses (§6.2), all particle filters included an additional 'capture container' observation model structure to facilitate consistency between starting and finishing locations in the forward and backward filter runs. We used this structure to shrink the 'container' within which particles were located at each time step towards the centroid of the set of possible 'ending' locations. For example, at the end of the forward filter, we assumed the individual must be within

$$\text{radius} = \text{mobility} + \max_j \left(\left\| \left(s_x^{(c)}, s_y^{(c)} \right) - \left(s_{x_{t=T}}^{(j)}, s_{y_{t=T}}^{(j)} \right) \right\| \right) \qquad \text{eqn 32}$$

of the centroid $\left(s_x^{(c)}, s_x^{(c)} \right)$ of all $j \in \{1, 2, \dots, J\}$ possible ending locations $\left(s_{x_{t=T}}^{(j)}, s_{y_{t=T}}^{(j)} \right)$. Programmatically, at relevant time steps in the filter, we computed the distance of each particle to $\left(s_x^{(c)}, s_y^{(c)} \right)$ and killed particles that exceeded the radius for that time step. This helped to pull particles from the forward filter into the region spanned by particles used to initialise the backward filter (and vice versa), which is important for smoothing. Otherwise, particle algorithms proceeded as previously described (see §4.4).

We used 150,000 particles for filtering and 2,000 particles for smoothing (see §6.2). As in simulation analyses (see §6), these choices represent a compromise between performance and computation time. The number of particles for filtering appeared to be generally sufficient, as we observed high convergence of the filters (see Main Text Results). A larger number of particles for smoothing would have helped to improve alignment between the two filters for smoothing (which was achieved for 75 % of filter runs), but was not possible given the computational resources available for this study. For best-guess analyses, the mean effective sample size per time step across all algorithm runs was 311 particles (AC: 250, DC: 349, ACDC: 385). Median total computation time was 2.0, 2.2 and 2.8 hours for the DC, AC and ACDC algorithms, respectively.

Supplementary figures

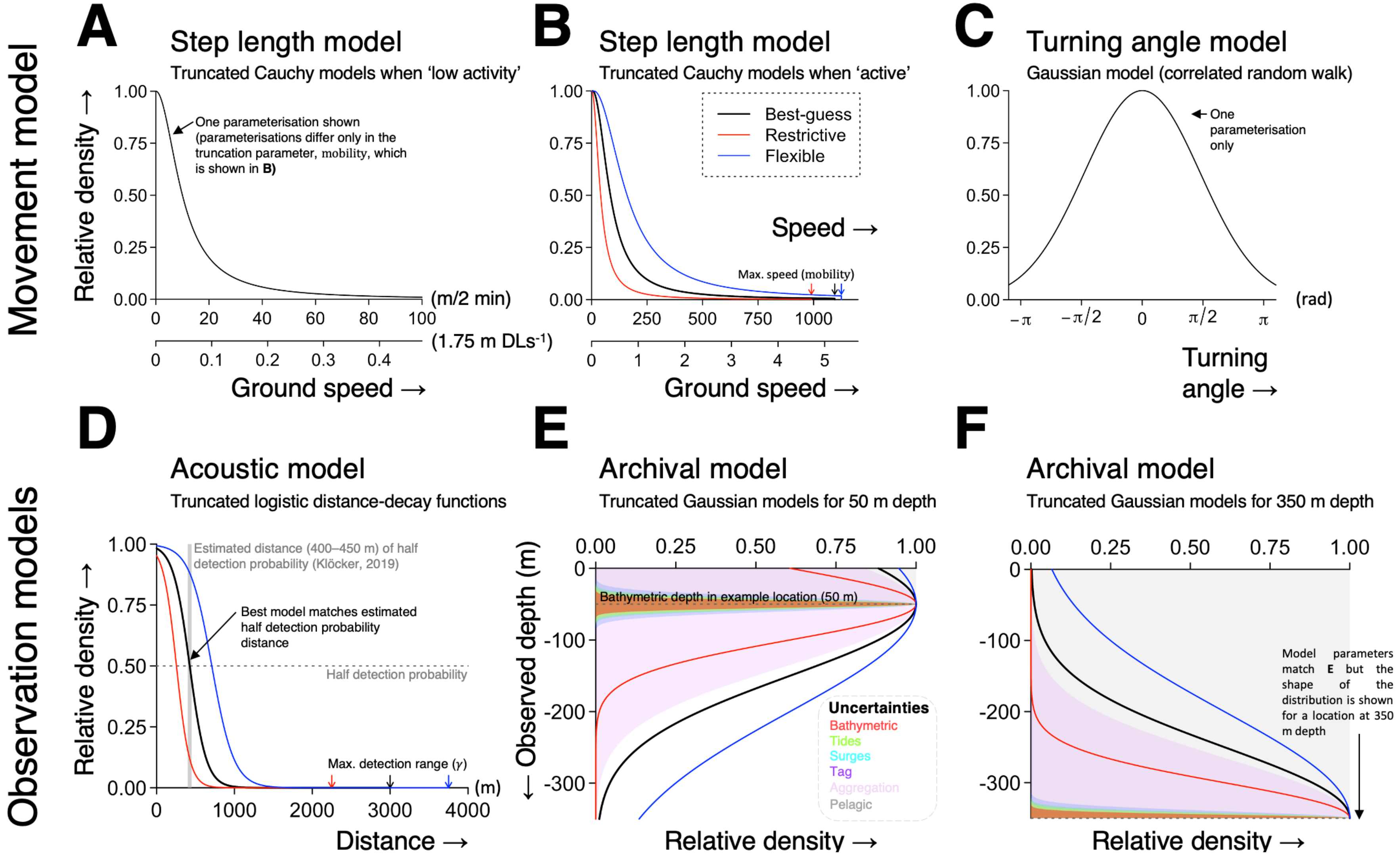

Fig. S1. A state-space model for tracking flapper skate. A–C illustrate the movement model; that is, the truncated Cauchy distribution of step lengths during (**A**) 'low activity' and (**B**) 'active' behavioural states and (**C**) the Gaussian distribution of turning angles. Step lengths are shown in units of metres per 2 min and disc lengths (DL) per second for a 1.75 m DL individual. **D–F** show the observation models for (**D**) acoustic and (**E–F**) depth observations at two bathymetric depths. The observation models evaluate the probability of the latent locations given (**D**) detection or non-detection at each operational receiver (the acoustic model) and (**E–F**) the depth observation (the depth model). The acoustic model is based on a truncated logistic, distance-delaying, detection-probability function. The depth model weighs the correspondence between a latent location's bathymetric depth and the observed depth. The required correspondence accounts for uncertainties in the bathymetric depth measurement, tidal range, storm surges, tag accuracy, bathymetry aggregation and potential pelagic movement (these are cumulative and shown by the coloured bands in **E** and **F**). Best-guess models are shown by the thick lines. Restricted and flexible model parameterisations (for the step length and observation models) are shown by the coloured lines and were used to examine algorithm sensitivity. All densities are scaled to a maximum value of one for visualisation. For full details, see Supplementary Text §4. For parameter values, see Table S4.

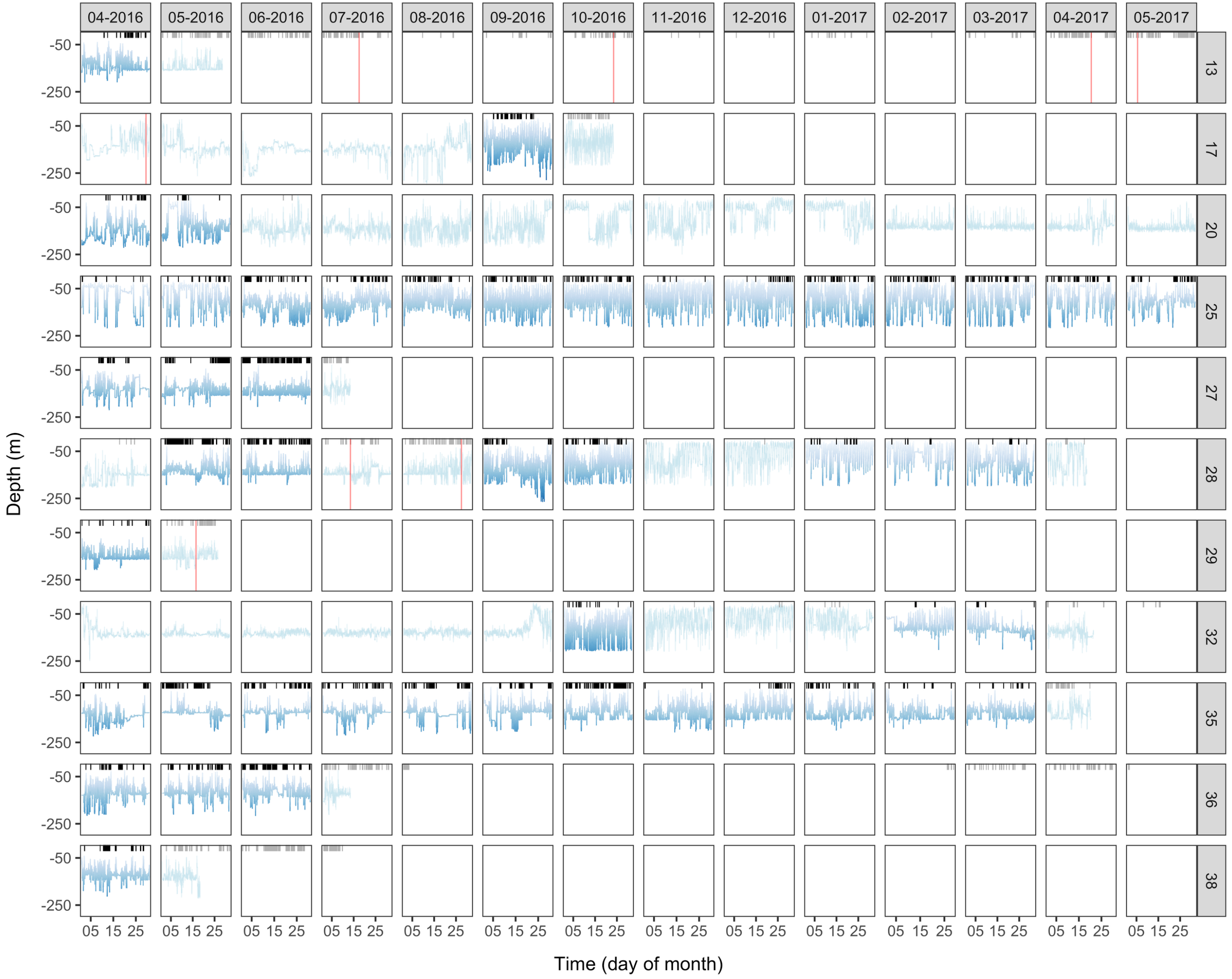

04-2016
05-2016
06-2016
07-2016
08-2016
09-2016
10-2016
11-2016
12-2016
01-2017
02-2017
03-2017
04-2017
05-2017
13
17
20
25
27
28
29
32
35
36
38
-50
-250
Depth (m)
05 15 25
Time (day of month)

Fig. S2. Observed time series. Panels distinguish individuals (rows) and months (columns) for which acoustic and archival (depth) data were collected from April 2016—May 2017. Points mark detections and lines mark depth observations. The colour scheme for depths is defined with respect to the bathymetry in the study area, following Fig. 1. For each individual, we modelled all months (panels) for which the individual was fully at liberty, with sufficient detection data (i.e., detections in at least two 7-day periods) and complete (uninterrupted) depth time series ($N = 48$ panels) (see Supplementary Text §7.1). Transparent panels indicate time series that did not meet the criteria for modelling. Recapture events, which interrupted individuals' time at liberty, are marked by red vertical lines. For a summary of modelled individuals, see Table S5.

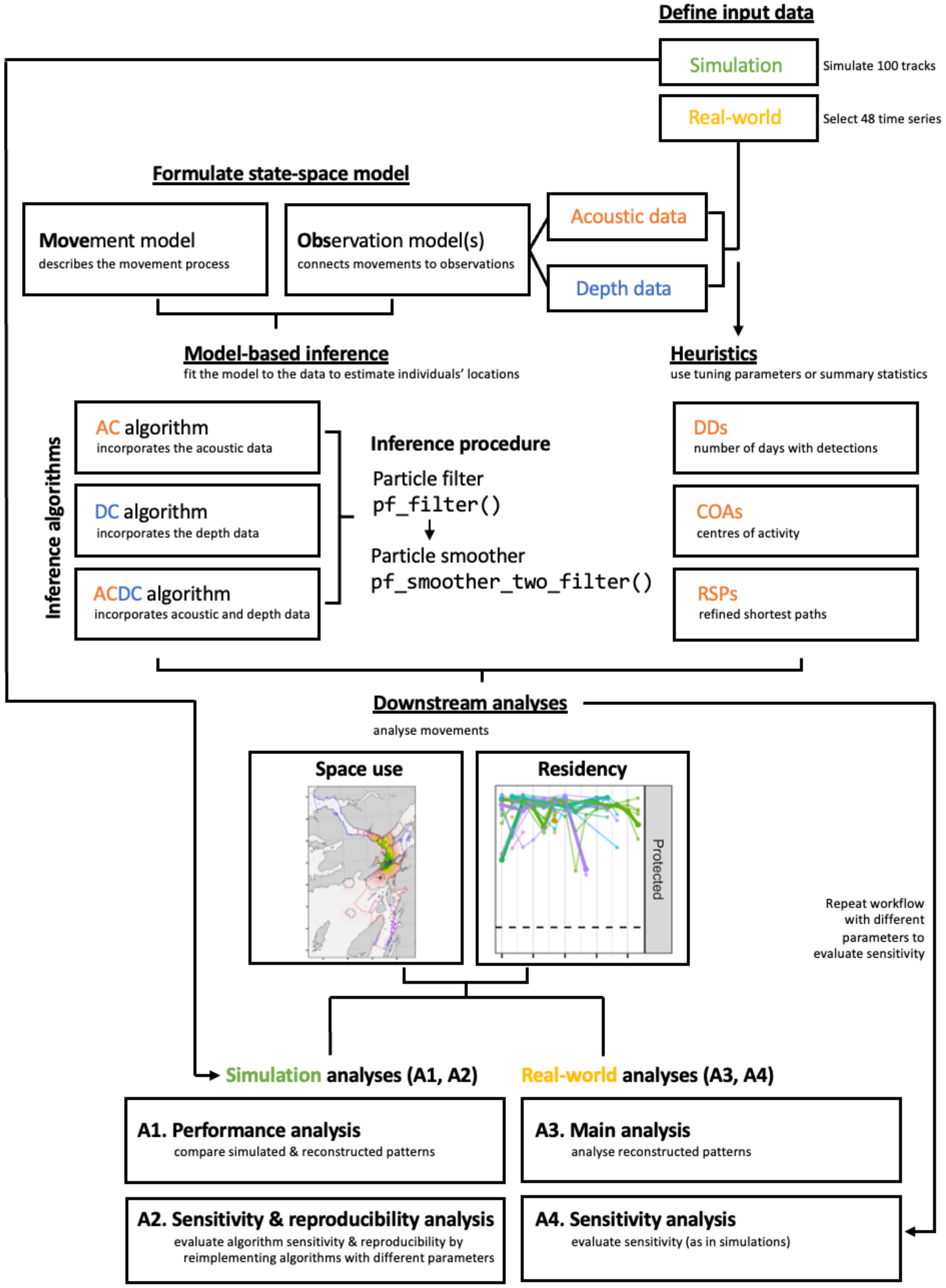
Define input data
Simulation
Simulate 100 tracks
Real-world
Select 48 time series
Formulate state-space model
Movement model
describes the movement process
Observation model(s)
connects movements to observations
Acoustic data
Depth data
Model-based inference
fit the model to the data to estimate individuals' locations
Heuristics
use tuning parameters or summary statistics
Inference algorithms
AC algorithm
incorporates the acoustic data
DC algorithm
incorporates the depth data
ACDC algorithm
incorporates acoustic and depth data
Inference procedure
Particle filter
pf_filter()
Particle smoother
pf_smoother_two_filter()
DDs
number of days with detections
COAs
centres of activity
RSPs
refined shortest paths
Downstream analyses
analyse movements
Space use
Residency
Protected
Repeat workflow with different parameters to evaluate sensitivity
Simulation analyses (A1, A2)
Real-world analyses (A3, A4)
A1. Performance analysis
compare simulated & reconstructed patterns
A2. Sensitivity & reproducibility analysis
evaluate algorithm sensitivity & reproducibility by reimplementing algorithms with different parameters
A3. Main analysis
analyse reconstructed patterns
A4. Sensitivity analysis
evaluate sensitivity (as in simulations)

Fig. S3. Summary of the workflow of simulation and real-world analyses. We conducted simulation-based and real-world analyses of animal movements. In both cases, we used heuristic and particle algorithms to analyse movements. Heuristic algorithms are based on turning parameters or summary statistics, whereas particle algorithms fit a state-space model to data (model-based inference). In simulation analyses (labelled A1 and A2), we compared patterns of space use and residency exhibited by simulated tracks to reconstructed patterns to examine algorithm performance (A1) and sensitivity (A2). In real-world analyses (labelled A3 and A4), we analysed tracking data from flapper skate. For full details, see Table S2. For a visual summary of the inference workflow, as implemented by the `patter` package, see ref. (*28*).

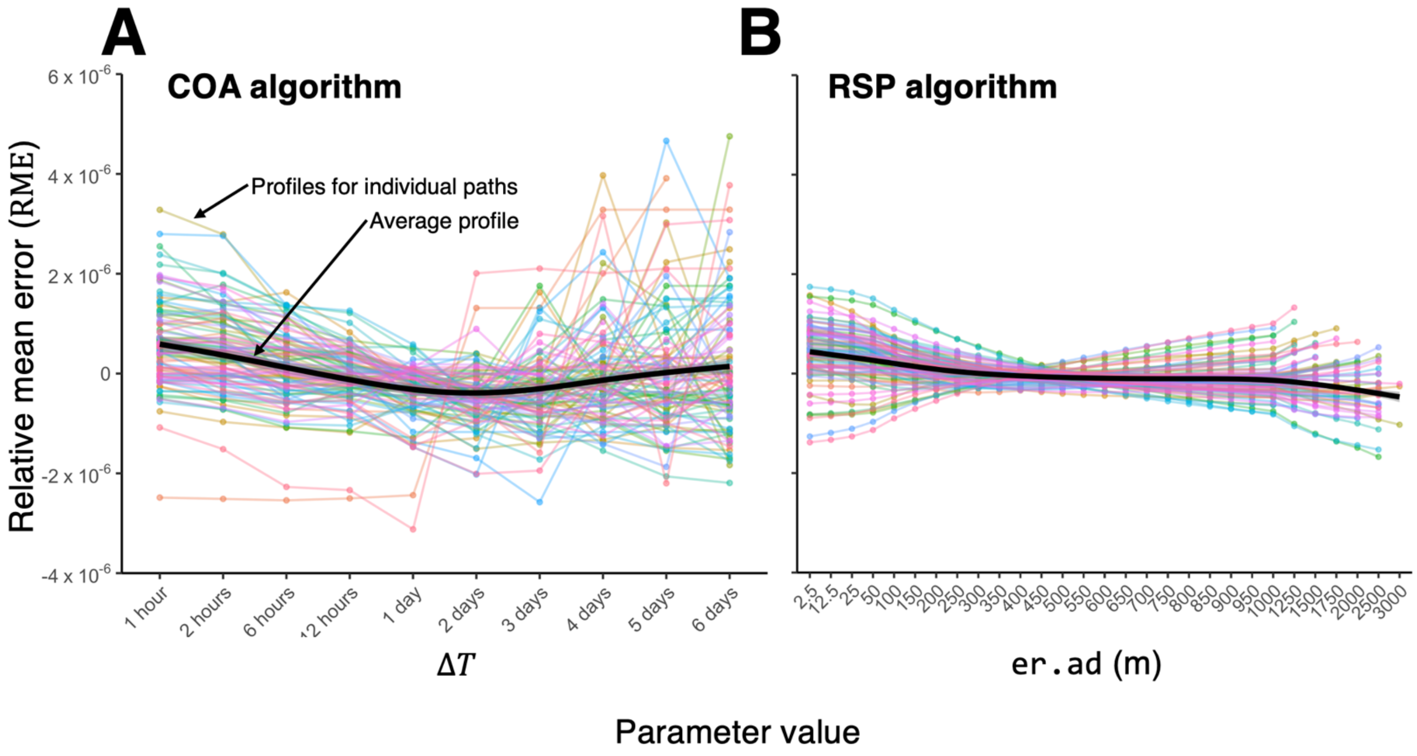

Fig. S4. Simulation analysis: heuristic algorithm performance profiles for (A) COAs and (B) RSPs. For each simulated path ($i \in \{1, \dots, 100\}$), the trend in relative mean absolute error (RME) across different values of ΔT (the time interval over which COAs are calculated) or `er.ad` (the rate at which uncertainty increases along RSPs) is shown. RME is the mean absolute difference between the UD for a simulated path and the corresponding reconstructed UD, centred by the average ME across all parameter values for that path. Note that the x-axes are non-linear. In each panel, the smooth line shows the expected RME as a function of the parameter value. Smooths were derived from generalised additive models of $RME_i \sim s(x_i)$, where s is a thin plate regression spline, fitted by mgcv (*103*). They are shown for visual aid only. We used these profiles to select parameter values for real-world analyses.

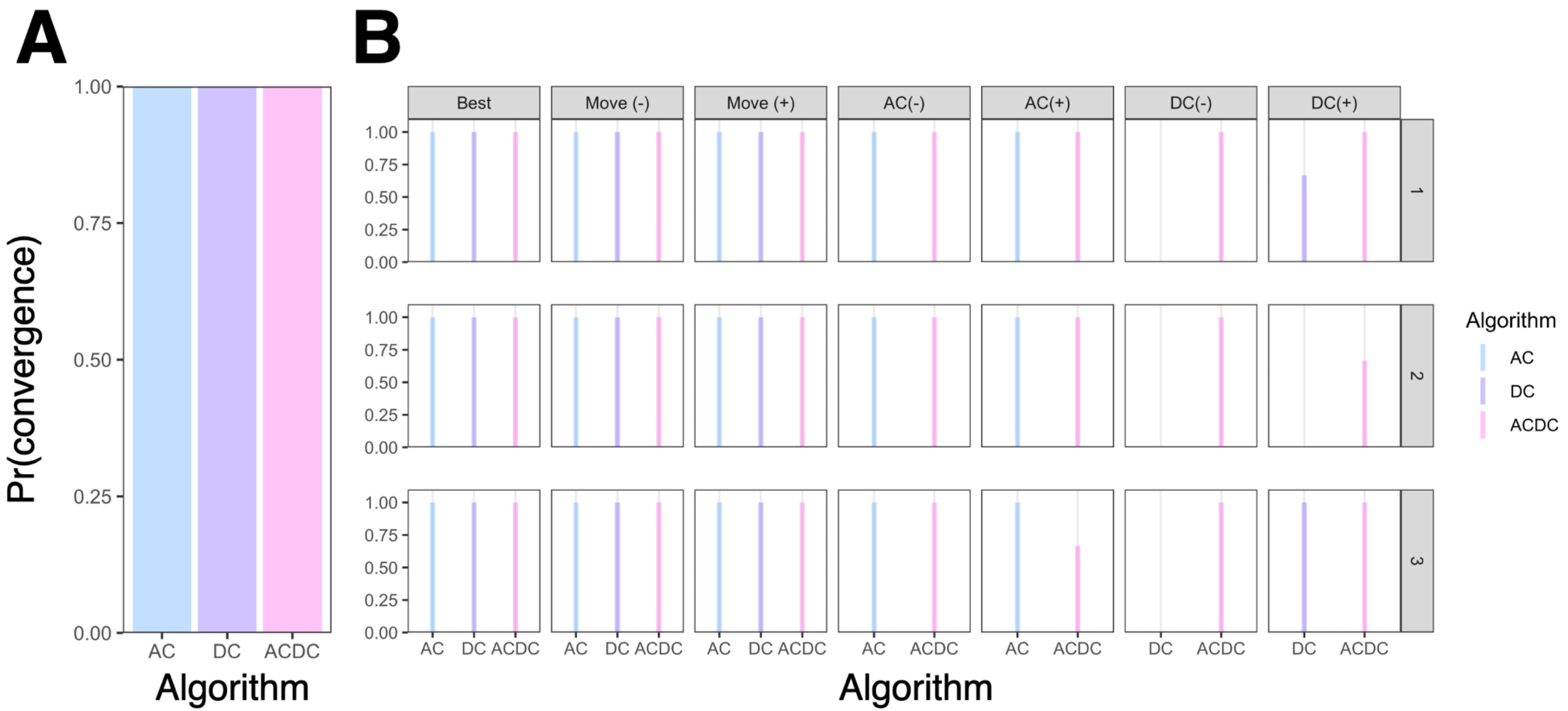

Fig. S5. **Simulation analysis: particle algorithm convergence properties for (A) performance and (B) sensitivity analyses. A** shows the proportion of algorithm runs that converged in the performance analysis. In this analysis, inference was performed once for each of the 100 simulated datasets using the data-generating parameter values. **B** shows the proportion of algorithm iterations that converged in the sensitivity analysis. In this analysis, inference was performed for each of first three simulated paths (rows) three times, using best-guess (data-generating) and mis-specified parameter values (columns). Mis-specified algorithm implementations were characterised by under (-) or over (+) estimation of the movement (Move), acoustic (AC) and depth (DC) observation model parameters, with other components held constant at data-generating values.

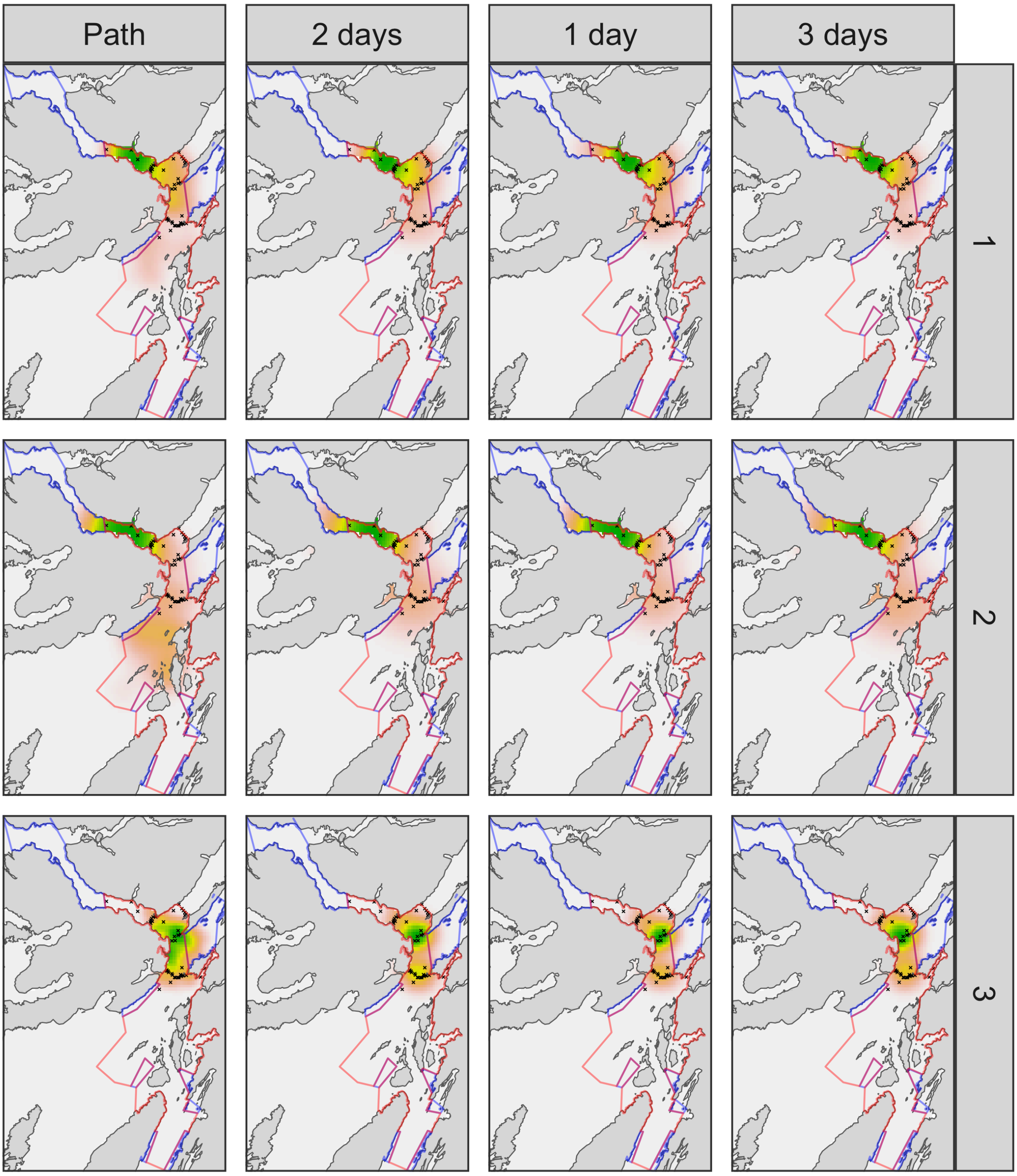

Fig. S6. Simulation analysis: COA algorithm performance and sensitivity. Rows distinguish simulated paths. For each simulated path, the 'true' UD and the UD from three COA algorithm implementations with different ΔT values (optimal, restrictive, flexible) are shown. Crosses mark receivers. The colour scale represents probability density and is shown between zero (white) and the maximum density (green) on each panel (following Figures 3–4). For clarity, maps are only shown within the extent of the MPA, as delineated by the blue and red polygons. These enclose protected zones open and closed to fisheries, respectively. Each panel is approximately 50 x 80 km (4000 km^2) in size.

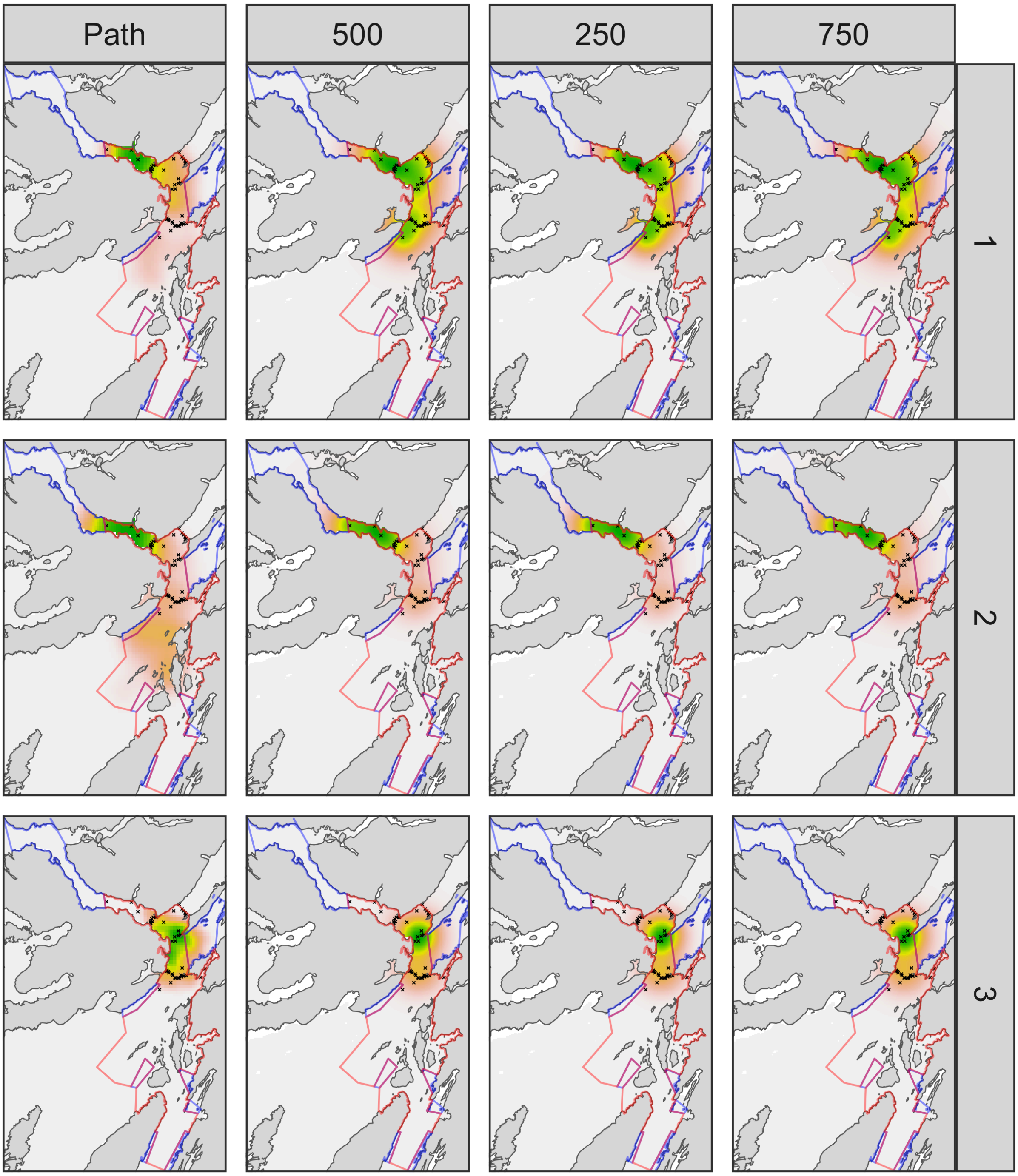

Fig. S7. **Simulation analysis: RSP algorithm performance and sensitivity, following Fig. S6.** Each panel is approximately 50 x 80 km (4000 km^2) in size.

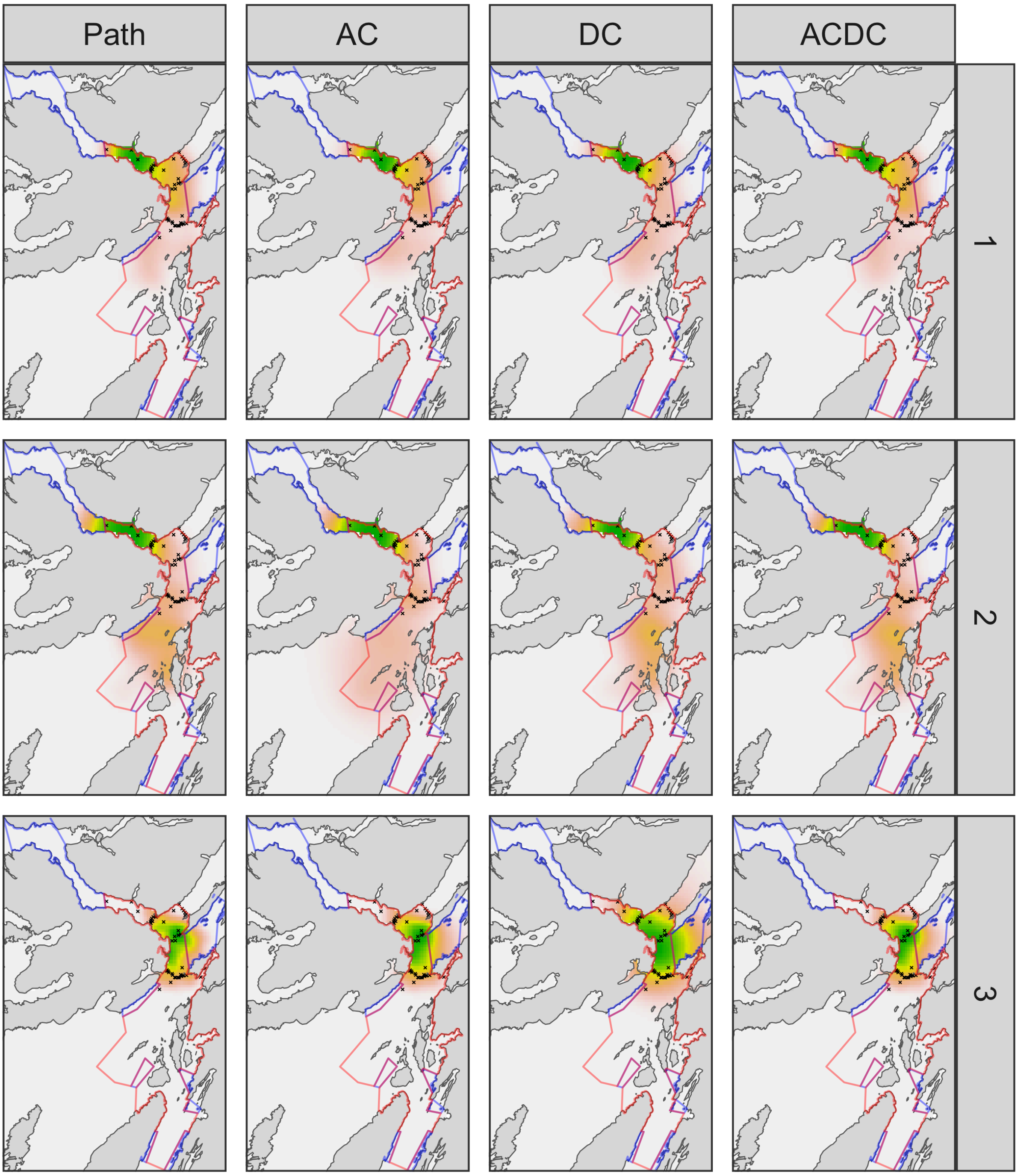

Fig. S8. Simulation analysis: particle algorithm performance, following Fig. S6. Rows distinguish simulated paths. For each simulated path, the ‘true’ UD and the UD from three particle algorithms (AC, DC and ACDC), correctly parameterised according to the data-generating process, are shown. Each panel is approximately 50 x 80 km (4000 km^2) in size.

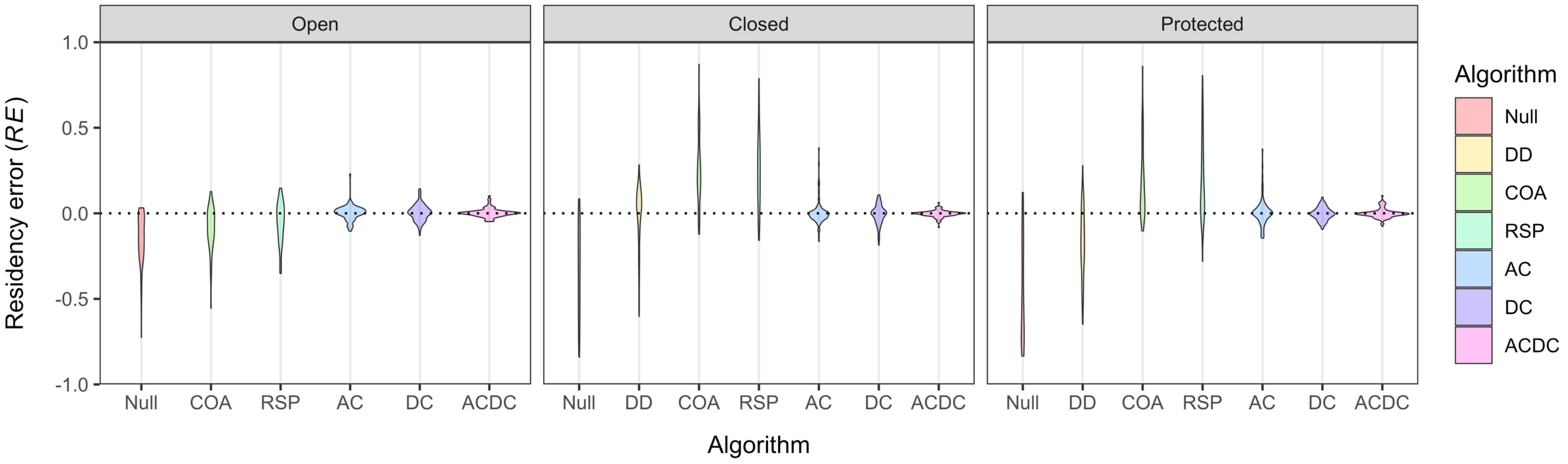

Fig. S9. Simulation analysis: model performance for estimating residency. Panels show residency error for zones within the MPA that are (a) open or (b) closed to fisheries and (c) the MPA as a whole. Residency error is the difference between estimated residency (the proportion of time spent in each zone) and the true value. Positive/negative values indicate overestimation/underestimation, respectively. Residency estimates were derived from performance analyses in which patterns of space use were reconstructed for each of the 100 simulated paths using optimal or data-generating parameter values. A null model and detection days (DD) are shown for context. The null model corresponds to the proportion of the study area in each zone. Detection days is the proportion of days with detections at receiver(s) (all of which are in closed areas). For heuristic (COA and RSP) and particle (AC, DC, ACDC) algorithms, residency was estimated (a) as the proportion of a UD's volume or (b) the proportion of particles, in each area, respectively. Each 'violin' shows the distribution of residency error between the UD for a simulated path and the UD reconstructed by the corresponding algorithm, across all 100 simulated paths. In performance analyses, inference was performed once for each of the 100 simulated datasets using heuristic algorithms with optimal parameters and particle algorithms (AC, DC and ACDC) with correctly defined (data-generating) parameters. Violin area is scaled by the number of algorithm runs that converged.

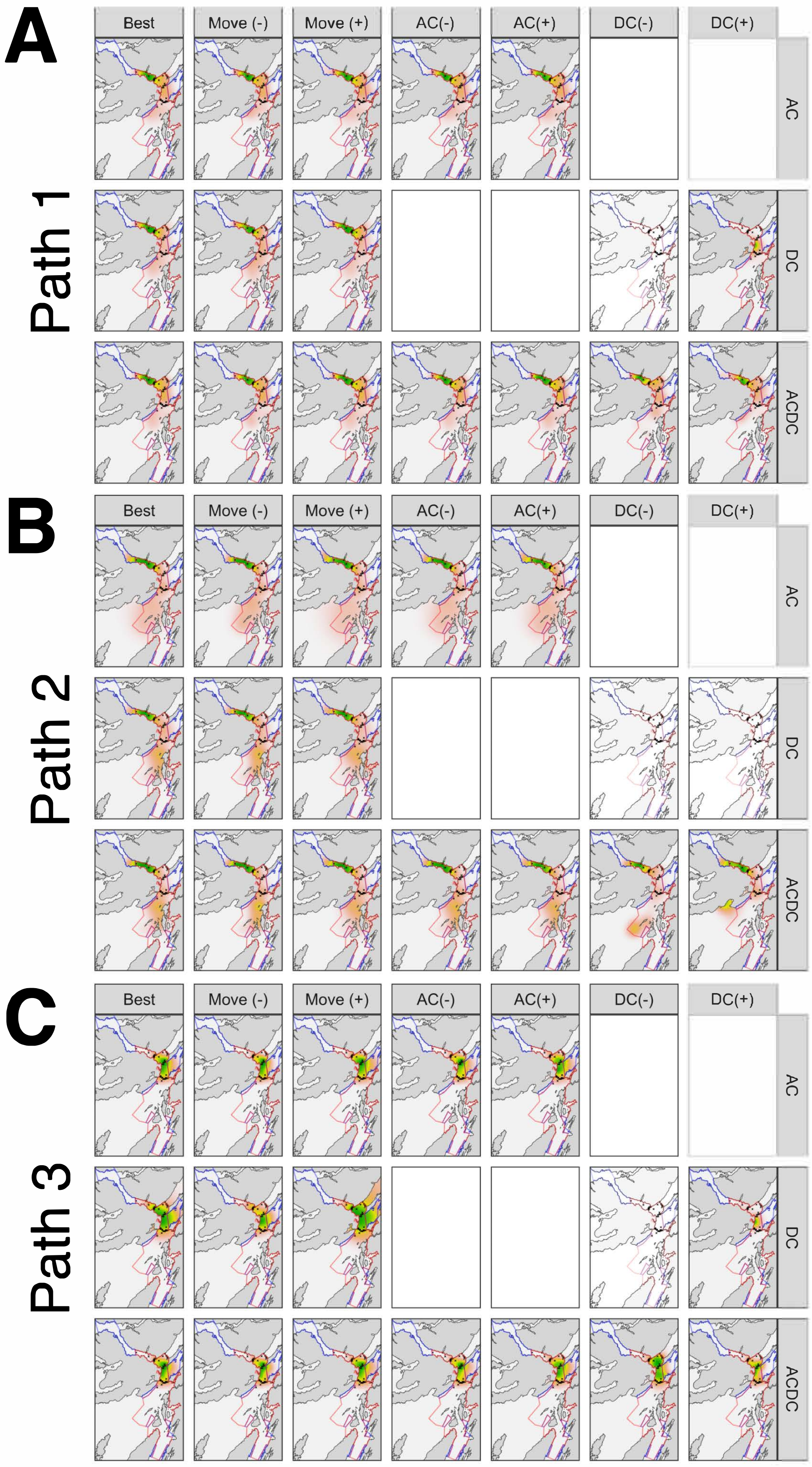

A
Path 1
B
Path 2
C
Path 3
Best
Move (-)
Move (+)
AC(-)
AC(+)
DC(-)
DC(+)
AC
DC
ACDC

Fig. S10. Simulation analysis: particle algorithm sensitivity. For each simulated path (1 [**A**], 2 [**B**] or 3 [**C**]), for each algorithm (row), UDs are shown for seven algorithm runs with different parameter settings (columns). UDs are shown for the first algorithm iteration (out of three for each parameter setting). Transparent panels indicate convergence failures. Blank panels indicate nonsensical parameter combinations (such as AC algorithm runs with under/overestimated depth observation model parameters). Each panel is approximately 50 x 80 km (4000 km^2) in size.

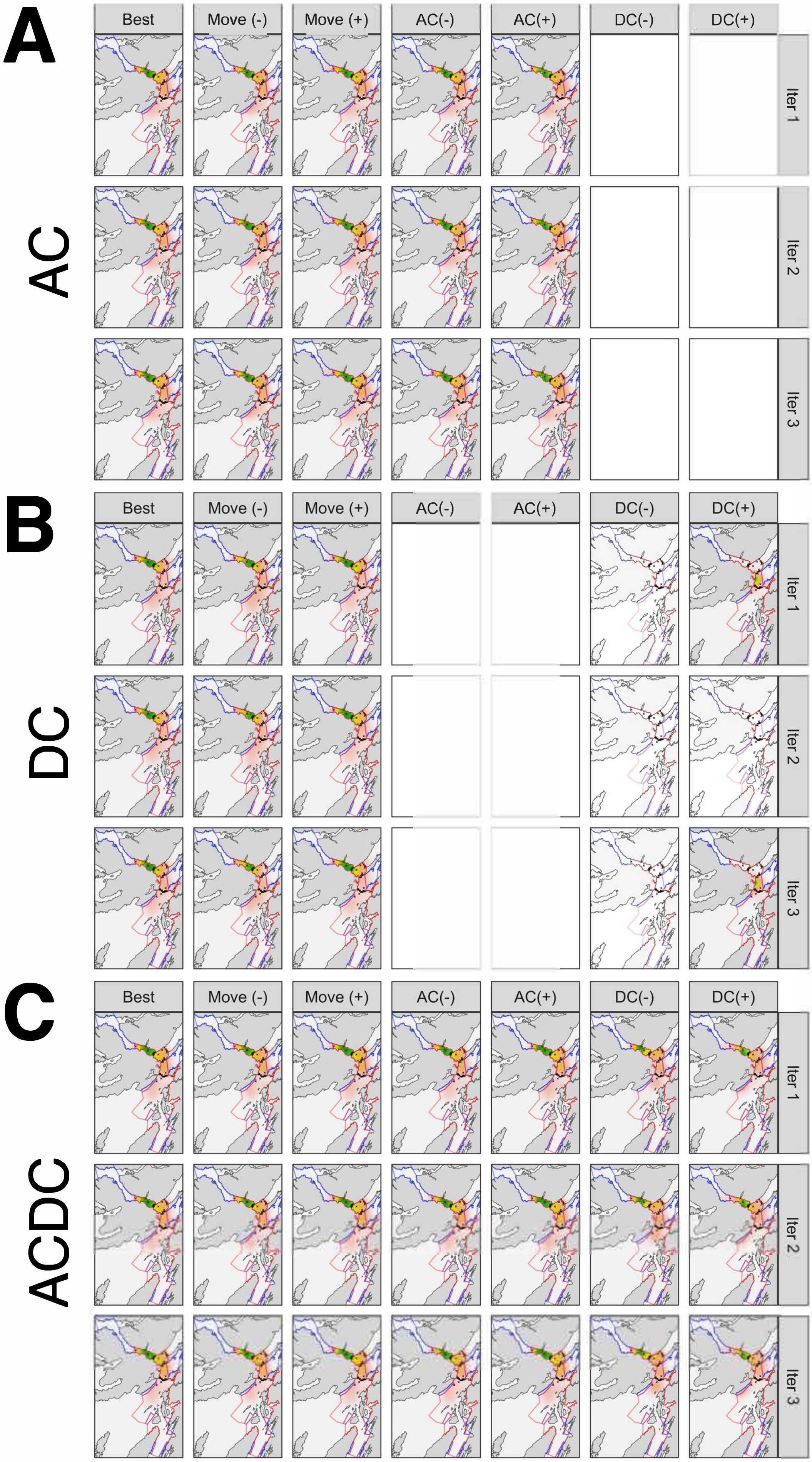
A
AC
Best
Move (-)
Move (+)
AC(-)
AC(+)
DC(-)
DC(+)
Iter 1
Iter 2
Iter 3
B
DC
C
ACDC

Fig. S11. Simulation analysis: particle algorithm repeatability, following Fig. S10. For the first simulated path, for each algorithm (row grouping), for three algorithm iterations (rows), UDs are shown for the seven parameter combinations (columns). Transparent panels indicate convergence failures and blank panels indicate nonsensical parameter combinations. Each panel is approximately 50 x 80 km (4000 km^2) in size.

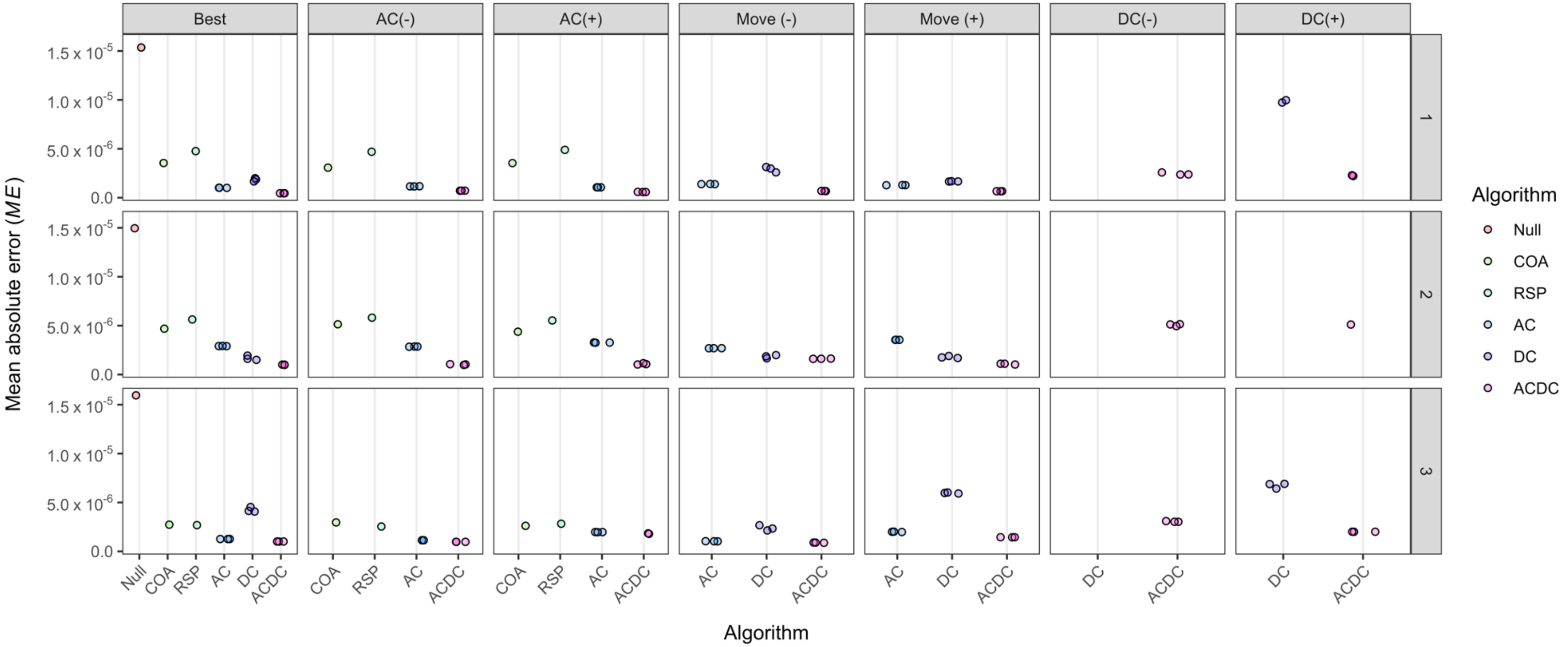

Fig. S12. Simulation analysis: algorithm sensitivity for mapping space use. Each panel shows the mean absolute error between the UD for a simulated path (rows) and the UD reconstructed by a selected algorithm parameterisation (column), including optimal/best-guess and restrictive/flexible parameterisations (columns). The null model is shown in the 'best' panel for reference. Particle (AC, DC and ACDC) algorithm implementations included a best (data-generating) parameterisation alongside under (-) or over (+) estimation of the movement (Move), acoustic (AC) and depth (DC) observation model parameters, with other components held constant at data-generating values. Particle algorithms were implemented independently three times to examine reproducibility (and three points are shown for each setting, except in the case of convergence failures). The two heuristic (COA and RSP) algorithms were implemented with optimal values (in 'best') and restricted/flexible parameterisations that are nominally included in the AC(-) and AC(+) panel columns, since these algorithms focus exclusively on acoustic detections. These algorithms are deterministic and only a single point is shown in relevant panels.

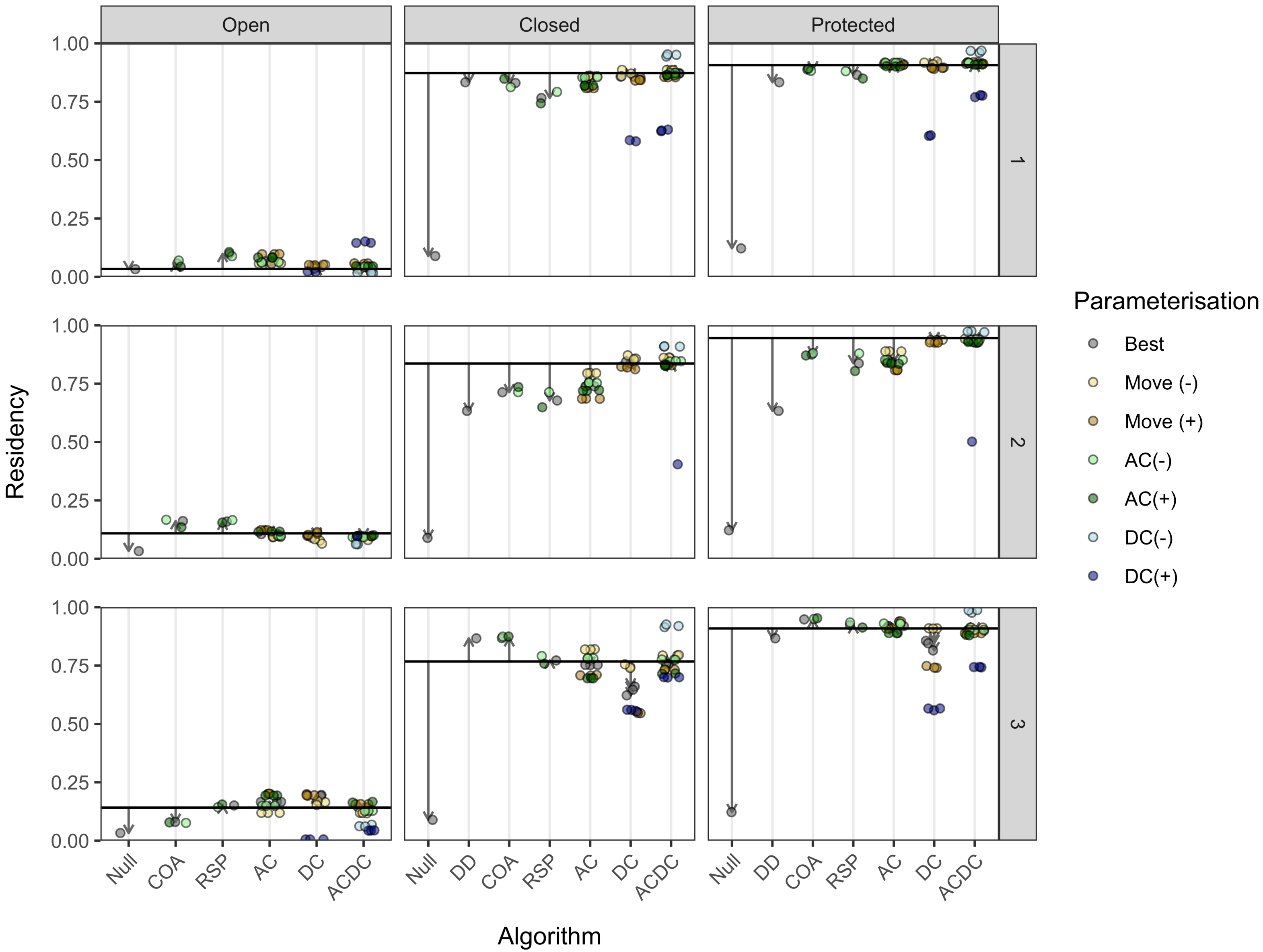

Open
Closed
Protected
1
2
3
1.00
0.75
0.50
0.25
0.00
Residency
Null
DD
COA
RSP
AC
DC
ACDC
Algorithm
Parameterisation
Best
Move (-)
Move (+)
AC(-)
AC(+)
DC(-)
DC(+)

Fig. S13. Simulation analysis: algorithm sensitivity for estimating residency. Each panel shows residency estimates from different algorithms in open or closed zones, or the MPA at large, for a given path (rows) and selected algorithm implementations (columns). Residency is estimated as the proportion of time spent in each area. The black horizontal line is the proportion of time steps the simulated path spent in each area. The null model corresponds to the proportion of the study area in each area. Detection days is the proportion of days with detections at receiver(s) (all of which are in closed zones). For heuristic (COA and RSP) and particle (AC, DC, ACDC) algorithms, residency was estimated (a) as the proportion of a UD's volume or (b) the proportion of particles, in each area, respectively. Particle (AC, DC and ACDC) algorithm implementations included a best (data-generating) parameterisation alongside under (-) or over (+) estimation of the movement (Move), acoustic (AC) and depth (DC) observation model parameters, with other components held constant at data-generating values. Particle algorithms were implemented independently three times to examine reproducibility (and three points are shown for each setting, except in the case of convergence failures). The two heuristic (COA and RSP) algorithms were implemented with optimal values and restricted/flexible parameterisations that are nominally included in the AC(-) and AC(+) categories, since these algorithms focus exclusively on acoustic detections. These algorithms are deterministic and only a single point is shown for the relevant settings. Vertical arrows point to the best residency estimate in each cluster. The spread of residency estimates around this point indicates the sensitivity, and reproducibility, of residency estimates under the different algorithm parameterisations.

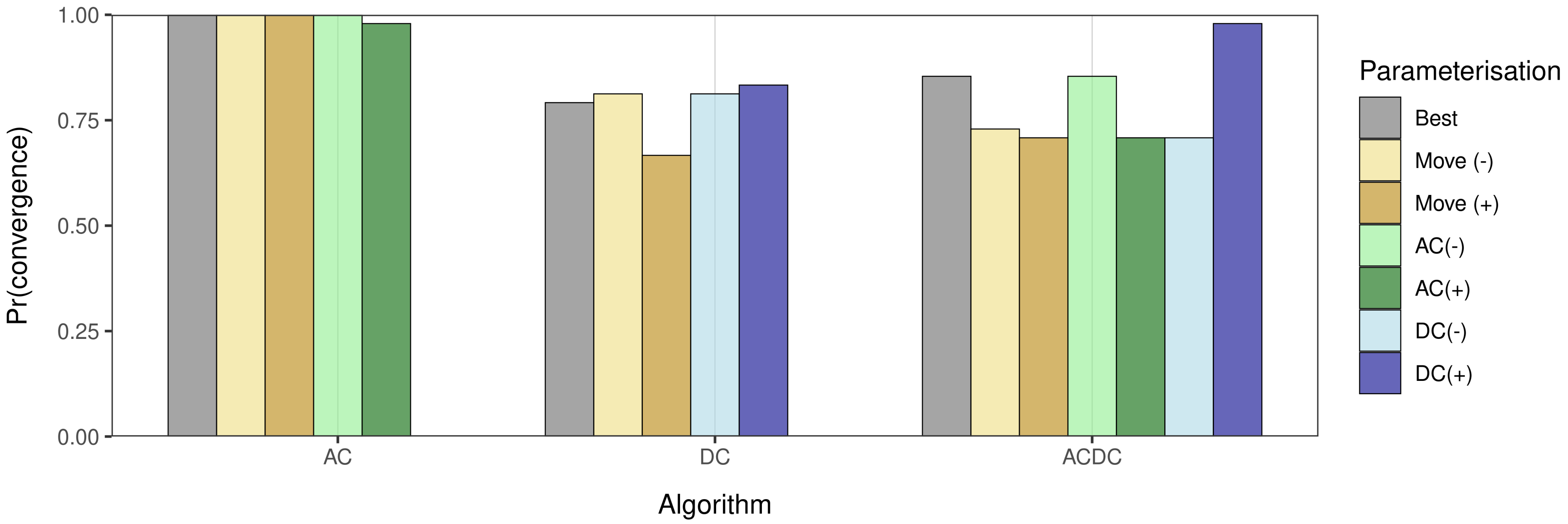

Fig. S14. Real-world analysis: particle algorithm convergence properties. For each particle algorithm, the proportion of algorithm runs that converged is shown for the five algorithm parameterisations (these are explained in Figures S12–13 with respect to simulation analyses).

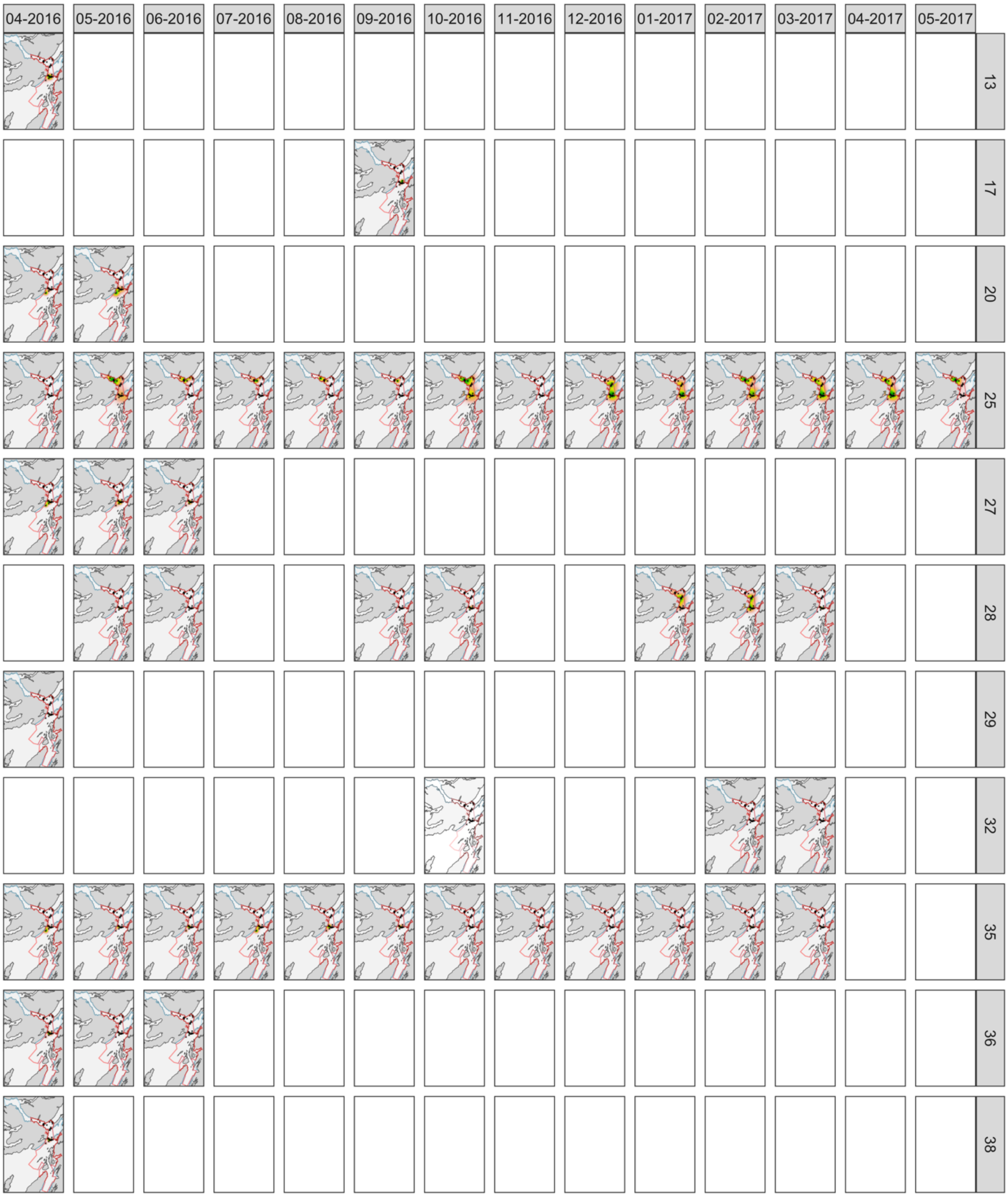

Fig. S15. Real-world analysis: COA algorithm maps from the main analysis. Each panel is the UD for a given individual (row) in a given month (column). As in earlier figures, crosses mark receivers. The colour scale represents probability density and is shown between zero (white) and the maximum density (green) on each panel. (In some panels, UDs from the COA algorithm are so concentrated they are 'hidden' by receivers.) For clarity, maps are only shown within the extent of the MPA, as delineated by the blue and red polygons (following earlier figures). These enclose protected zones open and closed to fisheries, respectively. Transparent panels indicate convergence failures (if applicable). Blank panels indicate individual/month combinations for which data were not collected or modelled (see Fig. S2). Each panel is approximately 50 x 80 km (4000 km^2) in size.

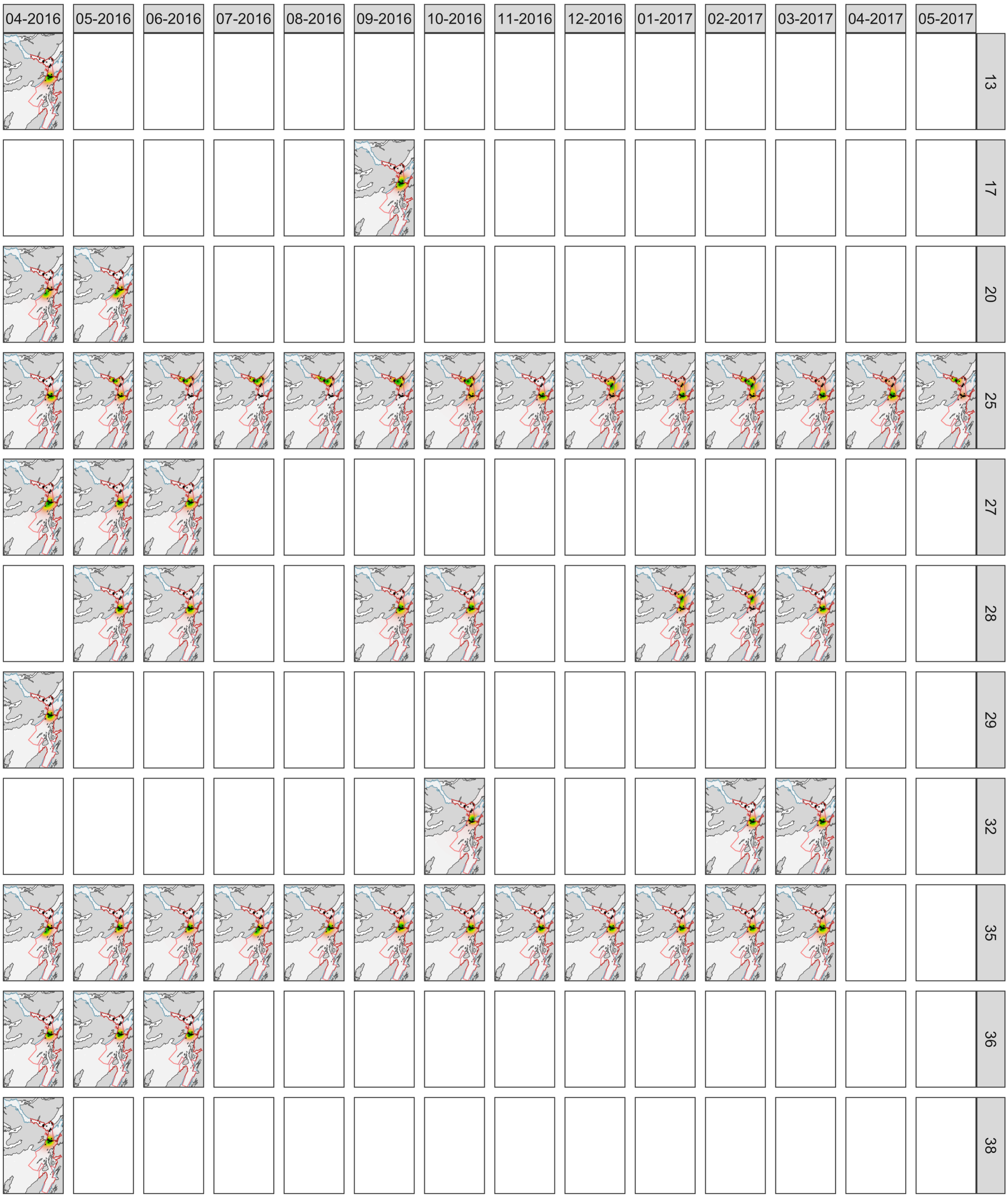

Fig. S16. Real-world analysis: RSP algorithm maps from the main analysis, following Fig. S15. Each panel is approximately 50 x 80 km (4000 km^2) in size.

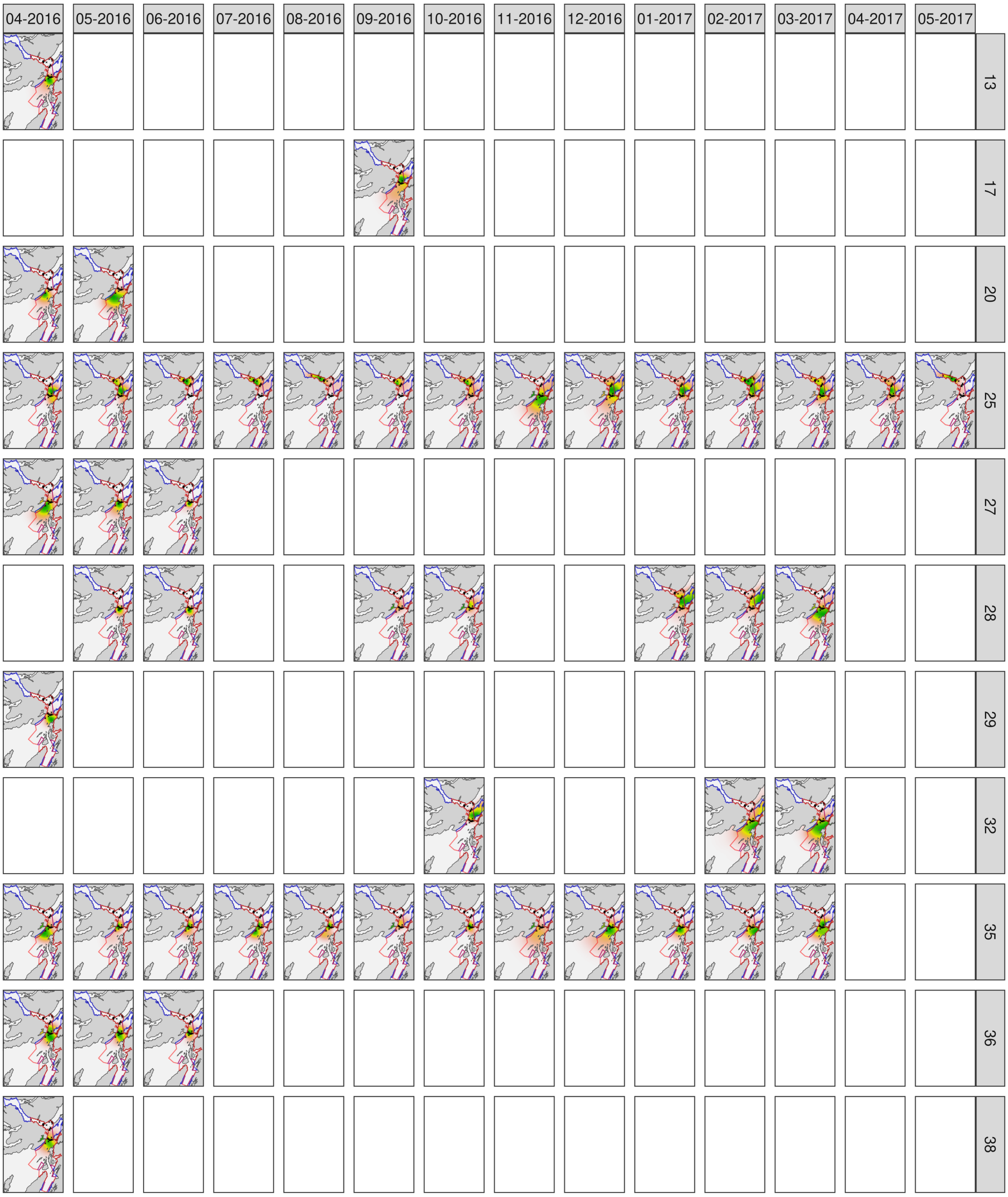

Fig. S17. Real-world analysis: AC algorithm maps from the main analysis, following Fig. S15. Each panel is approximately 50 x 80 km (4000 km^2) in size.

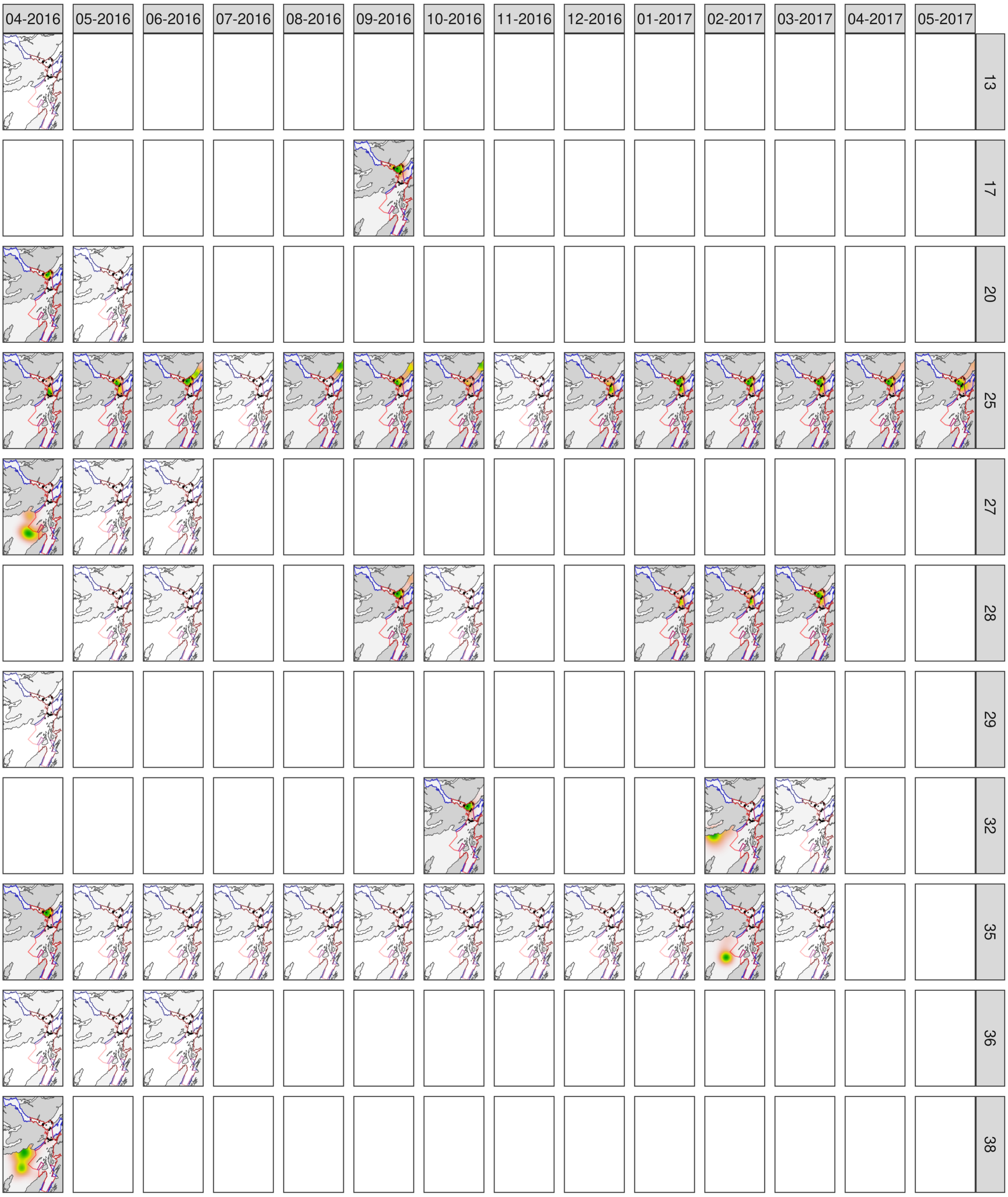

Fig. S18. Real-world analysis: DC algorithm maps from the main analysis, following Fig. S15. Each panel is approximately 50 x 80 km (4000 km^2) in size.

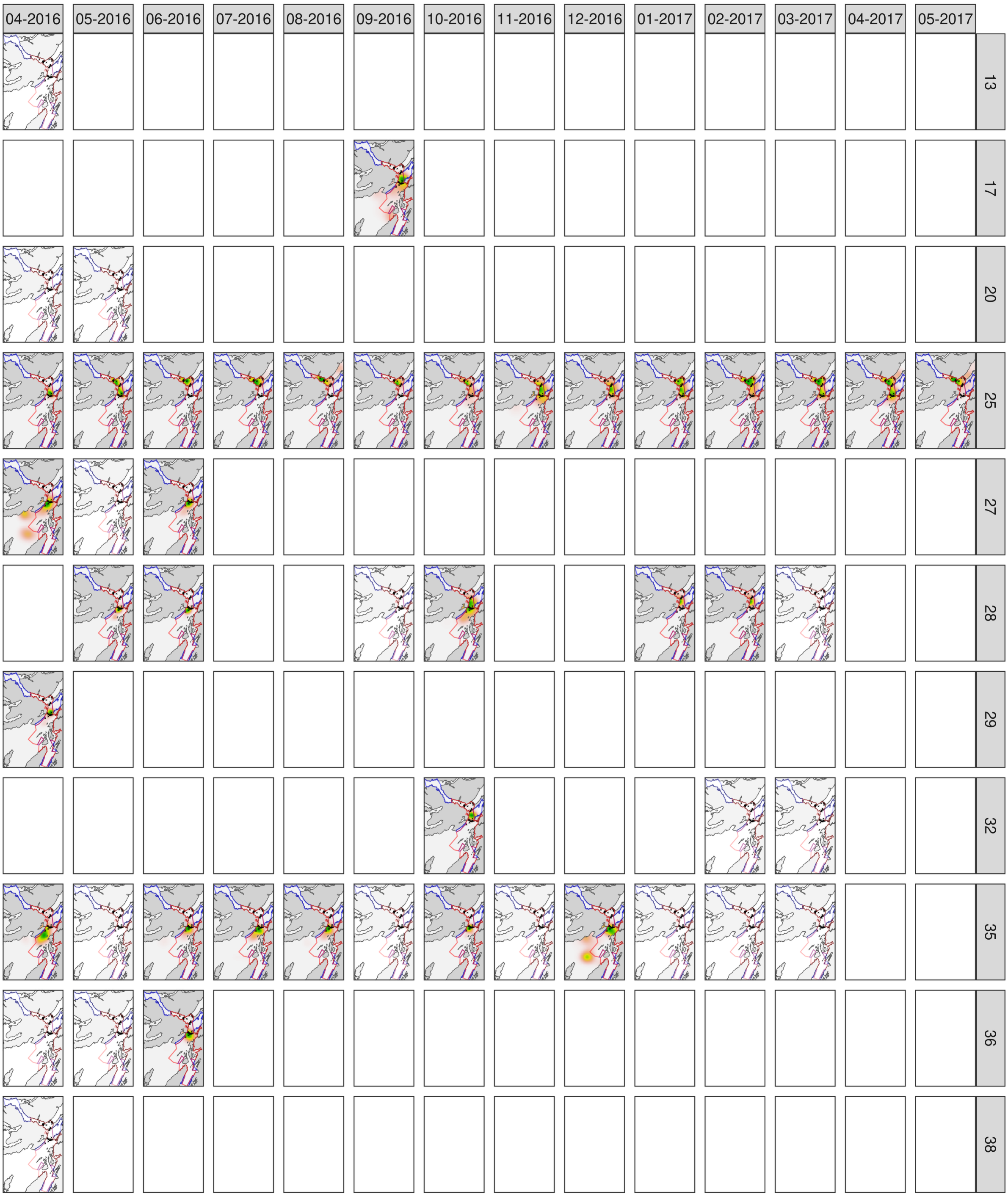

Fig. S19. Real-world analysis: ACDC algorithm maps from the main analysis, following Fig. S15. Each panel is approximately 50 x 80 km (4000 km^2) in size.

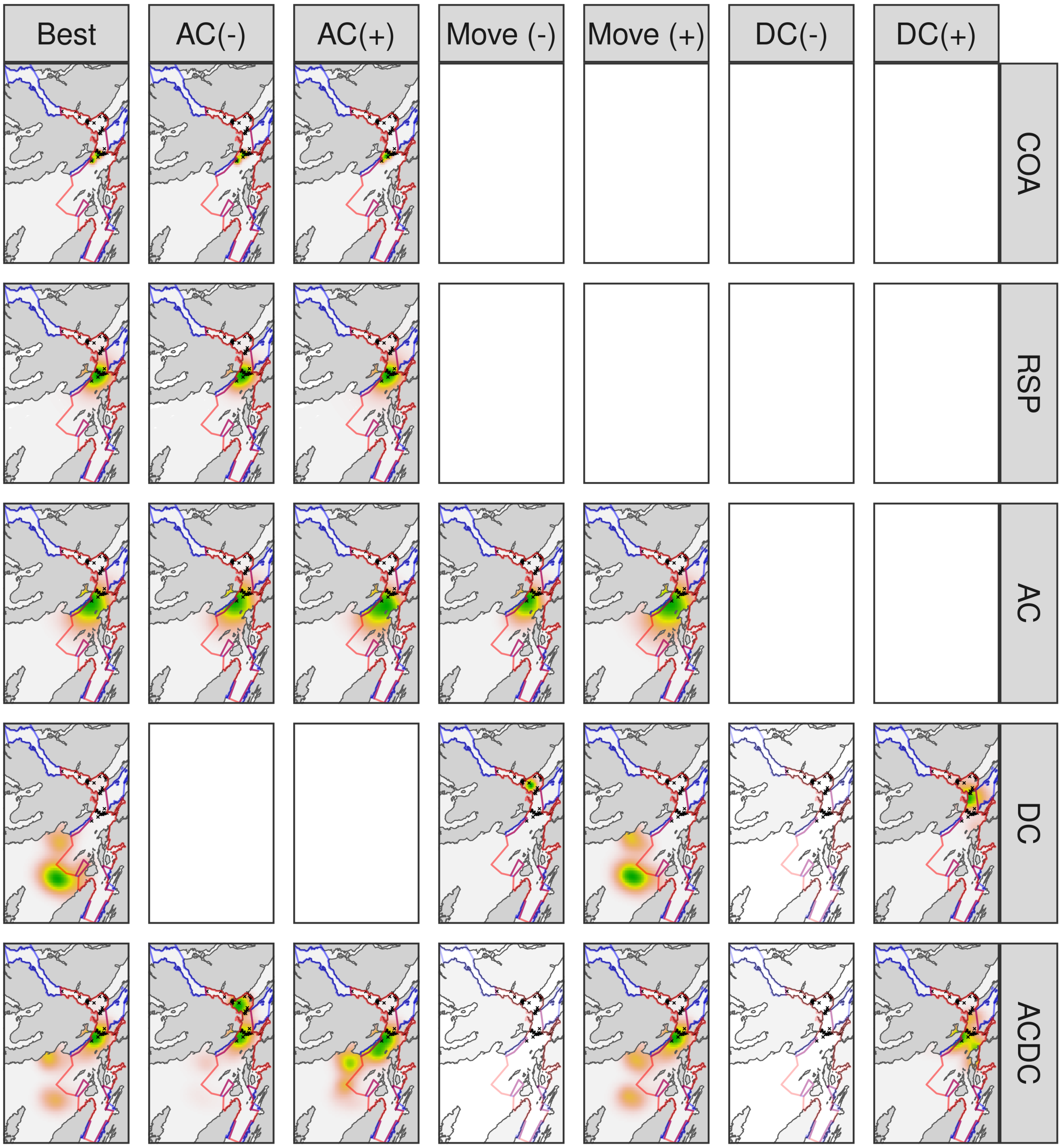

Fig. S20. Real-world analysis: sensitivity in maps of space use. Each panel shows the UD for an example individual (27) in an example month (April 2016) according to a particular algorithm (row) and parameterisation (column). Figure properties follow earlier figures. Particle (AC, DC and ACDC) algorithm implementations included a best (data-generating) parameterisation alongside restrictive (-) or flexible (+) settings for the movement (Move), acoustic (AC) and depth (DC) observation models, with other components held constant at data-generating values. The two heuristic (COA and RSP) algorithms were implemented with optimal values and restricted/flexible parameterisations that are nominally included in the AC(-) and AC(+) panel columns, since these algorithms focus exclusively on acoustic detections. Transparent panels indicate convergence failures. Blank panels indicate nonsensical algorithm–parameter combinations. Each panel is approximately 50 x 80 km (4000 km^2) in size.

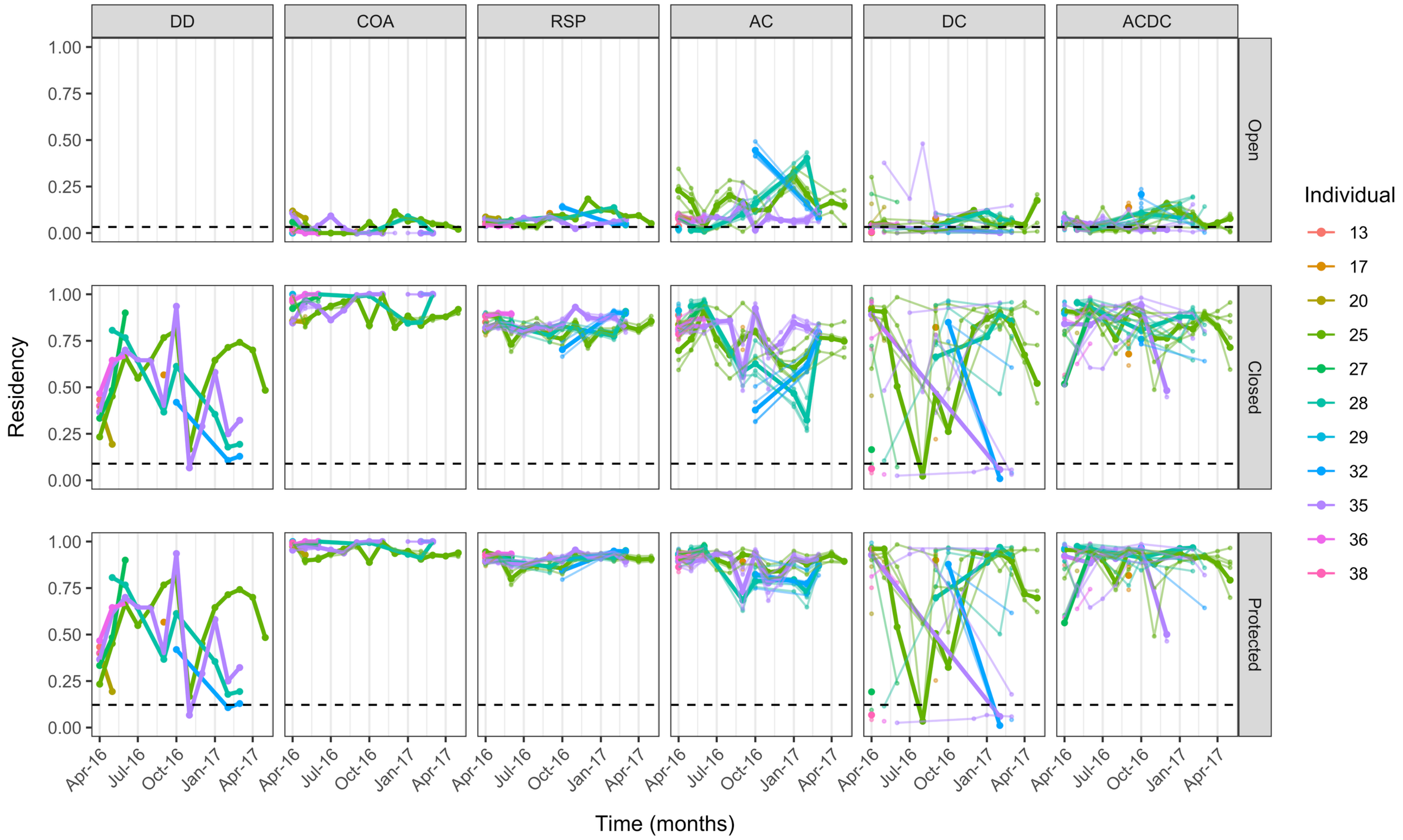
DD
COA
RSP
AC
DC
ACDC
Open
Closed
Protected
Residency
1.00
0.75
0.50
0.25
0.00
Apr-16
Jul-16
Oct-16
Jan-17
Apr-17
Time (months)
Individual
13
17
20
25
27
28
29
32
35
36
38

Fig. S21. Real-world analysis: sensitivity in residency estimates. Panels show individual residency time series for a given management area (row) and algorithm (column). The terms 'Open', 'Closed' and 'Protected' refer to zones within the MPA that are open or closed to fisheries and the entire MPA. Residency is the expected proportion of time spent in each area (in a given month). Thick points/lines are best-guess residency estimates; thinner points/lines are estimates from other algorithm parameterisations (sensitivity analysis). Estimates are only shown for months with sufficient data (hence gaps in some time series). Within panels, the feature of interest is the overall spread of estimates (rather than individual trajectories). Horizontal lines are null model estimates.

Supplementary tables

Table S1. Bathymetric data sources. For each region, the bathymetry datasets are listed. For the study system, the base dataset was sourced from ref. (*63*). In selected regions, gaps in this dataset were filled with additional datasets sourced from Admiralty's Seabed Mapping Service. The Scotland-wide dataset was obtained from Digimap. Data assembly is described in Supplementary Text §1.

Region	Dataset
Study system, Firth of Lorn	Howe et al. (*63*)
Study system, Coll and Tiree	2009 HI1257 The Isles of Eigg and Muck Blk4 2m SB
	2009 HI1297 Western App to Small Isles Blk1 2m SB
	2009 HI1297 Western App to Small Isles Blk1 4m SB
	2009 HI1297 Western App to Small Isles Blk1 8m SB
	2009 HI1297 Western App to Small Isles Blk2 2m SB
	2009 HI1297 Western App to Small Isles Blk2 4m SB
	2009 HI1297 Western App to Small Isles Blk2 8m SB
	2009 HI1297 Western App to Small Isles Blk3 2m SB
	2009 HI1297 Western App to Small Isles Blk3 4m SB
	2009 HI1297 Western App to Small Isles Blk3 8m SB
	2009 HI1297 Western App to Small Isles Blk4 2m SB
	2009 HI1297 Western App to Small Isles Blk4 4m SB
	2009 HI1297 Western App to Small Isles Blk4 8m SB
	2010 HI1297 Western App to Small Isles Blk4 Addendum 2m SB
	2010 HI1297 Western App to Small Isles Blk4 Addendum 4m SB
	2012 HI1297 Inner App to Small Isles Blk6 2m SB
	2012 HI1297 Inner App to Small Isles Blk6 4m SB
	2012 HI1297 Inner App to Small Isles Blk6 8m SB
	2012 HI1297 Pt 2 Inner App to Small Isles Blk2 2m SB
	2012 HI1297 Pt 2 Inner App to Small Isles Blk2 4m SB
	2012 HI1297 Pt 2 Inner App to Small Isles Blk2 8m SB
	2012 HI1297 Pt 2 Inner App to Small Isles Blk4 2m SB
	2012 HI1297 Pt 2 Inner App to Small Isles Blk4 4m SB
	2012 HI1298 Passage of Tiree Blk B 2m SB
Study system, Islay	2010 HI1329 Sound of Islay Blk1 2m SB
	2010 HI1329 Sound of Islay Blk2 2m SB
	2012 2014-249146 South West Islay 2m SB
	2012 HI1362 Outer Approaches to Firth of Lorne Blk E 2m SB
	2012 HI1362 Outer Approaches to Firth of Lorne Blk E 4m SB
	2013 HI1370 Approaches to Sound of Jura 2m SB
	2013 HI1370 Approaches to Sound of Jura 4m SB
	2013 HI1371 Sound of Jura 2m SB
	2013 HI1371 Sound of Jura 4m SB
Study system, Loch Creran	2017 2021-230548 Loch Creran 2m SDTP
Study system, Loch Etive	2014 HI1441 Loch Etive 2m SDTP
	2014 HI1441 Loch Etive 4m SDTP
	2014 HI1441 Loch Etive 8m SDTP
	2012 HI1373 Loch Linnhe Blk A 2m SB

**Region**	**Dataset**
Study system, Loch Linnhe	2012 HI1373 Loch Linnhe Blk A 4m SB
	2012 HI1373 Loch Linnhe Blk A 8m SB
	2012 HI1373 Loch Linnhe Blk A 12m SB
	2012 HI1373 Loch Linnhe Blk B 2m SB
	2012 HI1373 Loch Linnhe Blk B 4m SB
	2012 HI1373 Loch Linnhe Blk B 8m SB
	2012 HI1373 Loch Linnhe Blk C 2m SB
	2012 HI1373 Loch Linnhe Blk C 4m SB
	2012 HI1373 Loch Linnhe Blk C 8m SB
	2012 HI1373 Loch Linnhe Blk C 12m SB
Study system, Lochgilphead	2015 2015-209463 Claonaig Slipway
	2015 2015-209463 Lochranza
	2016 2016-298157 Port Crannaich 2m
	2016 2017-310993 Loch Fyne Otter Spit
	2016 2020-191663 Loch Fyne Wreck and Beaufort Rock 2m SDTP
	2020 2021-262711 Carradale Harbour
	2021 HI1680 Kilbrannan Sound 0-40m 2m SDTP
	2021 HI1680 Kilbrannan Sound 36-152m 4m SDTP
	2022 2022-067840 Claonaig Slipway
	2022 2022-067840 Lochranza
	2023 2023-150089 Claonaig Slipway
	2023 2023-129344 Carradale Harbour
Scotland	marine-dem-6-sec_3965365

Table S2. Summary of the workflow of simulation and real-world analyses.

Workflow summary
**Simulation experiments**<br>***Examine algorithm behaviour (relative performance, sensitivity and reproducibility) in our study system and inform real-world analyses***<br><br>**A1. Performance evaluation**<br>***Compare the performance of heuristic and particle algorithms for reconstructing patterns of space use and estimating residency, using 100 simulated paths/observational datasets***<br><br>**A1.1. Algorithm parameterisation**<br>**A1.1.1.** ***Heuristic algorithms.*** Select optimal parameters for the COA (ΔT) and RSP (`er.ad`) algorithms from profiles of the mean absolute error (ME), between UDs for the simulated paths and UDs reconstructed with different parameter values (Fig. S4).<br>**A1.1.2.** ***Particle algorithms.*** Use data-generating (i.e., best-guess) parameters (chosen for flapper skate, based on biological expertise).<br><br>**A1.2. Algorithm implementation.** Implement the algorithms with optimal or best-guess parameters and validate convergence (Fig. S5).<br><br>**A1.3. Algorithm performance**<br>**A1.3.1.** ***Maps of space use: visual evaluation.*** Visualise maps of space use for the first three paths (Figures S6–8).<br>**A1.3.2.** ***Maps of space use: quantitative evaluation.*** Compute the distribution of ME (from the UD for each simulated path and the reconstructed UD), across all paths by algorithm (Fig. 2).<br>**A1.3.3.** ***Residency***. Compute the distribution of residency error (RE) as the difference between estimated and true residency, across all paths by algorithm, in open/closed zones and the entire MPA (Figures 2 and S9).<br><br>**A2. Sensitivity and reproducibility evaluation**<br>***Compare algorithm sensitivity to parameter choices for maps of space use and residency estimates, using the first three simulated paths/observational datasets***<br><br>**A2.1. Algorithm implementation.** Implement the algorithms with restrictive and flexible settings. For COAs and RSPs, restrict/inflate $\Delta T \in \{2\text{ days}, 1\text{ day}, 3\text{ days}\}$ and $\text{er.ad} \in \{500, 250, 750\}$. For particle algorithms, restrict/inflate the movement model, acoustic observation model and/or depth observation model parameters, while holding other parameters constant. Repeat particle algorithms independently three times to examine reproducibility.<br><br>**A2.2. Algorithm sensitivity and reproducibility**<br>**A2.2.1.** ***Maps of space use: visual evaluation.*** Compare maps of space use using optimal/best-guess, restrictive and flexible parameters, and across multiple iterations of the particle algorithms (Figures S6–7 and S10–11).<br>**A2.2.2.** ***Maps of space use: quantitative evaluation.*** Compute ME for each algorithm parameterisation/iteration (Fig. S12).

Workflow summary
**A2.2.3.** ***Residency.*** Compute RE for each algorithm parameterisation/iteration (Fig. S13).
**Real-world analyses**<br>***Reconstruct patterns of space use and residency of flapper skate in the Loch Sunart to the Sound of Jura MPA***<br><br>**A3. Main analyses**<br>***Reconstruct patterns of space use and estimate residency via heuristic and particle algorithms for each individual and month with sufficient observations, using optimal/best-guess algorithm parameterisations***<br><br>**A3.1. Algorithm implementation.** Implement the algorithms with optimal/best-guess parameters (as in simulations) and validate convergence (Fig. S14).<br><br>**A3.2. Analyses**<br>**A3.2.1.** ***Maps of space use: visual evaluation.*** Reconstruct and visualise patterns of space use for each time series using best-guess algorithm parameterisations (Figures 3 and S15–19).<br>**A3.2.2.** ***Maps of space use: summary.*** Reconstruct an overall map of space use and visually assess the overlap with presence records from angling (Fig. 4).<br>**A3.2.3.** ***Residency***. Estimate residency within open/closed zones and the entire MPA (Fig. 5).<br><br>**A4. Sensitivity analyses**<br>***Examine sensitivity to parameter choices for maps of space use and residency estimates***<br><br>**A4.1. Algorithm implementation.** Implement the algorithms with restrictive and flexible parameters (as in simulations) and validate convergence (Fig. S14).<br><br>**A4.2. Analyses**<br>**A4.2.1.** ***Maps of space use.*** Reconstruct patterns of space use and visually compare sensitivity to algorithm parameterisation (Fig. S20).<br>**A4.2.2.** ***Residency***. Estimate residency in open/closed zones and the entire MPA, and visually compare sensitivity to algorithm parameterisation (Fig. S21).

Table S3. Summary of notation. The essential notation in the Main Text and the Supplementary Text is listed, ordered alphabetically. Example references to Supplementary Text equations are provided. Notation broadly follows ref. (*19*) where further details are provided.

Notation	Description
A	A set/subset of locations in the study area compatible with the initial observation(s). See eqn 3.
α	A linear coefficient in a logistic, distance-decaying, detection-probability model. See eqn 12.
$\mathrm{behaviour}$	The behavioural state of the individual: $\mathrm{behaviour} = 0$ (low activity) or $\mathrm{behaviour} = 1$ (active). See eqn 9.
$b(\boldsymbol{s})$	The bathymetric depth (m) in the location defined by $\boldsymbol{s}$. See eqn 13.
β	A linear coefficient in a logistic, distance-decaying, detection-probability model. See eqn 12.
d	The movement step length (m). See eqn 4.
$\delta_{I,I_{i,t}}$	The Kronecker delta. This evaluates to one if the grid cell of coordinate i at time t ($I_{i,t}$) equals grid cell I or 0 otherwise. See eqn 23.
$f(\cdot)$	A probability density function. See eqn 1.
$f(\boldsymbol{s}_{1:T})$	The prior probability density (i.e., the movement process), which is modelled as a discrete-time Markovian process. See eqn 1.
$f(\boldsymbol{s}_{t=1})$	The probability density of the individual's initial state. See eqn 2.
$f(\boldsymbol{s}_t \mid \boldsymbol{s}_{t-1})$	The probability density of movement from the individual's state at time $t-1$ to the location at time t; i.e., the movement model. See eqn 2.
$f(\boldsymbol{s}_t \mid \boldsymbol{y}_{1:T})$	The full marginal distribution of the individual's state at a given time ($\boldsymbol{s}_t$) given all of the data ($\boldsymbol{y}_{1:T}$). This is approximated by particle smoothing.
$f(\boldsymbol{s}_{1:T} \mid \boldsymbol{y}_{1:T})$	The joint probability distribution of the individual's states ($\boldsymbol{s}_{1:T}$) and all data ($\boldsymbol{y}_{1:T}$). See eqn 1.
$f(\boldsymbol{y}_t \mid \boldsymbol{s}_t)$	The probability density of the observations at a given time ($\boldsymbol{y}_t$) given the individual's state at that time ($\boldsymbol{s}_t$); i.e., the likelihood.
$f(\boldsymbol{y}_{1:T} \mid \boldsymbol{s}_{1:T})$	The joint probability density of all observations ($\boldsymbol{y}_{1:T}$) and all states ($\boldsymbol{s}_{1:T}$); i.e., the joint likelihood. See eqn 1.
$f_d(d)$	The probability density of step lengths (d). See eqn 5.
$f_{\Delta\phi}(\Delta\phi)$	The probability density of turning angles ($\Delta\phi$). See eqn 5.
ϕ	A heading (in radians). See eqn 4.
γ	The maximum detection range (m); that is, the distance from a receiver beyond which the detection of an acoustic transmission is assumed impossible. See eqn 12.
$h(\boldsymbol{s}_t, \boldsymbol{r}_k)$	The Euclidean distance between the transmitter, at the location defined by $\boldsymbol{s}_t$, and receiver k (at $\boldsymbol{r}_k = (s_{x;k}, s_{y;k})$). See eqn 12.
I	An index over grid cells. See eqn 23.
J	The Jacobian. See eqn 7.
k_1, k_2	The location parameters of the (truncated) Cauchy distributions, used to model movement step lengths during 'low activity' (k_1) and 'active' (k_2) behavioural states. See eqn 9. We also separately use k

Notation	Description
	as an index over operational receivers (see eqn 11) and k_i to represent the number of time steps over which a behaviour is maintained (see eqn 26).
$\mathrm{lim}_{\mathrm{shallow}}, \mathrm{lim}_{\mathrm{deep}}$	Shallow and deep truncation limits in the depth observation model. See eqn 13.
mobility	The maximum moveable distance (m) between two consecutive time steps. (This is the upper truncation parameter of the truncated Cauchy distribution used to model movement step lengths.) See eqn 9.
$p_{k,t}(\boldsymbol{s}_t)$	The probability of a detection at receiver k at time t, given an acoustic transmission from the location defined by $\boldsymbol{s}_t$. See eqn 11.
P_I	Probability-of-use for grid cell I; that is, the probability that an individual is located in that cell at a randomly chosen time. See eqn 23.
$\boldsymbol{r}_k = (s_{x;k}, s_{y;k})$	Receiver k's two-dimensional location. The expression $\boldsymbol{r}_k = (s_{x;k}, s_{y;k})$ denotes that $\boldsymbol{r}_k$ is a two-dimensional vector with x ($s_{x;k}$) and y ($s_{y;k}$) coordinates. See eqn 12.
$\boldsymbol{s}_t$	The individual's state $\boldsymbol{s}_t = (s_{x,t}, s_{y,t}, \phi_t, \mathrm{behaviour}_t)$. This comprises the individual's two-dimensional location ($s_{x,t}, s_{y,t}$), the heading (ϕ_t) and the behaviour. See eqn 1.
σ_{depth}	The standard deviation of the (truncated) Gaussian distribution of the depth observation model. See eqn 13.
$\sigma_{\Delta\phi}$	The standard deviation of the Gaussian model of turning angle. See eqn 10.
t	The time step index $(1, 2, \dots, T)$. See eqn 1.
T	The final time step. See eqn 1.
θ_1, θ_2	The scale parameters of the (truncated) Cauchy distribution used to model movement step lengths during 'low activity' (θ_1) and 'active' (θ_2) behavioural states. See eqn 9.
μ_{depth}	The mean of the (truncated) Gaussian distribution of the depth observation model. See eqn 13.
$w_{i,t}$	The normalised weight of the ith pair of coordinates at time t. See eqn 23.
$\boldsymbol{y}$	A dataset of observations. In the ACDC algorithm, we consider a combined dataset comprising acoustic and archival (depth) observations; i.e., $\boldsymbol{y} = \{\boldsymbol{y}^{(A)}, \boldsymbol{y}^{(D)}\}$. See eqn 1.
$\boldsymbol{y}^{(A)}$	An $M \times T$ matrix of acoustic observations, which comprise detections (1) and non-detections (0) at each receiver k; i.e., $y_{k,t}^{(A)} \in \{0, 1\}$. A column-vector of acoustic observations at time t is denoted $\boldsymbol{y}_t^{(A)}$. See eqn 11.
$\boldsymbol{y}^{(D)}$	A row vector of depth (m) observations. A single depth observation at time t is denoted $y_t^{(D)}$. See eqn 13.
$\frac{1}{z(\boldsymbol{s}_{t-1})}$	A normalisation constant required to evaluate the probability density of a movement from location $\boldsymbol{s}_{t-1} \rightarrow \boldsymbol{s}_t$. See eqn 8.

Table S4. Parameter values for algorithm implementations. For each algorithm, component sub-model, parameter and parameter setting (1, 2, 3), parameter values are shown. Parameter settings 1, 2 and 3 correspond to the optimal/best-guess, restricted and flexible parameterisations. In analyses A1 and A3, optimal/best-guess algorithm parameterisations were used to evaluate algorithm performance and for the main real-world analyses (Table S2). In analyses A2 and A4, the different parameter settings were used to analyse algorithm sensitivity (Table S2). The COA algorithm depends only on time interval over which COAs are calculated (ΔT). The RSP algorithm depends on a suite of tuning parameters. We trialled three settings for the `er.ad` parameter, which tunes the rate at which uncertainty increases with distance from receivers; the detection range was set to $\gamma = 3000$ m (as in best-guess particle algorithms); and other parameters were set to default values. The particle algorithms (AC, DC and ACDC) depend on movement (Move) and observation (acoustic [AC] and/or depth [DC]) model parameters: $k_1, k_2, \theta_1, \theta_2$ and mobility are the parameters of a truncated, two-state Cauchy model of step length; $\sigma_{\Delta\phi}$ is the standard deviation of a Gaussian model of turning angle; α, β, γ are the parameters of a truncated logistic distance-decaying acoustic detection-probability model; and μ_{depth}, σ_{depth}, $\text{lim}_{\text{shallow}}$ and lim_{deep} are the parameters of a truncated Gaussian depth observation model. For details, see Supplementary Text §4. For a visualisation of particle algorithm sub-models, see Fig. S1.

Algorithm	Sub-model	Parameter	Setting	Value
COA	-	ΔT	1	2 days
			2	1 day
			3	3 days
RSP	-	er.ad	1	500.0
			2	250.0
			3	750.0
AC	Movement (Move)	$\begin{cases}(k_1, \theta_1, \text{mobility}, \sigma_{\Delta\phi})\\(k_2, \theta_2, \text{mobility}, \sigma_{\Delta\phi})\end{cases}$	1	$\begin{cases}(0.0, 5.0, 1095.0, 1.5)\\(5.0, 100.0, 1095.0, 1.5)\end{cases}$
			2	$\begin{cases}(0.0, 5.0, 985.5, 1.5)\\(2.5, 50.0, 985.5, 1.5)\end{cases}$
			3	$\begin{cases}(0.0, 5.0, 1204.5, 1.5)\\(7.5, 150.0, 1204.5, 1.5)\end{cases}$
	Acoustic (AC)	(α, β, γ)	1	$(4.0, -0.0094, 3000.0)$
			2	$(3.0, -0.01175, 2250.0)$
			3	$(5.0, -0.00705, 3750.0)$
DC	Movement (Move)		1	$\begin{cases}(0.0, 5.0, 1095.0, 1.5)\\(5.0, 100.0, 1095.0, 1.5)\end{cases}$

Algorithm	Sub-model	Parameter	Setting	Value
		$\begin{cases}(k_1, \theta_1, \text{mobility}, \sigma_{\Delta\phi}) \\ (k_2, \theta_2, \text{mobility}, \sigma_{\Delta\phi})\end{cases}$	2	$\begin{cases}(0.0, 5.0, 985.5, 1.5) \\ (2.5, 50.0, 985.5, 1.5)\end{cases}$
			3	$\begin{cases}(0.0, 5.0, 1204.5, 1.5) \\ (7.5, 150.0, 1204.5, 1.5)\end{cases}$
	Depth (DC)	$(\mu_{\text{depth}}, \sigma_{\text{depth}}, \text{lim}_{\text{shallow}}, \text{lim}_{\text{deep}})$	1	$(b(\boldsymbol{s}), 100.0, 0.0, 350.0)$
			2	$(b(\boldsymbol{s}), 50.0, 0.0, 350.0)$
			3	$(b(\boldsymbol{s}), 150.0, 0.0, 350.0)$
ACDC	Movement (Move)	$\begin{cases}(k_1, \theta_1, \text{mobility}, \sigma_{\Delta\phi}) \\ (k_2, \theta_2, \text{mobility}, \sigma_{\Delta\phi})\end{cases}$	1	$\begin{cases}(0.0, 5.0, 1095.0, 1.5) \\ (5.0, 100.0, 1095.0, 1.5)\end{cases}$
			2	$\begin{cases}(0.0, 5.0, 985.5, 1.5) \\ (2.5, 50.0, 985.5, 1.5)\end{cases}$
			3	$\begin{cases}(0.0, 5.0, 1204.5, 1.5) \\ (7.5, 150.0, 1204.5, 1.5)\end{cases}$
	Acoustic (AC)	(α, β, γ)	1	$(4.0, -0.0094, 3000.0)$
			2	$(3.0, -0.01175, 2250.0)$
			3	$(5.0, -0.00705, 3750.0)$
	Depth (DC)	$(\mu_{\text{depth}}, \sigma_{\text{depth}}, \text{lim}_{\text{shallow}}, \text{lim}_{\text{deep}})$	1	$(b(\boldsymbol{s}), 100.0, 0.0, 350.0)$
			2	$(b(\boldsymbol{s}), 50.0, 0.0, 350.0)$
			3	$(b(\boldsymbol{s}), 150.0, 0.0, 350.0)$

Table S5. Summary of modelled flapper skate. For each individual, the acoustic and archival (depth) tag identifiers are provided for reference to previous publications (e.g., refs (*32*, *35*)). Individual sex, maturation status and residency labels assigned by ref. (*32*) are also provided. Residency labels comprise the residency category (non [N], short-term [S] or long-term [L] resident) and the total number of days with detections. (For details, see ref. (*32*).) In the present study, only a subset of (N) months with sufficient data (listed) were analysed for most individuals.

Individual	Acoustic	Archival	Sex	Maturity	Resident	Months
13	249	1558	F	Immature	L185	(1) 04-2016
17	244	1536	M	Mature	N38	(1) 09-2016
20	242	1538	F	Immature	S24	(2) 04-2016, 05-2016
25	555	1547	F	Mature	L256	(14) 04-2016, 05-2016, 06-2016, 07-2016, 08-2016, 09-2016, 10-2016, 11-2016, 12-2016, 01-2017, 02-2017, 03-2017, 04-2017, 05-2016
27	563	1520	M	Mature	S64	(3) 04-2016, 05-2016, 06-2016
28	560	1522	F	Immature	S150	(7) 05-2016, 06-2016, 09-2016, 10-2016, 01-2016, 02-2016, 03-2016
29	542	1523	M	Mature	N34	(1) 04-2016
32	549	1548	M	Mature	S38	(3) 10-2016, 02-2017, 03-2017
35	540	1509	F	Immature	L194	(12) 04-2016, 05-2016, 06-2016, 07-2016, 08-2016, 09-2016, 10-2016, 11-2016, 12-2016, 01-2016, 02-2017, 03-2017
36	543	1512	F	Mature	S130	(3) 04-2016, 05-2016, 06-2016
38	547	1507	F	Mature	S67	(1) 04-2016

REFERENCES AND NOTES

1. C. M. Duarte, S. Agusti, E. Barbier, G. L. Britten, J. C. Castilla, J.-P. Gattuso, R. W. Fulweiler, T. P. Hughes, N. Knowlton, C. E. Lovelock, H. K. Lotze, M. Predragovic, E. Poloczanska, C. Roberts, B. Worm, Rebuilding marine life. *Nature* **580**, 39–51 (2020).

2. D. J. McCauley, M. L. Pinsky, S. R. Palumbi, J. A. Estes, F. H. Joyce, R. R. Warner, Marine defaunation: Animal loss in the global ocean. *Science* **347**, 1255641 (2015).

3. N. Pacoureau, C. L. Rigby, P. M. Kyne, R. B. Sherley, H. Winker, J. K. Carlson, S. V. Fordham, R. Barreto, D. Fernando, M. P. Francis, R. W. Jabado, K. B. Herman, K.-M. Liu, A. D. Marshall, R. A. Pollom, E. V. Romanov, C. A. Simpfendorfer, J. S. Yin, H. K. Kindsvater, N. K. Dulvy, Half a century of global decline in oceanic sharks and rays. *Nature* **589**, 567–571 (2021).

4. N. K. Dulvy, N. Pacoureau, J. H. Matsushiba, H. F. Yan, W. J. VanderWright, C. L. Rigby, B. Finucci, C. S. Sherman, R. W. Jabado, J. K. Carlson, R. A. Pollom, P. Charvet, C. M. Pollock, C. Hilton-Taylor, C. A. Simpfendorfer, Ecological erosion and expanding extinction risk of sharks and rays. *Science* **386**, eadn1477 (2024).

5. G. M. Mace, M. Barrett, N. D. Burgess, S. E. Cornell, R. Freeman, M. Grooten, A. Purvis, Aiming higher to bend the curve of biodiversity loss. *Nat. Sustain.* **1**, 448–451 (2018).

6. K. Grorud-Colvert, J. Sullivan-Stack, C. Roberts, V. Constant, B. Horta e Costa, E. P. Pike, N. Kingston, D. Laffoley, E. Sala, J. Claudet, A. M. Friedlander, D. A. Gill, S. E. Lester, J. C. Day, E. J. Gonçalves, G. N. Ahmadia, M. Rand, A. Villagomez, N. C. Ban, G. G. Gurney, A. K. Spalding, N. J. Bennett, J. Briggs, L. E. Morgan, R. Moffitt, M. Deguignet, E. K. Pikitch, E. S. Darling, S. Jessen, S. O. Hameed, G. Di Carlo, P. Guidetti, J. M. Harris, J. Torre, Z. Kizilkaya, T. Agardy, P. Cury, N. J. Shah, K. Sack, L. Cao, M. Fernandez, J. Lubchenco, The MPA Guide: A framework to achieve global goals for the ocean. *Science* **373**, eabf0861 (2021).

7. J. A. Turner, M. Starkey, N. K. Dulvy, F. Hawkins, L. Mair, A. Serckx, T. Brooks, B. Polidoro, S. H. M. Butchart, K. Carpenter, M. Epps, R. W. Jabado, N. B. W. Macfarlane, L. Bennun,

Targeting ocean conservation outcomes through threat reduction. *npj Ocean Sustain.* **3**, 4 (2024).

8. F. Ferretti, D. Curnick, K. Liu, E. V. Romanov, B. A. Block, Shark baselines and the conservation role of remote coral reef ecosystems. *Sci. Adv.* **4**, eaaq0333 (2018).

9. A. Grüss, D. M. Kaplan, S. Guénette, C. M. Roberts, L. W. Botsford, Consequences of adult and juvenile movement for marine protected areas. *Biol. Conserv.* **144**, 692–702 (2011).

10. G. C. Hays, H. Bailey, S. J. Bograd, W. D. Bowen, C. Campagna, R. H. Carmichael, P. Casale, A. Chiaradia, D. P. Costa, E. Cuevas, P. J. Nico de Bruyn, M. P. Dias, C. M. Duarte, D. C. Dunn, P. H. Dutton, N. Esteban, A. Friedlaender, K. T. Goetz, B. J. Godley, P. N. Halpin, M. Hamann, N. Hammerschlag, R. Harcourt, A.-L. Harrison, E. L. Hazen, M. R. Heupel, E. Hoyt, N. E. Humphries, C. Y. Kot, J. S. E. Lea, H. Marsh, S. M. Maxwell, C. R. McMahon, G. Notarbartolo di Sciara, D. M. Palacios, R. A. Phillips, D. Righton, G. Schofield, J. A. Seminoff, C. A. Simpfendorfer, D. W. Sims, A. Takahashi, M. J. Tetley, M. Thums, P. N. Trathan, S. Villegas-Amtmann, R. S. Wells, S. D. Whiting, N. E. Wildermann, A. M. M. Sequeira, Translating marine animal tracking data into conservation policy and management. *Trends Ecol. Evol.* **34**, 459–473 (2019).

11. N. E. Hussey, S. T. Kessel, K. Aarestrup, S. J. Cooke, P. D. Cowley, A. T. Fisk, R. G. Harcourt, K. N. Holland, S. J. Iverson, J. F. Kocik, J. E. Mills Flemming, F. G. Whoriskey, Aquatic animal telemetry: A panoramic window into the underwater world. *Science* **348**, 1255642 (2015).

12. B. R. Mate, V. Y. Ilyashenko, A. L. Bradford, V. V. Vertyankin, G. A. Tsidulko, V. V. Rozhnov, L. M. Irvine, Critically endangered western gray whales migrate to the eastern North Pacific. *Biol. Lett.* **11**, 20150071 (2015).

13. Y.-C. Chan, T. L. Tibbitts, T. Lok, C. J. Hassell, H.-B. Peng, Z. Ma, Z. Zhang, T. Piersma, Filling knowledge gaps in a threatened shorebird flyway through satellite tracking. *J. Appl. Ecol.* **56**, 2305–2315 (2019).

14. I. D. Jonsen, T. A. Patterson, D. P. Costa, P. D. Doherty, B. J. Godley, W. J. Grecian, C. Guinet, X. Hoenner, S. S. Kienle, P. W. Robinson, S. C. Votier, S. Whiting, M. J. Witt, M. A. Hindell, R. G. Harcourt, C. R. McMahon, A continuous-time state-space model for rapid quality control of argos locations from animal-borne tags. *Mov. Ecol.* **8**, 31 (2020).

15. J. K. Matley, N. V. Klinard, A. P. Barbosa Martins, K. Aarestrup, E. Aspillaga, S. J. Cooke, P. D. Cowley, M. R. Heupel, C. G. Lowe, S. K. Lowerre-Barbieri, H. Mitamura, J.-S. Moore, C. A. Simpfendorfer, M. J. W. Stokesbury, M. D. Taylor, E. B. Thorstad, C. S. Vandergoot, A. T. Fisk, Global trends in aquatic animal tracking with acoustic telemetry. *Trends Ecol. Evol.* **37**, 79–94 (2022).

16. J. K. Matley, N. V. Klinard, S. M. Larocque, M. F. McLean, J. W. Brownscombe, G. D. Raby, V. M. Nguyen, A. P. Barbosa Martins, Making the most of aquatic animal tracking: A review of complementary methods to bolster acoustic telemetry. *Rev. Fish Biol. Fish.* **33**, 35–54 (2023).

17. S. Kraft, M. Gandra, R. J. Lennox, J. Mourier, A. C. Winkler, D. Abecasis, Residency and space use estimation methods based on passive acoustic telemetry data. *Mov. Ecol.* **11**, 12 (2023).

18. E. Lavender, S. Biber, J. Illian, M. James, P. J. Wright, J. Thorburn, S. Smout, An integrative modelling framework for passive acoustic telemetry. *Methods Ecol. Evol.* **14**, 2626–2638 (2023).

19. E. Lavender, A. Scheidegger, C. Albert, S. W. Biber, J. Illian, J. Thorburn, S. Smout, H. Moor, Particle algorithms for animal movement modelling in receiver arrays. *Methods Ecol. Evol.* **16**, 1808–1819 (2025).

20. C. A. Simpfendorfer, M. R. Heupel, R. E. Hueter, Estimation of short-term centers of activity from an array of omnidirectional hydrophones and its use in studying animal movements. *Can. J. Fish. Aquat. Sci.* **59**, 23–32 (2002).

21. V. Udyawer, R. G. Dwyer, X. Hoenner, R. C. Babcock, S. Brodie, H. A. Campbell, R. G. Harcourt, C. Huveneers, F. R. A. Jaine, C. A. Simpfendorfer, M. D. Taylor, M. R. Heupel, A

standardised framework for analysing animal detections from automated tracking arrays. *Anim. Biotelemetry* **6**, 17 (2018).

22. Y. Niella, H. Flávio, A. F. Smoothey, K. Aarestrup, M. D. Taylor, V. M. Peddemors, R. Harcourt, Refined shortest paths (RSP): Incorporation of topography in space use estimation from node-based telemetry data. *Methods Ecol. Evol.* **11**, 1733–1742 (2020).

23. P. Afonso, J. Fontes, K. N. Holland, R. S. Santos, Social status determines behaviour and habitat usage in a temperate parrotfish: Implications for marine reserve design. *Mar. Ecol. Prog. Ser.* **359**, 215–227 (2008).

24. N. J. Hostetter, J. A. Royle, Movement-assisted localization from acoustic telemetry data. *Mov. Ecol.* **8**, 15 (2020).

25. T. A. Patterson, L. Thomas, C. Wilcox, O. Ovaskainen, J. Matthiopoulos, State–space models of individual animal movement. *Trends Ecol. Evol.* **23**, 87–94 (2008).

26. M. W. Pedersen, K. C. Weng, Estimating individual animal movement from observation networks. *Methods Ecol. Evol.* **4**, 920–929 (2013).

27. J. Alós, M. Palmer, S. Balle, R. Arlinghaus, Bayesian state-space modelling of conventional acoustic tracking provides accurate descriptors of home range behavior in a small-bodied coastal fish species. *PLOS ONE* **11**, e0154089 (2016).

28. E. Lavender, A. Scheidegger, C. Albert, S. Biber, J. Illian, J. Thorburn, S. Smout, H. Moor, patter: Particle algorithms for animal tracking in R and Julia. *Methods Ecol. Evol.* **16**, 1609–1616 (2025).

29. J. R. Ellis, S. R. McCully-Philipps, D. Sims, R. H. L. Walls, J. Cheok, D. Derrick, N. K. Dulvy, *Dipturus intermedius*. The IUCN Red List of Threatened Species 2021. e. T18903491A68783461 (2021).

30. K. Brander, Disappearance of common skate *Raia batis* from Irish Sea. *Nature* **290**, 48–49 (1981).

31. R. J. Kynoch, R. J. Fryer, F. C. Neat, A simple technical measure to reduce bycatch and discard of skates and sharks in mixed-species bottom-trawl fisheries. *ICES J. Mar. Sci.* **72**, 1861–1868 (2015).

32. E. Lavender, D. Aleynik, J. Dodd, J. Illian, M. James, P. J. Wright, S. Smout, J. Thorburn, Movement patterns of a Critically Endangered elasmobranch (*Dipturus intermedius*) in a Marine Protected Area. *Aquat. Conserv. Mar. Freshw. Ecosyst.* **32**, 348–365 (2022).

33. E. Lavender, D. Aleynik, J. Dodd, J. Illian, M. James, J. Smout, J. Thorburn, Benthic animal-borne sensors and citizen science combine to validate ocean modelling. *Sci. Rep.* **12**, 16613 (2022).

34. J. Thorburn, P. J. Wright, E. Lavender, J. Dodd, F. Neat, J. G. A. Martin, C. Lynam, M. James, Seasonal and ontogenetic variation in depth use by a Critically Endangered benthic elasmobranch and its implications for spatial management. *Front. Mar. Sci.* **8**, 656368 (2021).

35. E. Lavender, D. Aleynik, J. Dodd, J. Illian, M. James, P. J. Wright, S. Smout, J. Thorburn, Environmental cycles and individual variation in the vertical movements of a benthic elasmobranch. *Mar. Biol.* **168**, 164 (2021).

36. E. Lavender, D. Aleynik, J. Dodd, J. Illian, M. James, P. J. Wright, S. Smout, J. Thorburn, Behavioural responses of a large, benthic elasmobranch to catch-and-release angling. *Front. Mar. Sci.* **9**, 864344 (2022).

37. J. Dodd, J. M. Baxter, D. W. Donnan, B. D. James, E. Lavender, C. A. McSorley, A. O. M. Mogg, J. A. Thorburn, First report of an egg nursery for the Critically Endangered flapper skate *Dipturus intermedius* (Rajiformes: Rajidae). *Aquat. Conserv. Mar. Freshw. Ecosyst.* **32**, 1647–1659 (2022).

38. I. Sousa, J. Baeyaert, J. M. S. Gonçalves, K. Erzini, Preliminary insights into the spatial ecology and movement patterns of a regionally critically endangered skate (*Rostroraja alba*) associated with a marine protected area. *Mar. Freshw. Behav. Physiol.* **52**, 283–299 (2019).

39. S. Kraft, A. C. Winkler, D. Abecasis, Horizontal and vertical movements of the critically endangered *Rostroraja alba* in a coastal marine protected area. *Aquat. Conserv. Mar. Freshw. Ecosyst.* **34**, e4204 (2024).

40. J. Alós, K. Aarestrup, D. Abecasis, P. Afonso, A. Alonso-Fernandez, E. Aspillaga, M. Barcelo-Serra, J. Bolland, M. Cabanellas-Reboredo, R. Lennox, R. McGill, A. Özgül, J. Reubens, D. Villegas-Ríos, Toward a decade of ocean science for sustainable development through acoustic animal tracking. *Glob. Change Biol.* **28**, 5630–5653 (2022).

41. J. Cariboni, D. Gatelli, R. Liska, A. Saltelli, The role of sensitivity analysis in ecological modelling. *Ecol. Model.* **203**, 167–182 (2007).

42. A. M. Schlaff, M. R. Heupel, C. A. Simpfendorfer, Influence of environmental factors on shark and ray movement, behaviour and habitat use: A review. *Rev. Fish Biol. Fish.* **24**, 1089–1103 (2014).

43. S. Andrzejaczek, T. C. D. Lucas, M. C. Goodman, N. E. Hussey, A. J. Armstrong, A. Carlisle, D. M. Coffey, A. C. Gleiss, C. Huveneers, D. M. P. Jacoby, M. G. Meekan, J. Mourier, L. R. Peel, K. Abrantes, A. S. Afonso, M. J. Ajemian, B. N. Anderson, S. D. Anderson, G. Araujo, A. O. Armstrong, P. Bach, A. Barnett, M. B. Bennett, N. A. Bezerra, R. Bonfil, A. M. Boustany, H. D. Bowlby, I. Branco, C. D. Braun, E. J. Brooks, J. Brown, P. J. Burke, P. Butcher, M. Castleton, T. K. Chapple, O. Chateau, M. Clarke, R. Coelho, E. Cortes, L. I. E. Couturier, P. D. Cowley, D. A. Croll, J. M. Cuevas, T. H. Curtis, L. Dagorn, J. J. Dale, R. Daly, H. Dewar, P. D. Doherty, A. Domingo, A. D. M. Dove, M. Drew, C. L. Dudgeon, C. A. J. Duffy, R. G. Elliott, J. R. Ellis, M. V. Erdmann, T. J. Farrugia, L. C. Ferreira, F. Ferretti, J. D. Filmalter, B. Finucci, C. Fischer, R. Fitzpatrick, F. Forget, K. Forsberg, M. P. Francis, B. R. Franks, A. J. Gallagher, F. Galvan-Magana, M. L. García, T. F. Gaston, B. M. Gillanders, M. J. Gollock, J. R. Green, S. Green, C. A. Griffiths, N. Hammerschlag, A. Hasan, L. A. Hawkes, F. Hazin, M. Heard, A. Hearn, K. J. Hedges, S. M. Henderson, J. Holdsworth, K. N. Holland, L. A. Howey, R. E. Hueter, N. E. Humphries, M. Hutchinson, F. R. A. Jaine, S. J. Jorgensen, P. E. Kanive, J. Labaja, F. O. Lana, H. Lassauce, R. S. Lipscombe, F. Llewellyn, B. C. L. Macena, R. Mambrasar, J. D. McAllister, S. R. McCully Phillips, F. McGregor, M. N. McMillan, L. M. McNaughton, S. A. Mendonça, C. G. Meyer, M. Meyers, J. A. Mohan, J.

C. Montgomery, G. Mucientes, M. K. Musyl, N. Nasby-Lucas, L. J. Natanson, J. B. O'Sullivan, P. Oliveira, Y. P. Papastamtiou, T. A. Patterson, S. J. Pierce, N. Queiroz, C. A. Radford, A. J. Richardson, A. J. Richardson, D. Righton, C. A. Rohner, M. A. Royer, R. A. Saunders, M. Schaber, R. J. Schallert, M. C. Scholl, A. C. Seitz, J. M. Semmens, E. Setyawan, B. D. Shea, R. A. Shidqi, G. L. Shillinger, O. N. Shipley, M. S. Shivji, A. B. Sianipar, J. F. Silva, D. W. Sims, G. B. Skomal, L. L. Sousa, E. J. Southall, J. L. Y. Spaet, K. M. Stehfest, G. Stevens, J. D. Stewart, J. A. Sulikowski, I. Syakurachman, S. R. Thorrold, M. Thums, D. Tickler, M. T. Tolloti, K. A. Townsend, P. Travassos, J. P. Tyminski, J. J. Vaudo, D. Veras, L. Wantiez, S. B. Weber, R. J. D. Wells, K. C. Weng, B. M. Wetherbee, J. E. Williamson, M. J. Witt, S. Wright, K. Zilliacus, B. A. Block, D. J. Curnick, Diving into the vertical dimension of elasmobranch movement ecology. *Sci. Adv.* **8**, eabo1754 (2022).

44. K. Whoriskey, E. G. Martins, M. Auger-Méthé, L. F. G. Gutowsky, R. J. Lennox, S. J. Cooke, M. Power, J. Mills Flemming, Current and emerging statistical techniques for aquatic telemetry data: A guide to analysing spatially discrete animal detections. *Methods Ecol. Evol.* **10**, 935–948 (2019).

45. D. M. P. Jacoby, A. T. Piper, What acoustic telemetry can and cannot tell us about fish biology. *J. Fish Biol.* **106**, 1260–1284 (2023).

46. T. H. Selby, K. M. Hart, B. J. Smith, C. G. Pollock, Z. Hillis-Starr, M. K. Oli, Juvenile hawksbill residency and habitat use within a Caribbean marine protected area. *Endang. Species Res.* **40**, 53–64 (2019).

47. L. P. Griffin, G. A. Casselberry, K. M. Hart, A. Jordaan, S. L. Becker, A. J. Novak, B. M. DeAngelis, C. G. Pollock, I. Lundgren, Z. Hillis-Starr, A. J. Danylchuk, G. B. Skomal, A novel framework to predict relative habitat selection in aquatic systems: Applying machine learning and resource selection functions to acoustic telemetry data from multiple shark species. *Front. Mar. Sci.* **8**, 631262 (2021).

48. C. N. Ehler, Two decades of progress in Marine Spatial Planning. *Mar. Policy* **132**, 104134 (2021).

49. J. A. Sulikowski, N. Hammerschlag, A novel intrauterine satellite transmitter to identify parturition in large sharks. *Sci. Adv.* **9**, eadd6340 (2023).

50. S. T. Kessel, S. J. Cooke, M. R. Heupel, N. E. Hussey, C. A. Simpfendorfer, S. Vagle, A. T. Fisk, A review of detection range testing in aquatic passive acoustic telemetry studies. *Rev. Fish Biol. Fish.* **24**, 199–218 (2014).

51. M. W. Pedersen, D. Righton, U. H. Thygesen, K. H. Andersen, H. Madsen, Geolocation of North Sea cod (*Gadus morhua*) using hidden Markov models and behavioural switching. *Can. J. Fish. Aquat. Sci.* **65**, 2367–2377 (2008).

52. M. M. Graham, A. Storkey, Continuously tempered Hamiltonian Monte Carlo. arXiv:1704.03338 [stat.CO] (2017).

53. E. Lavender, A. Scheidegger, H. Moor, C. Albert, State-space models and inference approaches for aquatic animal tracking with passive acoustic telemetry and biologging sensors. EcoEvoRXiv [Preprint]. https://ecoevorxiv.org/repository/view/9215.

54. L. P. Griffin, G. A. Casselberry, S. K. Lowerre-Barbieri, A. Acosta, A. J. Adams, S. J. Cooke, A. Filous, C. Friess, T. L. Guttridge, N. Hammerschlag, V. Heim, D. Morley, M. J. Rider, G. B. Skomal, M. J. Smukall, A. J. Danylchuk, J. W. Brownscombe, Predator–prey landscapes of large sharks and game fishes in the Florida Keys. *Ecol. Appl.* **32**, e2584 (2022).

55. R. Harcourt, A. M. M. Sequeira, X. Zhang, F. Roquet, K. Komatsu, M. Heupel, C. McMahon, F. Whoriskey, M. Meekan, G. Carroll, S. Brodie, C. Simpfendorfer, M. Hindell, I. Jonsen, D. P. Costa, B. Block, M. Muelbert, B. Woodward, M. Weise, K. Aarestrup, M. Biuw, L. Boehme, S. J. Bograd, D. Cazau, J.-B. Charrassin, S. J. Cooke, P. Cowley, P. J. N. de Bruyn, T. du Dot, C. Duarte, V. M. Eguíluz, L. C. Ferreira, J. Fernández-Gracia, K. Goetz, Y. Goto, C. Guinet, M. Hammill, G. C. Hays, E. L. Hazen, L. A. Hückstädt, C. Huveneers, S. Iverson, S. A. Jaaman, K. Kittiwattanawong, K. M. Kovacs, C. Lydersen, T. Moltmann, M. Naruoka, L. Phillips, B. Picard, N. Queiroz, G. Reverdin, K. Sato, D. W. Sims, E. B. Thorstad, M. Thums, A. M. Treasure, A. W. Trites, G. D. Williams, Y. Yonehara, M. A. Fedak, Animal-borne telemetry: An integral component of the ocean observing toolkit. *Front. Mar. Sci.* **6**, 326 (2019).

56. G. T. Crossin, M. R. Heupel, C. M. Holbrook, N. E. Hussey, S. K. Lowerre-Barbieri, V. M. Nguyen, G. D. Raby, S. J. Cooke, Acoustic telemetry and fisheries management. *Ecol. Appl.* **27**, 1031–1049 (2017).

57. R. J. Lennox, P. Afonso, K. Birnie-Gauvin, L. S. Dahlmo, C. I. Nilsen, R. Arlinghaus, S. J. Cooke, A. T. Souza, I. Jarić, M. Prchalová, M. Říha, S. Westrelin, W. Twardek, E. Aspillaga, S. Kraft, M. Šmejkal, H. Baktoft, T. Brodin, G. Hellström, D. Villegas-Ríos, K. W. Vollset, T. Adam, L. K. Sortland, M. G. Bertram, M. Crossa, E. F. Vogel, N. Gillies, J. Reubens, Electronic tagging and tracking aquatic animals to understand a world increasingly shaped by a changing climate and extreme weather events. *Can. J. Fish. Aquat. Sci.* **81**, 326–339 (2024).

58. M. Espinoza, T. J. Farrugia, C. G. Lowe, Habitat use, movements and site fidelity of the gray smooth-hound shark (*Mustelus californicus* Gill 1863) in a newly restored southern California estuary. *J. Exp. Mar. Biol. Ecol.* **401**, 63–74 (2011).

59. E. C. Ingram, R. M. Cerrato, K. J. Dunton, M. G. Frisk, Endangered Atlantic Sturgeon in the New York Wind Energy Area: Implications of future development in an offshore wind energy site. *Sci. Rep.* **9**, 12432 (2019).

60. R Core Team, *R: A Language and Environment for Statistical Computing, Version 4.3.1* (R Foundation for Statistical Computing, 2023).

61. E. Lavender, A. Scheidegger, C. Albert, S. W. Biber, J. Brodersen, D. Aleynik, G. Cole, J. Dodd, P. J. Wright, J. Illian, M. James, S. Smout, J. Thorburn, H. Moor, Animal tracking with particle algorithms informs protected area design. Zenodo. doi: 10.5281/zenodo.14805161 (2025).

62. S. Benjamins, J. Dodd, J. Thorburn, V. A. Milway, R. Campbell, D. M. Bailey, Evaluating the potential of photo-identification as a monitoring tool for flapper skate (*Dipturus intermedius*). *Aquat. Conserv. Mar. Freshw. Ecosyst.* **28**, 1360–1373 (2018).

63. J. A. Howe, R. Anderton, R. Arosio, D. Dove, T. Bradwell, P. Crump, R. Cooper, A. Cocuccio, The seabed geomorphology and geological structure of the Firth of Lorn, western

Scotland, UK, as revealed by multibeam echo-sounder survey. *Earth Environ. Sci. Trans. R. Soc. Edinb.* **105**, 273–284 (2014).

64. Global Administrative Areas, GADM database of global administrative areas, version 4.0 (2022). www.gadm.org.

65. International Hydrographic Organization, *International Hydrographic Organization Standards for Hydrographic Surveys. IHO Publication No. 44* (International Hydrographic Organization, Principauté de Monaco, 2020).

66. D. Aleynik, A. C. Dale, M. Porter, K. Davidson, A high resolution hydrodynamic model system suitable for novel harmful algal bloom modelling in areas of complex coastline and topography. *Harmful Algae* **53**, 102–117 (2016).

67. K. Davidson, C. Whyte, D. Aleynik, A. Dale, S. Gontarek, A. A. Kurekin, S. McNeill, P. I. Miller, M. Porter, R. Saxon, S. Swan, HABreports: Online early warning of harmful algal and biotoxin risk for the Scottish shellfish and finfish aquaculture industries. *Front. Mar. Sci.* **8**, 631732 (2021).

68. C. Armstrong, J. A. Howe, A. Dale, C. Allen, Bathymetric observations of an extreme tidal flow: Approaches to the Gulf of Corryvreckan, western Scotland, UK. *Cont. Shelf Res.* **217**, 104347 (2021).

69. C. A. Klöcker, "From transmissions to detections: The impact of environmental variation on acoustic detection probability and its implications for spatial management in a Marine Protected Area," thesis, University of St Andrews, St Andrews, UK (2019).

70. E. Lavender, {patter}: Particle algorithms for animal tracking in R, version 1.0.0 (2024). https://github.com/edwardlavender/patter.

71. J. Bezanson, A. Edelman, S. Karpinski, V. B. Shah, Julia: A fresh approach to numerical computing. *SIAM Rev.* **59**, 65–98 (2017).

72. G. V. Lauder, V. Di Santo, "6—Swimming mechanics and energetics of elasmobranch fishes" in *Fish Physiology*, R. E. Shadwick, A. P. Farrell, C. J. Brauner, Eds. (Academic Press, 2015), vol. 34, pp. 219–253.

73. G. R. Parsons, Metabolism and swimming efficiency of the bonnethead shark *Sphyrna tiburo*. *Mar. Biol.* **104**, 363–367 (1990).

74. G. R. Parsons, J. K. Carlson, Physiological and behavioral responses to hypoxia in the bonnethead shark, *Sphyrna tiburo*: Routine swimming and respiratory regulation. *Fish Physiol. Biochem.* **19**, 189–196 (1998).

75. M. J. Waller, N. Queiroz, I. da Costa, T. Cidade, B. Loureiro, F. C. Womersley, J. Fontes, P. Afonso, B. C. L. Macena, A. Loveridge, N. E. Humphries, E. J. Southall, D. W. Sims, Direct measurement of cruising and burst swimming speeds of the shortfin mako shark (*Isurus oxyrinchus*) with estimates of field metabolic rate. *J. Fish Biol.* **103**, 864–883 (2023).

76. P. Motta, M. L. Habegger, A. Lang, R. Hueter, J. Davis, Scale morphology and flexibility in the shortfin mako *Isurus oxyrinchus* and the blacktip shark *Carcharhinus limbatus*. *J. Morphol.* **273**, 1096–1110 (2012).

77. J. E. Fontanella, F. E. Fish, E. I. Barchi, R. Campbell-Malone, R. H. Nichols, N. K. DiNenno, J. T. Beneski, Two- and three-dimensional geometries of batoids in relation to locomotor mode. *J. Exp. Mar. Biol. Ecol.* **446**, 273–281 (2013).

78. R. T. Graham, M. J. Witt, D. W. Castellanos, F. Remolina, S. Maxwell, B. J. Godley, L. A. Hawkes, Satellite Tracking of Manta Rays Highlights Challenges to Their Conservation. *PLOS ONE* **7**, e36834 (2012).

79. K. Yano, F. Sato, T. Takahashi, Observations of mating behavior of the manta ray, *Manta birostris*, at the Ogasawara Islands, Japan. *Ichthyol. Res.* **46**, 289–296 (1999).

80. F. E. Fish, A. Kolpas, A. Crossett, M. A. Dudas, K. W. Moored, H. Bart-Smith, Kinematics of swimming of the manta ray: Three-dimensional analysis of open-water maneuverability. *J. Exp. Biol.* **221** (Pt. 6), jeb166041 (2018).

81. V. Fong, S. L. Hoffmann, J. H. Pate, Using drones to assess volitional swimming kinematics of manta ray behaviors in the wild. *Drones* **6**, 111 (2022).

82. F. Fish, H. Dong, J. Zhu, H. Bart-Smith, Kinematics and hydrodynamics of mobuliform swimming: Oscillatory winged propulsion by large pelagic batoids. *Mar. Technol. Soc. J.* **51**, 35–47 (2017).

83. F. E. Fish, J. L. Hoffman, Stability design and response to waves by batoids. *Integr. Comp. Biol.* **55**, 648–661 (2015).

84. J. M. Parson, F. E. Fish, A. J. Nicastro, Turning performance of batoids: Limitations of a rigid body. *J. Exp. Mar. Biol. Ecol.* **402**, 12–18 (2011).

85. L. Rosenberger, M. Westneat, Functional morphology of undulatory pectoral fin locomotion in the Stingray *Taeniura lymma* (Chondrichthyes: Dasyatidae). *J. Exp. Biol.* **202**, 3523–3539 (1999).

86. E. L. Blevins, G. V. Lauder, Rajiform locomotion: Three-dimensional kinematics of the pectoral fin surface during swimming in the freshwater stingray *Potamotrygon orbignyi*. *J. Exp. Biol.* **215**, 3231–3241 (2012).

87. D. M. Koester, C. P. Spirito, Punting: An unusual mode of locomotion in the little skate, *Leucoraja erinacea* (Chondrichthyes: Rajidae). *Copeia* **2003**, 553–561 (2003).

88. D. Queirolo, E. Gaete, I. Montenegro, M. C. Soriguer, K. Erzini, Behaviour of fish by-catch in the mouth of a crustacean trawl. *J. Fish Biol.* **80**, 2517–2527 (2012).

89. V. Di Santo, E. L. Blevins, G. V. Lauder, Batoid locomotion: Effects of speed on pectoral fin deformation in the little skate, *Leucoraja erinacea*. *J. Exp. Biol.* **220**, 705–712 (2017).

90. S. M. Bayse, M. V. Pol, P. He, Fish and squid behaviour at the mouth of a drop-chain trawl: Factors contributing to capture or escape. *ICES J. Mar. Sci.* **73**, 1545–1556 (2016).

91. T. H. Selby, K. M. Hart, I. Fujisaki, B. J. Smith, C. J. Pollock, Z. Hillis-Starr, I. Lundgren, M. K. Oli, Can you hear me now? Range-testing a submerged passive acoustic receiver array in a Caribbean coral reef habitat. *Ecol. Evol.* **6**, 4823–4835 (2016).

92. E. R. Winter, A. M. Hindes, S. Lane, J. R. Britton, Detection range and efficiency of acoustic telemetry receivers in a connected wetland system. *Hydrobiologia* **848**, 1825–1836 (2021).

93. M. Long, A. Jordaan, T. Castro-Santos, Environmental factors influencing detection efficiency of an acoustic telemetry array and consequences for data interpretation. *Anim. Biotelemetry* **11**, 18 (2023).

94. T. A. Hayden, C. M. Holbrook, T. R. Binder, J. M. Dettmers, S. J. Cooke, C. S. Vandergoot, C. C. Krueger, Probability of acoustic transmitter detections by receiver lines in Lake Huron: Results of multi-year field tests and simulations. *Anim. Biotelemetry* **4**, 19 (2016).

95. N. V. Klinard, E. A. Halfyard, J. K. Matley, A. T. Fisk, T. B. Johnson, The influence of dynamic environmental interactions on detection efficiency of acoustic transmitters in a large, deep, freshwater lake. *Anim. Biotelemetry* **7**, 17 (2019).

96. V. J. Wearmouth, D. W. Sims, Movement and behaviour patterns of the critically endangered common skate *Dipturus batis* revealed by electronic tagging. *J. Exp. Mar. Biol. Ecol.* **380**, 77–87 (2009).

97. E. Lavender, “Modelling the movements of flapper skate (*Dipturus intermedius*) in relation to a Scottish Marine Protected Area,” thesis, University of St. Andrews, St Andrews, UK (2022).

98. A. Wheeler, *The Fishes of the British Isles and NW Europe* (MacMillan & Co. Ltd., 1969).

99. S. Brown-Vuillemin, T. Barreau, J.-M. Caraguel, S. P. Iglésias, Trophic ecology and ontogenetic diet shift of the blue skate (*Dipturus cf. flossada*). *J. Fish Biol.* **97**, 515–526 (2020).

100. A. Doucet, A. Johansen, “A tutorial on particle filtering and smoothing: Fifteen years later” in *The Oxford Handbook of Nonlinear Filtering*, D. Crisan, B. Rozovskii, Eds. (Oxford Univ. Press, 2009).

101. A. Baddeley, R. Turner, spatstat: An R package for analyzing spatial point patterns. *J. Stat. Softw.* **12**, 1–42 (2005).

102. B. J. Worton, Kernel methods for estimating the utilization distribution in home-range studies. *Ecology* **70**, 164–168 (1989).

103. S. N. Wood, *Generalized Additive Models: An Introduction with R* (Chapman and Hall/CRC, 2017).
